# Development of photo- and chemo-stable near-infrared-emitting dyes: linear-shape benzo-rosol and its derivatives as unique ratiometric bioimaging platforms†

**DOI:** 10.1039/d0sc03314f

**Published:** 2020-08-06

**Authors:** Mingchong Dai, Ye Jin Reo, Chang Wook Song, Yun Jae Yang, Kyo Han Ahn

**Affiliations:** Department of Chemistry, Pohang University of Science and Technology (POSTECH) 77 Cheongam-Ro, Nam-Gu Pohang Gyungbuk 37673 Republic of Korea ahn@postech.ac.kr

## Abstract

Microscopic imaging aided with fluorescent probes has revolutionized our understanding of biological systems. Organic fluorophores and probes thus continue to evolve for bioimaging applications. Fluorophores such as cyanines and hemicyanines emit in the near-infrared (NIR) region and thus allow deeper imaging with minimal autofluorescence; however, they show limited photo- and chemo-stability, demanding new robust NIR fluorophores. Such photo- and chemo-stable NIR fluorophores, linear-shape π-extended rosol and rosamine analogues, are disclosed here which provide bright fluorescence images in cells as well as in tissues by confocal laser-scanning microscopy. Furthermore, they offer unique ratiometric imaging platforms for activatable probes with dual excitation and dual emission capability, as demonstrated with a 2,4-dinitrophenyl ether derivative of benzo-rosol.

## Abstract 2

NIR-emitting benzo-rosol and -rosamine dyes offer novel ratiometric imaging platforms with high pohoto- and chemo-stability.

## Graphical abstract

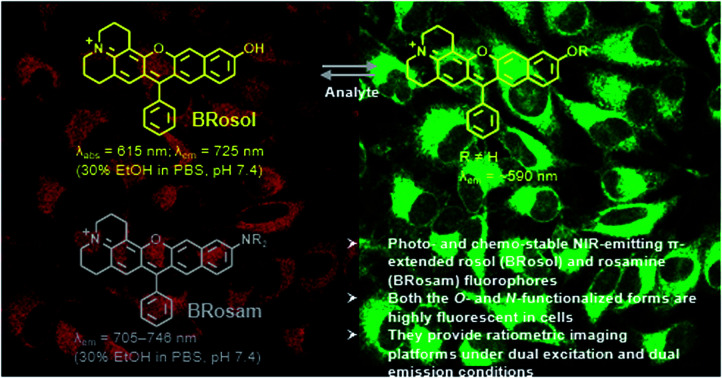

## Introduction

Microscopic imaging aided with a fluorescent probe is essential for studying biological processes in living systems. Accordingly, fluorescent organic molecules (fluorophores) and probes have been evolved for bioimaging applications. Fluorophores that emit in the deep-red/near-infrared (NIR) wavelength region have received particular attention for deep-tissue or whole body imaging applications,^1,2^ because with such dyes we can minimize the autofluorescence interference from innate biomolecules and thus increase the imaging depth. Cyanines (Cy) and analogues constitute a representative class of deep-red/NIR dyes. For example, ICG, an FDA-approved Cy7 dye, has been importantly used for tumour imaging and photodynamic therapy. A key structural feature of Cy dyes is that two identical nitrogen-containing heterocycles, one with a positive charge, are resonance-stabilized through “flexible” polymethine units. Cy dyes have limited photo-stability,^3^ however, thwarting their use for long-term bioimaging applications. In addition, Cy dyes and their asymmetric analogues hemicyanines seem to have limited chemical stability (chemo-stability) toward some reactive biological species, such as hypochlorous acid, hydrogen sulfide or bisulfite, as inferred from the various cyanine- and hemicyanine-based activatable probes for these species (Table S1, ESI†). Therefore, new NIR-emitting dyes that are photo- and chemo-stable are in great demand.

**Scheme 1:**
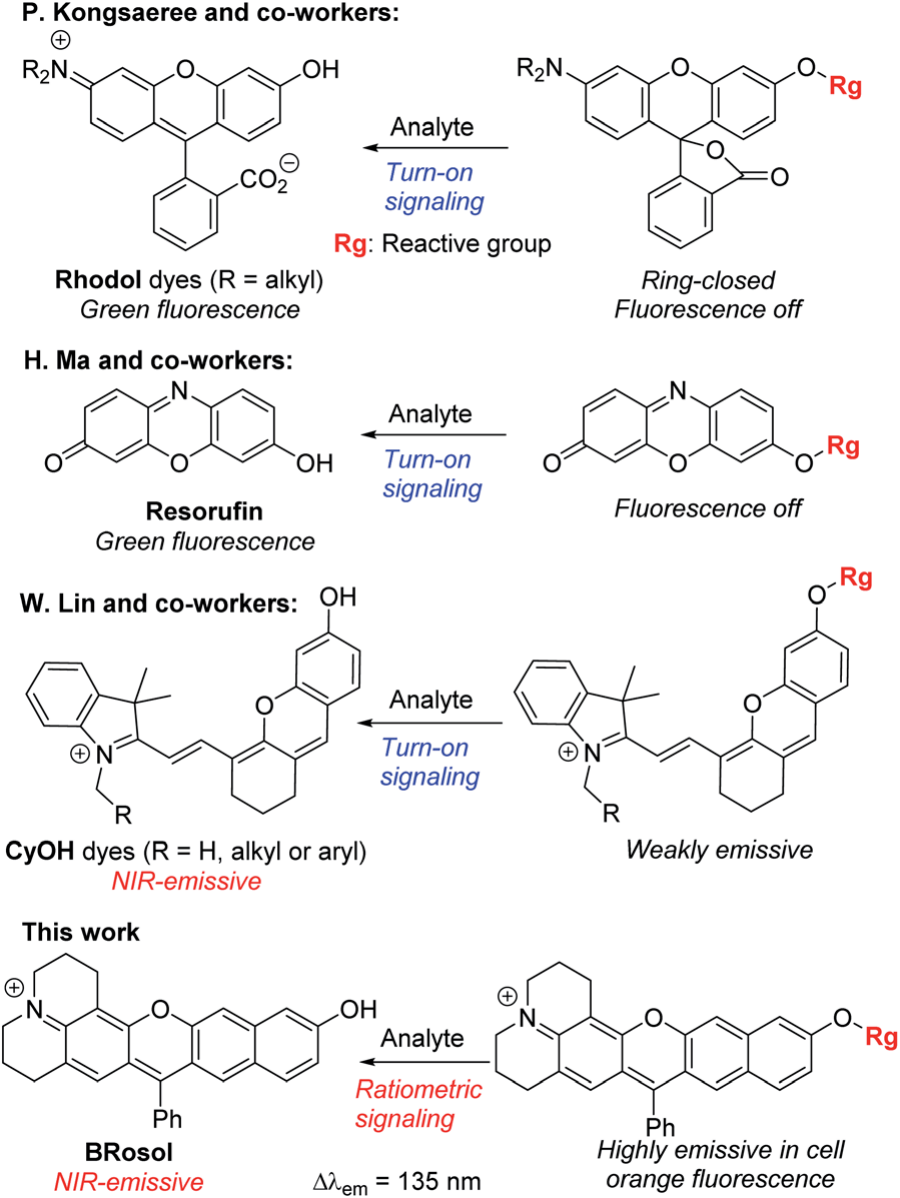
Sensing properties of ArOH-type activatable probes.

Besides, for application in biomolecular tagging or detection of biological analytes, fluorophores with a functional arm are required. In particular, fluorophores with a functional arm through which we can modulate the emission properties are highly valuable.^4^ With such “armed” fluorophores we can develop the so-called reaction-based or activatable probes for biological analytes.^5,6^ For example, the rhodol system, a structural hybrid of rhodamine and fluorescein dyes, and resorufin offer an opportunity for the development of activatable probes; When the reactive group (Rg) attached onto the hydroxyl group of such a dye is selectively cleaved by a target analyte, it generates the parent dye with accompanying fluorescence changes and thus enables fluorescence detection (Scheme 1). This sensing scheme has been widely explored with other aryl alcohol (ArOH)-type fluorophores as well.^7–9^ Typical rhodols and also resorufin, however, absorb and emit in a rather shorter wavelength region, which, during microscopic imaging of tissues, can cause significant autofluorescence from innate biomolecules such as flavoproteins. To minimize the autofluorescence interference, which mostly ranges from the blue to orange wavelength region, fluorophores that emit in longer wavelengths (λ_em_ ≥ 630 nm) are in great demand.^2^ Recently, Lin and co-workers developed a new class of ArOH-type fluorophores, so-called **CyOH** dyes,^9^ which is a hybrid system of cyanine and fluorescein dyes. The **CyOH** dyes emit in the NIR region, alleviating autofluorescence and also the shallow imaging depth in tissue imaging. Accordingly, they are increasingly used to develop fluorescent probes for various analytes, such as tyrosinase,^10^ esterases,^11^ β-glucuronidase,^12^ keloid,^13^ phosphatase,^14^ peptidases,^15^ and cytochrome P450,^16^ by introducing the corresponding reactive group to the hydroxyl arm.

Although **CyOH** dyes have promising features such that they emit in the NIR wavelength region and have an hydroxyl functional arm through which their emission properties can be modulated, we have found that they have limited photostability (see below) as well as limited chemostability.^17^

Besides, the **CyOH** dyes in the *O*-functionalized form (ArORg) are weakly emissive, as in the case of rhodol and resorufin dyes: accordingly, the analyte-triggered conversion from ArORg to ArOH results in a turn-on type fluorescence response (Scheme 1). Such “single intensity-based” detection is widely used for bioimaging studies; however, it raises a serious reliability issue in quantitative analysis because the fluorescence intensity is inherently sensitive to environmental factors, such as polarity and viscosity changes. The reliability issue can be solved with ratiometric probes, which allow monitoring of fluorescence intensity ratio changes that are not so sensitive to the environmental fluctuations. Therefore, there is a strong need for new ArOH-type dyes whose *O*-functionalized forms also fluoresce but in different emission windows. Such dyes will find widespread use for the development of activatable probes with ratiometric imaging capability.

In this contribution, we wish to report the NIR-emitting dyes that show high photo- and chemo-stability, along with large Stokes shifts. The new dyes have π-extended features of rosol and rosamine dyes, which have hydroxyl and amino functional arms, respectively. Notably, their *O*- and *N*-functionalized derivatives also emit strong fluorescence with large peak separations from the parent dyes at the physiological pH, offering highly promising dye platforms for the development of ratiometric imaging probes. Surely, they provide bright fluorescence images in cells as well as in tissues by confocal laser-scanning microscopy (CLSM).

## Results and discussion

### Design and synthesis

**Fig. 1:**
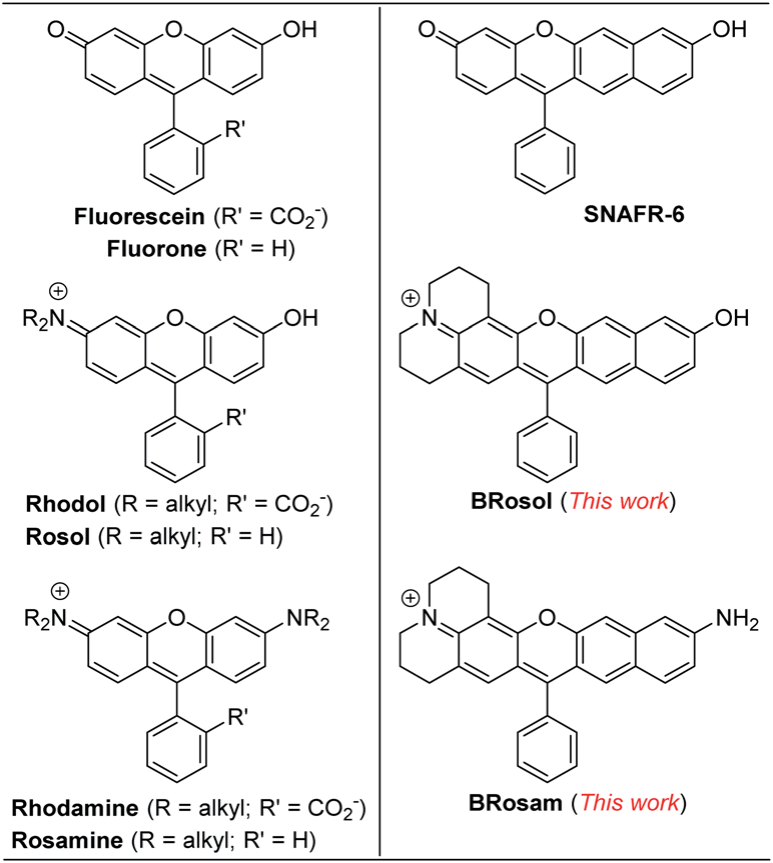
Structures of new and related dyes.

To develop NIR-emitting dyes that have high photo- and chemo-stability, we focused on linear-shape benzene-fused rosol (benzorosol: **BRosol**) analogues, from which the corresponding benzene-fused rosamine (benzorosamine: **BRosam**) derivatives can be also derived (Fig. 1). The new dyes belong to the push–pull type dipolar dyes having a hydroxyl or amino donor, which also acts as the functional arm. A linear-shape, benzene-fused fluorone such as **SNAFR-6** is reported by Strongin and co-workers,^18^ but linear-shape benzene-fused rosol or rosamine dyes have not been reported yet. Inspired by their studies, we have investigated the linear-shape benzene-fused rosol and rosamine systems. From our previous experience on benzene-fused coumarin^19^ and rhodamine dyes^20^ we expected that the linear-shape benzorosol and its analogues would emit in longer wavelengths, preferably in the NIR region, along with stronger fluorescence and larger Stokes shifts in comparison with the corresponding bent-shape isomers. Furthermore, we expected that the benzene-fused dye core which has no flexible vinyl units would bestow the desired photo- and chemo-stability in bioimaging applications; We suspect that the flexible vinyl unit(s) conjugated with the cationic heterocycle present in Cy and hemicyanine dyes is responsible for their limited photo- and chemo-stability.

**Scheme 2:**
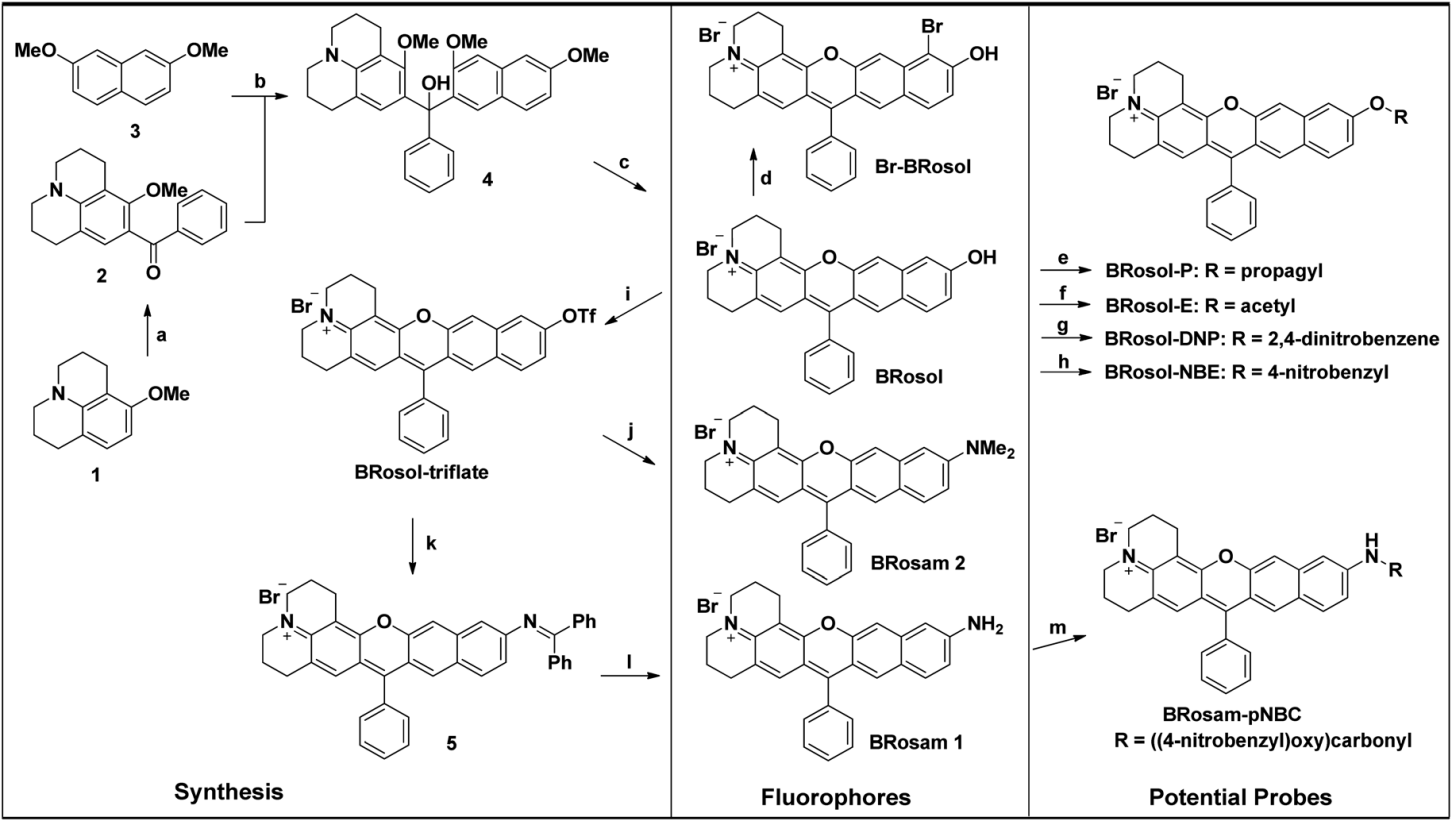
Synthesis of the linear-shape benzorosol (**BRosol**), benzorosamine (**BRosam**), and their derivatives. Reagents and conditions: (a) PhCOCl, AlCl_3_, CH_2_Cl_2_, 0 °C, 4 h, 30%; (b) (1) compound **2**, *n*-BuLi, TMEDA, THF, room temp., 2 h; (2) compound **3**, THF, −78 °C, 6 h, 50%; (c) BBr_3_, CH_2_Cl_2_, from −78 °C to room temp., 6 h, 95%; (d) NBS, CH_2_Cl_2_, room temp., 6 h, 54%; (e) propargyl bromide, Et_3_N, CH_3_CN, 50 °C, 24 h, 65%; (f) Ac_2_O, NEt_3_, room temp., 1 h, 83%; (g) 1-fluoro-2,4-dinitrobenzene, Et_3_N, CH_3_CN, room temp., 12 h, 75%; (h) 4-nitrobenzyl bromide, K_2_CO_3_, CH_3_CN, 60 °C, 12 h, 55%; (i) (CF_3_SO)_2_O, Et_3_N, CH_2_Cl_2_, room temp., 1 h, 79%; (j) Me_2_NH, Pd_2_(dba)_3_, Xantphos, Cs_2_CO_3_, toluene, 100 °C, overnight, 48%; (k) Ph_2_C=NH, Pd_2_(dba)_3_, Xantphos, Cs_2_CO_3_, toluene, 100 °C, overnight; (l) NaOAc, HONH_2_·HCl, MeOH, room temp., 1 h, 51%(2 steps); (m) 4-nitrobenzyl chloroformate, DMAP, pyridine, CH_2_Cl_2_, room temp., overnight, 86%.

The synthetic route to **BRosol** was established by adopting the synthetic procedure of **SNAFR**s (Scheme 2).^18^ Starting from 2,7-dimethoxynaphthalene (**3**) to 7-benzoyl-8-methoxyjulolidine (**2**), which, in turn, was prepared from 8-methoxyjulolidine (**1**) through the Friedel–Crafts acylation with benzoyl chloride, afforded the tertiary alcohol **4**. Treatment of compound **4** with boron tribromide at room temperature afforded **BRosol** through ring-closing followed by demethylation. Starting from commercially available chemicals, through three steps we were able to synthesize **BRosol**. At the tunable functional arm, the hydroxyl group of **BRosol**, we can readily introduce functional groups, such as propargyl (**BRosol-P**), acetyl (**BRosol-E**), and 2,4-dinitrophenyl (**BRosol-DNP**) through standard chemical conversions. **BRosol-P** will be useful for bioconjugation by click chemistry.^21^**BRosol-E** and **BRosol-DNP** could potentially be ratiometric probes for esterase and biothiols, respectively. In the last section, we demonstrate that **BRosol-DNP** indeed can be used to sense glutathione (GSH), a major biothiol, with a ratiometric fluorescence response under the dual excitation and emission conditions.^22,23^**BRosol** can be also functionalized to provide *o*-bromo derivative, **Br-BRosol**, which would allow further derivatization through transition metal-catalyzed coupling reactions.

**BRosol** can further provide the corresponding amine analogues, **BRosam** dyes; Through the Buchwald–Hartwig reaction of the **BRosol-triflate** with dimethylamine or benzophenone imine, the corresponding benzorosamine **BRosam 2** and the imine intermediate **5** were readily prepared, respectively. Hydrolysis of the diphenylimino group of compound **5** afforded the corresponding benzorosamine with a simple amino substituent, **BRosam 1**. The free amino group of **BRosam 1** provides a tunable arm for further functionalization. For example, we can introduce a *p*-nitrobenzyloxycarbonyl (*p*-NBC) group to it, affording the *p*-nitrobenzylcarbamate derivative **BRosam-pNBC**. All the new compounds were fully characterized by ^1^H NMR, ^13^C NMR, and HRMS analyses (ESI†).

### Photophysical properties of BRosol and its derivatives

The absorption and emission spectra of **BRosol** and its derivatives (**BRosol-E** and **BRosol-P**) were compared in organic and also in aqueous media. The compounds have higher absorbance and fluorescence intensity in organic solvent such as dichloromethane, acetonitrile and ethanol (Fig. S1 in the ESI,†Table 1), but lower absorbance and fluorescence in PBS (10 mM, pH 7.4). Notably, inspite of the low fluorescence in the buffer only, **BRosol** and its derivatives provide bright cellular fluorescence images under normal imaging conditions (at 10 μM fluorophore with 5% laser power; see below). The unique cellular environment, which is more viscous and hydrophobic compared with that of aqueous, and the dipolar nature of the fluorophores are likely to be responsible for the contrasting emission behaviour. **BRosol** and its derivatives including those of **BRosam** belong to dipolar dyes, whose emission behaviour is highly dependent on media. In general, dipolar dyes are weakly or poorly emissive in aqueous media but become highly fluorescent in a less polar and more viscous environment.^24^ Accordingly, dipolar dyes display pronounced changes in their emission intensity compared to that of the “resonance-symmetric” dyes such as rhodamine, fluorescein, and cyanine dyes when going from aqueous media to the cellular medium.

**Table 1:** Photophysical properties of **BRosol** and its derivatives

Medium	**BRosol**				
	*λ*_abs_ (nm)	*λ*_em_ (nm)	Stoke shift (nm)	*ε*	*Φ*_f_ a
PBS pH 7.4	516	586	70	18 300	0.021
CH_2_Cl_2_	525	611	86	35 900	0.276
CH_3_CN	515	589	74	37 600	0.192
Ethanol	520	595	75	39 900	0.103
0.3 EtOH/PBS (pH 7.4)	515	590	75	21 600	0.037
	615	725	110	4800	0.020

**BRosol-E**					
PBS pH 7.4	453	601	148	19 500	0.088
CH_2_Cl_2_	478	591	113	24 000	0.316
CH_3_CN	512	595	83	24 600	0.202
Ethanol	471	595	124	23 900	0.088

**BRosol-P**					
PBS pH 7.4	476	591	115	24 400	0.064
CH_2_Cl_2_	517	585	68	30 400	0.273
CH_3_CN	466	590	124	28 800	0.183
Ethanol	514	589	75	30 400	0.108

a The fluorescence quantum yields determined using Nile blue (*Φ*_F_ = 0.27 in EtOH) as a reference fluorophore. The concentration of **BRosol** was 10 μM in the organic solvent (containing 1% DMSO) or 1.0 μM in PBS (containing 0.1% DMSO), the concentration at which the dye shows no aggregation-caused quenching.

**Fig. 2:**
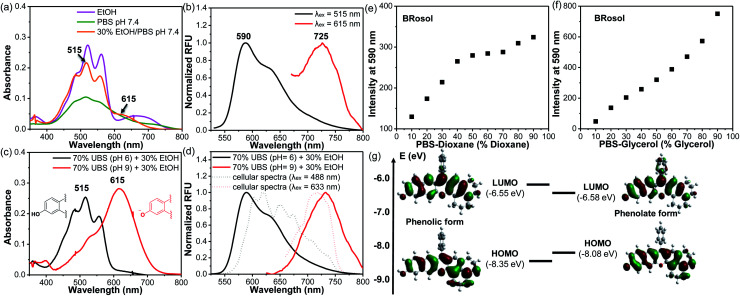
(a) Absorption spectra of **BRosol** (10 μM) obtained in different solvents (EtOH, pH 7.4 PBS, and 30% EtOH/PBS (pH 7.4); all containing 1% DMSO). (b) Emission spectra of **BRosol** (10 μM) in 30% EtOH/PBS (pH 7.4), obtained under dual excitation at 515 nm and 615 nm, respectively. (c) Absorption spectra and (d) normalized emission spectra of **BRosol** (10 μM) in 70% UBS (pH 6; pH 9) + 30% EtOH. The emission spectra were obtained under excitation at the absorption maximum in the given medium. Plots of the fluorescence intensity changes of **BRosol** (10 μM) in (e) pH 7.4 PBS-dioxane and in (f) pH 7.4 PBS-glycerol mixtures: the fluorescence intensity was estimated as the emission peak height at 590 nm under excitation at 515 nm. (g) Electron distribution and energy state of the HOMO and LUMO for the phenolate and phenolic forms of **BRosol**, respectively, calculated using Gaussian’09 with the hybrid B3LYP functional and 6-31 G(d,p) basis set.

The photophysical properties of **BRosol** were measured in a mixed solvent system of 30% ethanol in PBS (pH 7.4). In the mixed medium, in addition to the absorption band of the phenolic form of **BRosol** (λ_abs_ = 515 nm), that of the phenolate form gave substantial fluorescence (λ_abs_ = 615 nm) (Fig. 2a). When being excited at each of the two absorption maxima, emissions from the orange (λ_em_ = 590 nm) and NIR (λ_em_ = 725 nm) regions were observed, respectively (Fig. 2b).

The hydroxyl group of **BRosol** has p*K*_a_ = 7.4, which is less acidic than that of the **CyOH** fluorophore (p*K*_a_ ∼5.6). Thus, **BRosol** exists both in the phenolic and phenolate forms in an approximately 1 : 1 ratio in the cytosol (Fig. S2 in the ESI†). Thus, **BRosol** would enable cellular fluorescence imaging in the NIR region. Based on the p*K*_a_ value, we obtained the absorption and emission spectra of the phenolic and phenolate forms of **BRosol** in the mixed solvent system of 30% EtOH + 70% UBS (Universal Buffer System, at pH 6.0 and pH 9.0 respectively) (Fig. 2c and d). At pH 6.0, **BRosol** mostly exists in its phenolic form, having λ_abs_ = 515 nm and λ_em_ = 590 nm. However, at pH 9, **BRosol** mainly exists in its phenolate form, displaying the absorption and emission maxima at λ_abs_ = 615 nm and λ_em_ = 725 nm, respectively. The normalized emission spectra of the phenolic and phenolate forms of **BRosol** (Fig. 2d) are matched well with those obtained at pH 7.4 (Fig. 2b). The polarity- and viscosity-dependent emission behaviour of **BRosol** shown in Fig. 2e and f, respectively, indicates that it becomes strongly fluorescent in a less polar and more viscous environment. The environment-sensitive emission behaviour supports that **BRosol** and its derivatives are dipolar dyes. The quantum yield of **BRosol** in organic solvent is quite high (*Φ*_f_ = 28% in CH_2_Cl_2_) but reduces in PBS (*Φ*_f_ = 2.1%). In PBS, **BRosol** and its derivatives seem to exhibit aggregation-induced quenching (ACQ) even in the linear absorbance–concentration range (Fig. S3†), hence we measured the quantum yields of the dyes in PBS at a low concentration (1.0 μM) to avoid the ACQ effect. ACQ is a common issue for hydrophobic dyes. To inhibit ACQ in the case of **Brosol**, it would be necessary to introduce a steric group in the benzene ring in **BRosol**. The photophysical data of **BRosol** and its derivatives are listed in Table 1.

It is notable that the absorbance from the phenolic form of **BRosol** becomes insignificant above 600 nm, whereas that of the phenolate form displays the maximum at 615 nm (Fig. 2c). Therefore, we can selectively excite the phenolate form over the phenolic form by irradiation at 615 nm or higher. This is an important feature for the ratiometric imaging under dual excitation conditions. Suppose a ratiometric probe generated from **BRosol**, labeled **ArORg**, is converted to **ArOH** through activation by an analyte, we can monitor the formation of the enzymatic product separately from the probe by following the phenolate form.

The calculated HOMO–LUMO energy gap of the phenolate form (ketone) was 0.3 eV smaller than that of the phenolic form of **BRosol** (Fig. 2g), which corroborates the red-shifted absorption and emission bands of the phenolate form. Both forms have highly conjugated electronic distributions.

At this stage, we have briefly assessed the absorption and emission behaviour of **Br-BRosol**; its absorption spectra in different solvents indicated that the phenolate form becomes major in ethanol, which is likely due to the more acidic hydroxyl group (p*K*_a_ = 5.8) (Fig. S4 and S5 in the ESI†). Accordingly, the NIR emission from the phenolate form is much stronger than the red emission from the phenolic form (Table S2†). A further study to derivatize **Br-BRosol** and apply it to develop ratiometric probes is the next concern.

### Photophysical properties of **BRosam** and its derivatives

**Fig. 3:**
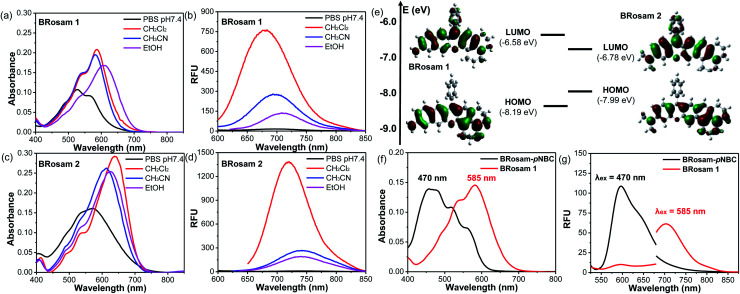
(a–d) Absorbance and emission spectra of **BRosam 1** and **BRosam 2**, each at 10 μM (containing 1% DMSO) in different media (PBS, CH_2_Cl_2_, CH_3_CN and EtOH), obtained under excitation at the maximum absorption wavelength. The HeLa cells were stained with each fluorophore at 10 μM for 30 min and then fixed with 4% formaldehyde. (e) Frontier molecular orbitals of **BRosam 1** and **BRosam 2**, calculated using Gaussian’09 built-in hybrid density functional B3LYP with the 6-31 G(d,p) basis set. (f) Absorption and (g) emission spectra of **BRosam 1** and **BRosam-pNBC** (each at 10 μM in 30% EtOH/PBS containing 1% DMSO). The emission spectra were obtained under dual excitation at (λ_ex_ = 470 nm) (slit: 4 nm/4 nm) and 585 nm (slit: 5 nm/5 nm), respectively.

Starting from **BRosol**, we can readily synthesize the corresponding benzo-rosamine analogues, **BRosam**. The **BRosam** compounds lack the carboxy group that forms the spirolactone equilibrium species and thus behave differently from the corresponding rhodamine fluorophores. The **BRosam** dyes **1** and **2** emit in the NIR region in ethanol, at 708 nm and 740 nm respectively. Thus, they are also of interest as a new class of NIR-emitting fluorophores. We briefly evaluated their photophysical properties. The **BRosam** dyes emitted fluorescence insensitive to pH changes in the range from pH 4–12, because the aryl amines are poorly basic (Fig. S6 in the ESI†). This is a distinctive feature from the ArOH-type fluorophores that have a more acidic hydroxyl group. The **BRosam** dyes are also strongly emissive in dichloromethane but weakly emissive in PBS (pH 7.4) (Fig. 3a–d), as in the case of **BRosol**. However, they also provided bright cellular images under excitation at low laser powers (see below). Furthermore, both **BRosam 1** and **BRosam 2** have large Stokes shifts of more than 170 nm (Table 2) in PBS (pH 7.4).

**Table 2:** Photophysical properties of **BRosam 1** and **BRosam 2**

Medium	**BRosam 1**				
	*λ*_abs_ (nm)	*λ*_em_ (nm)	Stoke shift (nm)	*ε*	*Φ*_f_ a
PBS pH 7.4	526	700	174	10 700	0.008
CH_2_Cl_2_	586	681	95	20 800	0.184
CH_3_CN	581	693	112	19 500	0.068
Ethanol	610	708	92	16 900	0.027

**BRosam 2**					
PBS pH 7.4	572	742	170	16 100	0.006
CH_2_Cl_2_	637	720	83	29 100	0.172
CH_3_CN	611	743	132	26 000	0.059
Ethanol	626	740	114	25 400	0.041

a Fluorescence quantum yields determined using Nile blue (*Φ*_F_ = 0.27 in EtOH) as a reference dye. The concentration of each dye was 10 μM in the organic solvent (containing 1% DMSO) or 1.0 μM in PBS (containing 0.1% DMSO).

Theoretical calculations (Fig. 3e) at the ground state indicated that, compared to **BRosam 1**, **BRosam 2** has the smaller HOMO–LUMO gap, and thus absorbs and emits the longer wavelengths in solution. However, their cellular spectra displayed emission peaks at similar wavelengths. Interestingly, their maximum emission wavelengths, *λ*_em_ = 707 nm and 710 nm respectively in the cellular environment, are not so shifted from that of **BRosol**. We can readily functionalize **BRosam** at the free amine site, for example, providing a carbamate derivative, **BRosam-pNBC** (Scheme 2). Such a *p*-nitrophenyl carbamate could be explored to develop a nitroreductase probe:^25^ a possible conversion of the carbamate to the corresponding amine by the enzyme would cause a dramatic color change from blue to red (Fig. 3f). The large absorption change is likely due to a change in the intramolecular charge-transfer (ICT) upon the conversion. Thus, **BRosam** also could offer a novel dye platform for the development of ratiometric fluorescent probes (Fig. 3g and S7, ESI†).

### Photo- and chemo-stability of the new fluorophores and their derivatives

**BRosol** is stable in solution (30% EtOH/PBS at pH 7.4): It maintained its emission intensity, more than 90%, at 37 °C when monitored at every hour for 24 h (Fig. S8 in the ESI†). It should be noted that cyanine dyes, such as **Cy7** and **Qcy7**, in PBS lose their emission intensity progressively.^26,27^

**Fig. 4:**
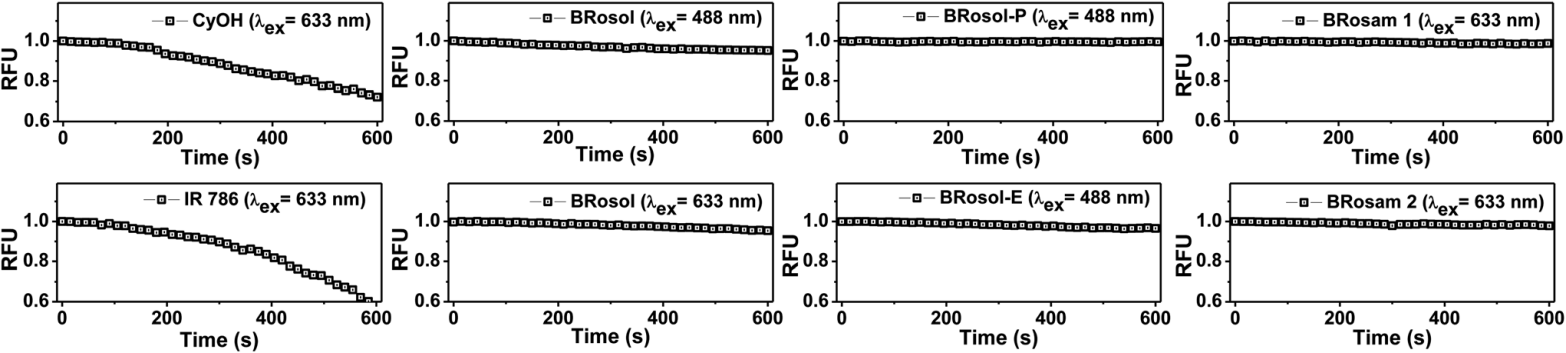
Time-dependent emission intensity changes of **CyOH**, **IR 786**, **BRosol**, **BRosam** and their derivatives (each at 10 μM in 30% EtOH/PBS (pH 7.4) containing 1% DMSO) upon continuous laser irradiation by CLSM, measured at 15 s intervals for 10 min. The laser power and excitation wavelength used: 0.05 mW and *λ*_ex_ = 488 nm for **BRosol**, **BRosol-E**, and **BRosol-P**; 0.18 mW and *λ*_ex_ = 633 nm for **CyOH**, **IR 786**, **BRosol**, **BRosam 1**, and **BRosam 2**.

As NIR-emitting dyes, currently cyanine and hemicyanine compounds or their analogues are widely used for bioimaging applications. However, owing to the flexible polymethine structure conjugated with the cationic heterocycle moiety, they have low photostability, thwarting their use in long-term monitoring of biological processes. When we monitored the fluorescence intensity of **CyOH** and **IR 786**, a hemicyanine and a **Cy7** dye respectively, in 30% EtOH/PBS (pH 7.4) by continuous laser (633 nm) irradiation for 10 min, they showed a substantial decrease in the emission intensity (Fig. 4). In contrast, **BRosol**, **BRosam 1**, and **BRosam 2** under the same laser irradiation conditions show slight changes in the emission intensity. **BRosol** and its derivatives **BRosol-P** and **BRosol-E** also maintain their emission intensity even under irradiation at 488 nm. Thus, the new fluorophores and their derivatives are highly photostable, showing minor changes in the emission intensity under the imaging conditions by confocal laser scanning microscopy.

As mentioned above, the cyanine and hemicyanine moieties are used to develop various activatable probes to detect hypochlorous acid,^28^ hydrogen sulfide^29^ or bisulfite,^30,31^ which, in turn, suggests that these fluorophores respond to the “reactive” biological species.^17^ Therefore, we have checked the stability of **BRosol** in the presence of the reactive chemical species each at 100 μM in 30% EtOH-PBS (100 mM; pH 7.4) at room temperature (∼25 °C) for 1 h. We used a rather high concentration of the buffer to eliminate any possible effect by pH change upon addition of the analytes. Under the given conditions **BRosol** is found to be stable toward the reactive species (Fig. S9 in the ESI†).

The high photo- and chemo-stability of the new fluorophores are likely due to their robust structural feature that consists of highly fused benzene rings instead of the flexible vinyl unit conjugated with the cationic heterocycle as in the case of cyanine and hemicyanine fluorophores. The cationic polymethine moiety seems to be responsible for the photo-degradation and also the sensitivity toward nucleophilic sulfur species.

### Comparison between **BRosol** and **CyOH**

To point out the advantageous features of **BRosol** as the ratiometric sensing platform, we have compared its basic photophysical properties with those of a **CyOH** fluorophore (Table 3 and S3†). The large Stokes shifts, the large emission peak separation, and the bright cellular fluorescence of **BRosol** as well as its *O*-functionalized derivatives (see below), in addition to their high photo- and chemo-stability (chemical stability), are highly desirable features for bioimaging applications.

**Table 3:** Comparison of the reaction-based sensing platforms, **CyOH** and **BRosol** fluorophores

	**CyOH** (R = Me)^a^ turn-on sensing platform		**BRosol** b ratiometric sensing platform	
	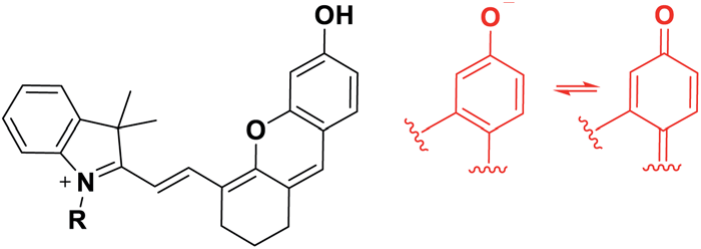		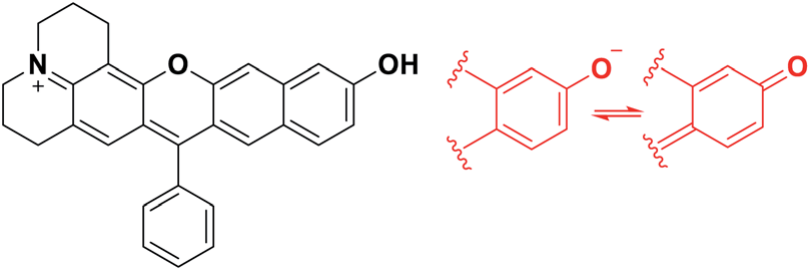	
	Phenolic (also, *O*-functionalized) form	Phenolate form	Phenolic (also, *O*-functionalized) form	Phenolate form
*λ*_abs_/*λ*_em_	654/677 nm	690/716 nm	515/590 nm	615/725 nm
Stokes shift	23 nm	26 nm	75 nm	110 nm
Δ*λ*_em_; p*K*_a_	39 nm; p*K*_a_ = 5.6		135 nm; p*K*_a_ = 7.4	
*Φ*_f_ in CH_2_Cl_2_	0.033		0.276	
Cellular brightness	Dark	Bright	Bright	Bright
Chemo-stability^c^	Response to bisulfite		Stable to bioanalytes	
Photo-stability^d^	Limited photo-stability		High photo-stability	

a Measured in PBS : MeOH = 1 : 1.

b Measured in PBS : EtOH = 7 : 3.

c See details in Fig. S11 (ESI).

d See details in Fig. 4.

At this point, it is also worthwhile to compare **BRosol** with **SNARF-1**, which has the *o*-carboxy substituent and thus can be classified as a π-extended rhodol analogue, benzorhodol (Fig. S10 in the ESI†). **SNARF-1** and its analogues are widely used for measuring of intracellular pHs.^32^ The common features of **SNARF-1**, in comparison to **BRosol**, are that it has a similar p*K*_a_ value of 7.4 and both the phenolic and phenolate forms are emissive. On the other hand, for **SNARF-1**, both the phenolic and phenolate forms have rather small Stokes shifts (31 nm and 48 nm, respectively), and the emission peak separation between the phenolic and phenolate forms is much smaller (Δ*λ*_em_ = 50 nm: *λ*_em_ = 586 nm at pH 6; *λ*_em_ = 636 nm at pH 9) than that of **BRosol**. Such bent-shape dipolar dyes generally emit in the shorter wavelengths and have smaller Stokes shifts compared with the corresponding linear-shape analogues.^19,20^

### Cellular imaging evaluation of **BRosol** and **BRosam** derivatives

Prior to cellular imaging, we have evaluated the biocompatibility of the new dyes. **BRosol** and its derivatives are biocompatible, as indicated by the cell viability assay conducted for each fluorophore at 5.0, 10, and 20 μM for 12 h. The **BRosam** dyes exhibited a little cellular toxicity at a higher concentration of 20 μM, but, at the 10 μM level, the cell viability was more than 90% even after 12 h (Fig. S11 in the ESI†).

**Fig. 5:**
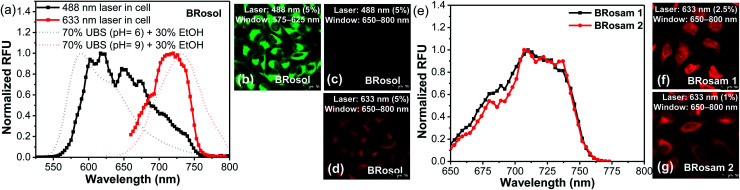
The cellular emission spectra and fluorescence images of HeLa cells incubated with **BRosol**, **BRosam 1** or **BRosam 2** (10 μM) for 30 min and then fixed with 4% formaldehyde, obtained by CLSM. (a) The emission spectra of the phenolic and phenolate forms of **BRosol** in the cells, obtained under dual excitation at 488 nm and 633 nm, respectively. (b–d) CLSM cellular images obtained by excitation with different laser sources (488 nm and 633 nm) and by collection of emissions from different windows (575–625 nm and 650–800 nm). (e) The emission spectra of **BRosam 1** and **2** in the cells, and (f, and g) cellular fluorescence images obtained under excitation at 633 nm and collection of emissions from 650–800 nm. The scale bar is 50 μm.

For the cellular imaging, first we obtained the cellular emission spectra of **BRosol** under excitation at different wavelengths (laser sources: 488 nm, 514 nm, 561 nm, 594 nm and 633 nm) at 1–5% laser power (Fig. 5 and S12 in the ESI†). Under excitation at 488 nm, at which the phenolic form of **BRosol** has a significantly higher absorbance than its phenolate form, the cellular emission spectrum overlaps well with that of the phenolic form observed in solution at pH 6.0. Under excitation at both 514 nm and 561 nm, the cellular emission spectra cover both the phenolic and phenolate forms. Under excitation at 594 nm and further at 663 nm where the phenolic form has insignificant absorbance, the cellular spectra overlap well with the emission spectrum of the phenolate form obtained in the pH 9.0 solution. According to the cellular emission spectra obtained under excitation at 488 nm and 633 nm, respectively, during cellular imaging by confocal laser-scanning microscopy (CLSM), we can resolve the emissions from the phenolic and phenolate forms of **BRosol** through separate windows, 575–625 nm and 650–800 nm respectively (Fig. 5b–d). Thus, fluorescent probes based on **BRosol** are expected to enable ratiometric cellular imaging with a minimal spectral overlap under the dual excitation/emission conditions. Indeed, this is the case, as demonstrated with a **BRosol**-derived biothiol probe (see below). In the case of **BRosam** dyes, their cellular emission spectra are very similar: They are broad and cover a wide spectral range from 650–750 nm (Fig. 5e). **BRosam** dyes also provide very bright fluorescence images in cells at a lower laser power (1.0–2.5% at 633 nm, Fig. 5f and g), offering novel NIR-emitting dyes for bioimaging applications.

**Fig. 6:**
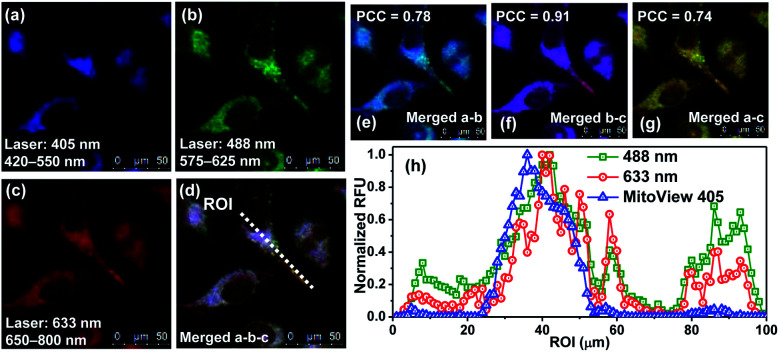
CLSM images of HeLa cells co-incubated with **BRosol** (5.0 μM) and **MitoView 405** (0.5 μM) (stains mitochondria) in PBS (10 mM, pH 7.4) for 15 min at 37 °C. (a) *λ*_ex_ = 405 nm; *λ*_em_ = 420–550 nm (**MitoView 405**). (b) *λ*_ex_ = 488 nm and *λ*_em_ = 575–625 nm (the phenolic form of **BRosol**) (c) *λ*_ex_ = 633 nm; *λ*_em_ = 650–800 nm (the phenolate form of **BRosol**). (d) Merged image of (a), (b), and (c). (e) Merged image of (a) and (b). (f) Merged image of (b) and (c). (g) Merged image of (a) and (c). (h) Intensity profile along the ROI (region-of-interest) in the image (d). PCC: Pearson's correlation coefficient. The scale bar is 50 μm.

At this point, we conducted dye colocalization experiments to figure out the cellular localization of **BRosol** (Fig. 6), with respect to its phenolic and phenolate forms. Because **BRosol** contains a hydrophobic and cationic moiety, we suspected whether it, either in the phenolic or phenolate form, could localize in the mitochondria. To this end, we chose a commercial blue dye **MitoView 405** that selectively stains the mitochondria. HeLa cells coincubated with **BRosol** and **MitoView 405** for 15 min at 37 °C were fluorescently imaged by confocal laser scanning microscopy (CLSM). By choosing appropriate excitation wavelengths (405 nm, 488 nm, and 633 nm) and emission windows (blue, green, and red), it was possible to separate fluorescence images from **MitoView 405**, the phenolic **BRosol**, and the **BRosol** phenolate. The resulting colocalization data indicate that **BRosol**, either in the phenolic or phenolate form, mostly localizes in the mitochondria (Fig. 6): The intensity profiles along the region-of-interest (ROI) show a small discrepancy region and the Pearson's colocalization coefficient (PCC) values are quite high (PCC = 0.78 between the images of **MitoView40** and phenolic **BRosol**; PCC = 0.74 between the images of **MitoView40** and **BRosol** phenolate). Both the phenolic and phenolate forms of **BRosol** have a higher PCC value of 0.91.

### Imaging of tissues with **BRosol** and **BRosam** derivatives

**Fig. 7:**
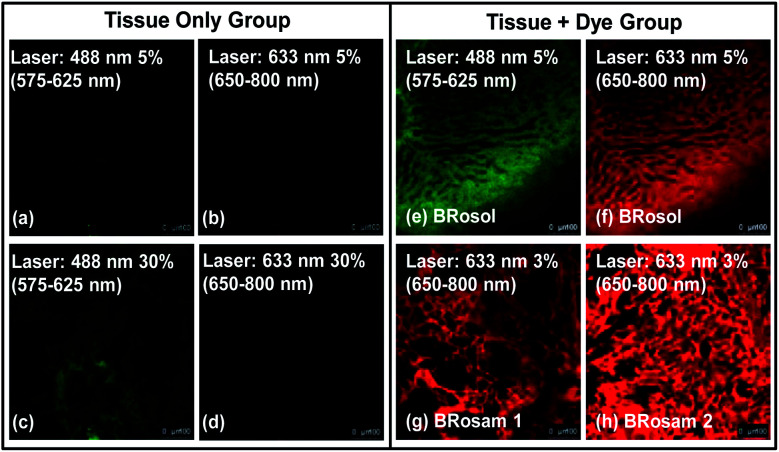
Fluorescence images of mouse liver tissues incubated with **BRosol**, **BRosam 1** or **BRosam 2**, each at 10 μM (containing 1% DMSO) for 1 h, obtained by CLSM. (a–d) Images of the tissue only, obtained under excitation at 488 nm and 633 nm with 5% and 30% laser power, respectively, which show autofluorescence. (e) Image from the phenolic **BRosol**, obtained under excitation at 488 nm (5% laser power) and collection of emissions from 575–625 nm. (f) Image from the **BRosol** phenolate, obtained under excitation at 633 nm (5% laser power) and collection of emissions from 650–800 nm. Images from (g) **BRosam 1** and (h) **BRosam** 2, both obtained under excitation at 633 nm (3% laser power) and collection of emissions from 650–800 nm. The scale bar is 100 μm.

A big advantage of deep-red/NIR emitting dyes is that they enable florescence imaging with minimal autofluorescence, fluorescence interference from innate biomolecules. All the dyes provide bright fluorescence images from the green-orange and deep-red emission channels, respectively (Fig. 7). Accordingly, at the low laser power (≤5%), by using the new dyes we can obtain bright fluorescence images of tissues with little autofluorescence interference.

### Application of BRosol as a ratiometric imaging platform

To demonstrate the potential of **BRosol** as a ratiometric imaging platform, **BRosol-DNP** (DNP represents the 2,4-dinitrophenyl group) was studied as a new biothiol probe, as other aryl DNP ethers are known to sense biothiols such as glutathione (GSH).^33–35^ For example, GSH displaces the DNP moiety in related aryl DNP ethers (**ArO-DNP**) through nucleophilic aromatic substitution, generating the corresponding aryl alcohols (**ArOH**). This conversion was mostly accompanied by a fluorescence turn-on response, owing to the fluorescence quenching effect by the nitro group through the photo-induced electron transfer (PET) mechanism. However, unlike the common cases, **BRosol-DNP** emitted strong fluorescence in a wide emission window from the green to red regions when excited at its absorption maximum, 460 nm (Fig. S13 in the ESI†). As the absorption and emission spectra of **BRosol** itself appear at different wavelengths from the probe, it can be used to detect GSH with ratiometric signal responses (see below).

**Fig. 8:**
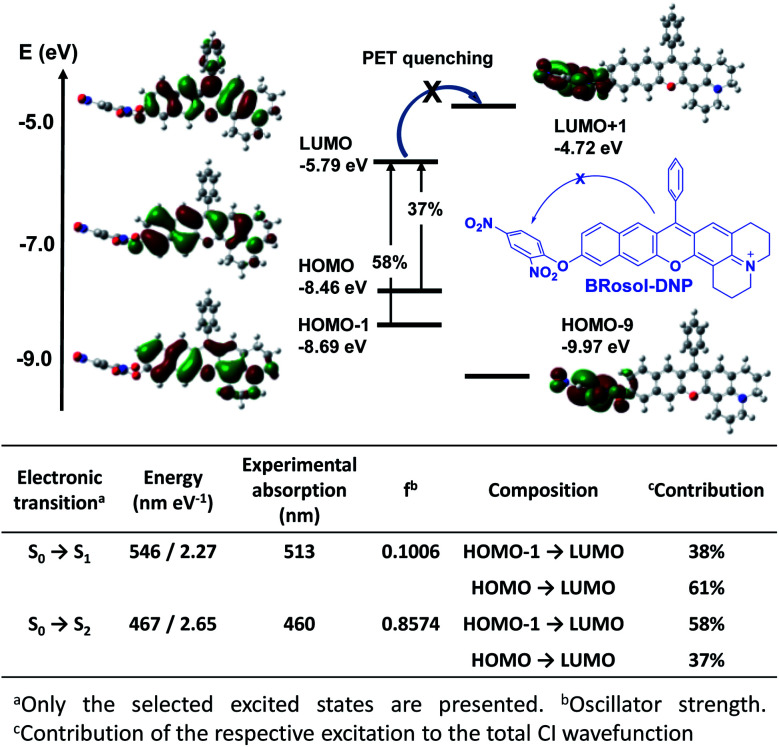
Frontier molecular orbitals of **BRosol-DNP** obtained through TD-DFT calculations using Gaussion’09 built-in hybrid density functional B3LYP with the 6-31 G(d,p) basis set. The percentage values indicate contribution of the respective excitation to the total CI wavefunction.

The “non-quenched” emission behaviour from the DNP-substituted **BRosol** is likely due to the lower HOMO energy level of the **BRosol** moiety compared to the LUMO level of the DNP, which makes the possible PET quenching difficult. Thanks to the highly conjugated nature of **BRosol** and thus the lower LUMO energy level (Fig. 8), the commonly observed PET-quenching from the nitroaryl group is suppressed in both the dinitrophenyl- and mononitrophenyl-substituted **BRosol** derivatives (**BRosol-NBE**: Fig. S14 and S15 in the ESI†). Significant orbital overlaps between the LUMO and HOMO (also HOMO−1) also support the highly fluorescent nature of the dinitrophenyl derivatives. In other words, the highly conjugated feature of **BRosol** and thus its lower LUMO energy level is an added merit, from which we can generate the fluorescent nitroaryl derivatives.

**Fig. 9:**
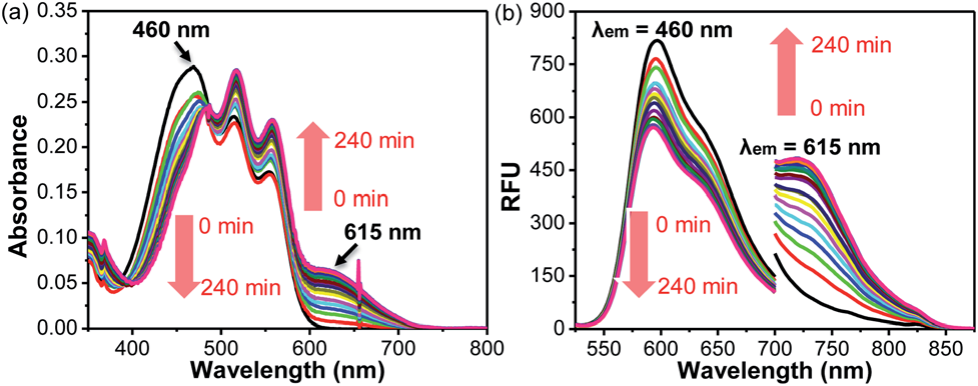
Time-dependent (a) absorption and (b) emission spectral changes of **BRosol-DNP** (10 μM, containing 1% DMSO) in the presence of GSH (10 mM) in 30% EtOH/PBS (100 mM, pH 7.4). The emission spectra were obtained under dual excitation at 460 nm (slit: 4 nm/4 nm) and 615 nm (slit: 12 nm/12 nm), respectively.

Given that **BRosol** and **BRosol-DNP** emit fluorescence in different channels, we moved to evaluate the ratiometric imaging capability of **BRosol-DNP** toward intracellular GSH under the dual excitation/emission conditions. First, we evaluated the ratiometric sensing behavior of **BRosol-DNP** toward GSH in the mixed medium of 30% EtOH/PBS (pH 7.4) at 37 °C. In the presence of GSH at a biologically relevant concentration (10 mM), the emission peak from **BRosol-DNP** (10 μM) at 590 nm decreased gradually while a new peak at 725 nm increased, which was ascribed to the **BRosol** molecules produced from the supposed nucleophilic aromatic substitution by GSH. Concurrently, the absorption peak at 460 nm from the probe decreased while that at 615 nm (from the **BRosol** produced) increased (Fig. 9a and b). There were no further signal changes after 180 min under the conditions (Fig. S16 in the ESI†).

**BRosol-DNP** also showed high selectivity toward GSH over other potentially competing analytes; It exhibited prominent absorption and emission spectral changes only toward GSH over the other analytes, when examined for the biothiols at their biologically relevant concentrations and for the other analytes at 1.0 mM level. Only hydrogen sulfide and cysteine may cause slight interference in detecting intracellular GSH levels, as shown in the selectivity data presented by the ratio values in the absorbance (*A*_615_/*A*_460_) or in the fluorescence intensity (*I*_725_/*I*_590_) estimated by the peak height (Fig. S17 in the ESI†). The basal ratio (*I*_725_/*I*_590_) values are due to the residual fluorescence of the probe in the NIR region. The time-course fluorescence response of **BRosol-DNP** toward GSH at different concentrations exhibits concentration-dependent changes (Fig. S18 in the ESI†).

**Fig. 10:**
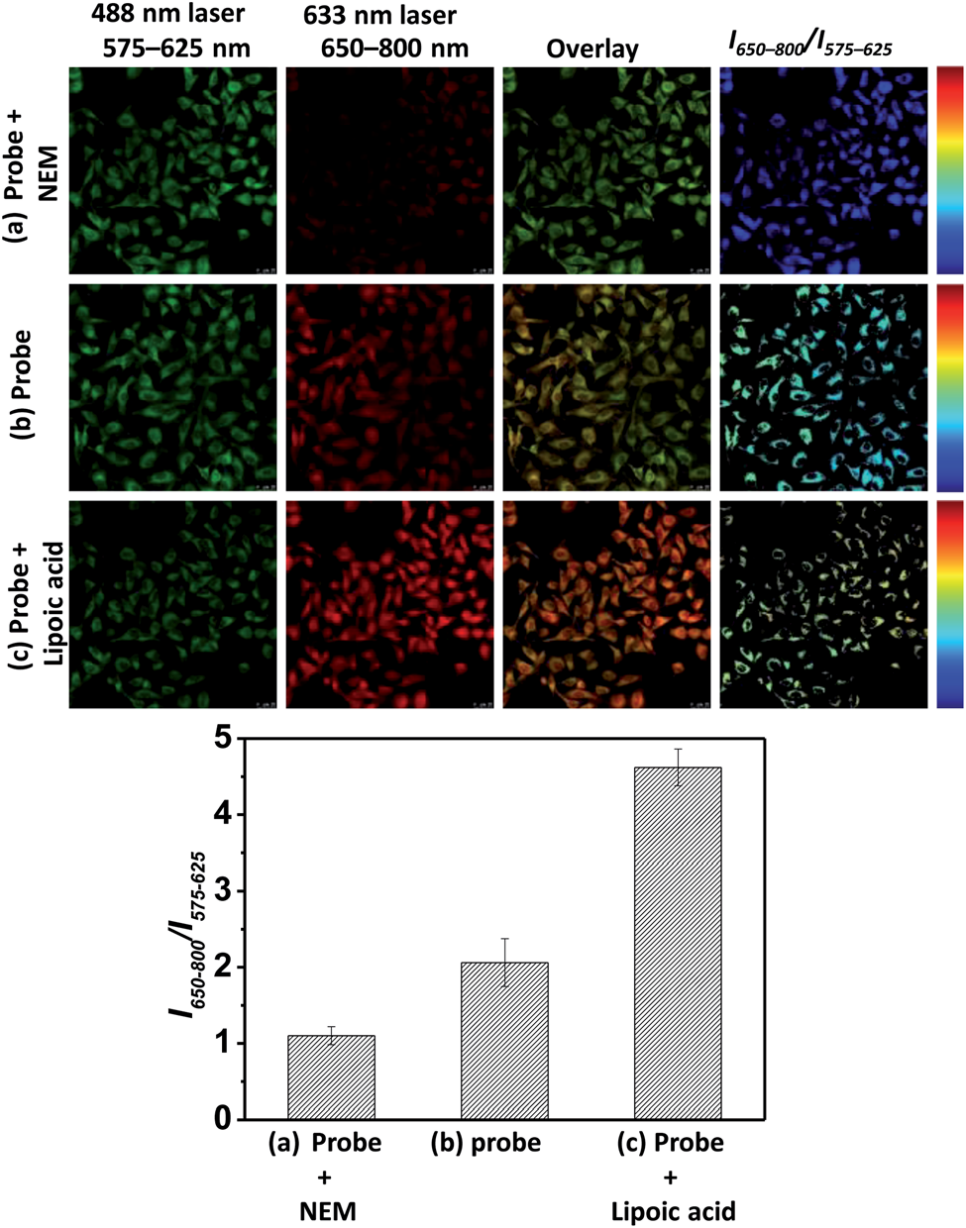
Fluorescence images of HeLa cells incubated with **BRosol-DNP** (10 μM) in PBS (10 mM, pH 7.4) at 37 °C for 120 min, obtained by CLSM under dual excitation and emission conditions (*λ*_ex_ = 488 nm at 5% laser power, *λ*_em_ = 575–625 nm; *λ*_ex_ = 633 nm at 30% laser power, *λ*_em_ = 650–800 nm): HeLa cells (a) pre-incubated with NEM (100 μM) for 30 min, (b) incubated only with the probe, and (c) pre-incubated with lipoic acid (500 μM) for 24 h. The scale bar is 20 μm.

In light of the ratiometric sensing capability of **BRosol-DNP** toward GSH in solution, next we applied it to observe intracellular GSH levels through ratiometric fluorescence imaging under the dual excitation/emission conditions. HeLa cells incubated with **BRosol-DNP** (10 μM) at 37 °C for 2 h were subjected to fluorescence imaging using confocal laser scanning microscopy. As a negative control experiment, prior to incubation with the probe, HeLa cells were pre-incubated for 30 min with *N*-ethylmaleimide (NEM, 100 μM), which consumes GSH to form the corresponding conjugate addition product (Fig. 10a). As a positive control, the time HeLa cells were pre-incubated for one day with lipoic acid (500 μM), which increases the intracellular GSH level (Fig. 10c).^36^ The cellular fluorescence images indicate a lower level of intracellular GSH in the negative control, whereas an opposite result in the case of the positive control experiment. A comparison of the signal intensity ratios between the red and green images (*I*_650–800_/*I*_575–625_) provides the relative GSH levels quantitatively. At this point, it should be noted that fluorescence of such phenolic probes would be dependent on pH changes in general. Therefore, we should take care of such interference in real applications. In this regard, the use of **Br-BRosol** that has a lower p*K*_a_ value would be better than the use of **BRosol** for the probe development.

In short, **BRosol** offers a unique ratiometric sensing platform capable of fluorescence microscopic imaging under the dual excitation/emission conditions, as demonstrated here with **BRosol-DNP**. Thus, a door is open to develop various fluorescent probes with ratiometric imaging capability under the dual excitation/emission conditions, by introducing other analyte-specific reactive groups to **BRosol** and its analogues at the hydroxyl arm.

## Conclusions

NIR-emitting compounds such as cyanine and hemicyanine dyes are widely used for bioimaging. Their structures are characterized by a polymethine moiety conjugated to a cationic heterocycle, which seems to be the feature responsible for their limited photostability. The key structural component is also known to react with some reactive oxygen species and biothiols, as indicated by a number of so-called reaction-based probes. Therefore, there is a strong demand to develop new NIR fluorophores that have high photostability as well as high chemo-stability toward those reactive species. Such NIR fluorophores are disclosed here, which have a functional arm and, furthermore, provide novel ratiometric imaging platforms. The new fluorophore system, benzorosol, belongs to aryl alcohol-type NIR fluorophores, of which *O*-functionalized derivatives also provide bright cellular fluorescence in contrast to indolinum-containing hemicyanine dyes. Both the free hydroxyl and *O*-functionalized forms have large Stokes shifts as well as a large emission peak separation, enabling ratiometric bioimaging under the dual excitation and dual emission conditions. From benzorosol, the corresponding benzorosamine compounds can be readily prepared, which possess similar photophysical properties to the parent benzorosol. The ratiometric imaging capability of benzorosol is demonstrated by its 2,4-dinitrophenyl ether derivative, which selectively detects cellular glutathione. The new fluorophore systems have several notable features, such as ease of synthesis, NIR emission, high photo- and chemo-stability, large Stokes shifts, biocompatibility, and high cellular brightness. As robust NIR fluorophores, they hold great promise for bioconjugation as well as for the development of ratiometric imaging probes.

## Conflicts of interest

There are no conflicts to declare.

## Supplementary Material

SC-011-D0SC03314F-s001
