# Supplementary material for: Development of photo- and chemo-stable near-infrared-emitting dyes: linear-shape benzo-rosol and its derivatives as unique ratiometric bioimaging platforms
Source: Chem Sci. 2020 Aug 6;11(33):8901–11. doi: 10.1039/d0sc03314f (PMC8163444; doi:10.1039/d0sc03314f)

Electronic Supplementary Material (ESI) for Chemical Science.
This journal is © The Royal Society of Chemistry 2020

Development of Photo- and Chemo-stable Near-Infrared-Emitting Dyes: Linear-Shape Benzo-Rosol and Its Derivatives as Unique Ratiometric Bioimaging Platforms

Mingchong Dai[a], Ye Jin Reo[a], Chang Wook Song[a], Yun Jae Yang[a] and Kyo Han Ahn* [a]

Department of Chemistry, Pohang University of Science and Technology (POSTECH), 77 Cheongam-Ro, Nam-Gu, Pohang, Gyungbuk 37673, Republic of Korea
E-mail: ahn@postech.ac.kr

1. General information……………………………………………………………………………….………………..2

2. Cell culturing and tissue experiments…………………………………………………………………………….2

3. Cytotoxicity assay……………………………………………………………………………...…….….…….2
.
4. Synthesis…………………………………………………………………………..……...…….……….2

5. Tables……………………………..……………………………………………………...……….….…7
.
6. Figures……………………………..……………………………………………………...…………….9

7. Spectra ………………………………………...………………………………………....…………………..…19

1. General information.

The chemical reagents were purchased from Aldrich, Alfa or TCI. Commercially available reagents were used without further purification. Anhydrous solvents for organic synthesis were prepared by passing through a solvent purification tower. All reactions were performed under argon atmosphere unless otherwise stated. Thin-layer chromatography was performed on precoated silica gel 60F-254 glass plates. ^{1}H and ^{13}C NMR spectra were measured with a Bruker AVANCE III 300 MHz, AVANCE III 500 MHz and AVANCE III 600 MHz FT-NMR spectrometer. Coupling constants (J value) are reported in Hertz. The chemical shifts (δ) are displayed in ppm, multiplicities are indicated by s (singlet), d (doublet), t (triplet), dd (doublet of doublets) and m (multiplet). Spectra are referenced to residual chloroform (7.26 ppm, ^{1}H, 77.16 ppm, ^{13}C). UV/Vis absorption spectra were obtained using a HP 8453 UV/Vis spectrophotometer. Fluorescence emission spectra were recorded on a Photon Technical International Fluorescence System using a 1 cm standard quartz cell. High-resolution mass spectra was recorded on a JEOL JMS-700 spectrometer at the Korea Basic Science Center, Kyungpook National University and the values are reported in units of mass to charge (m/z).

2. Cell culturing and tissue experiments

HeLa cells were maintained in DMEM supplemented with 10% (v/v) fetal bovine serum (FBS) and 1% (w/v) penicillin-streptomycin (PS) at 37 °C in a humidified atmosphere of 5% CO_2 in air. The cells were passaged when they reached approximately 80% confluence. (Cells were obtained from Korean Cell Line Bank.)

The experimental procedures regarding mice tissues herein were performed in accordance with protocols approved by The Pohang University of Science and Technology Committee on Animal Research and we followed the guidelines for the use of experimental animals established by The Korean Academy of Medical Science. We made every effort to minimize animal suffering and reduce the number of animals used to prepare samples for imaging. BALB/c type mouse (7 weeks) were used for this experiment. The liver was removed from the mouse after dislocation of the cervical vertebra, washed with PBS buffer, and sliced with a vibrating blade microtome (VT1000S, Leica, Germany) in 1 mm thickness. For the autofluorescence images, the tissue was used without a staining step. For the experiments for **CyOH**, **BRosol**, **BRosam 1**, and **BRosam 2**, tissues were incubated in the PBS buffer solution containing 10 μM of each dye for 1 h in an incubator maintained with 5% of CO_2 in the air and at 37 ˚C. The stained sample was washed with PBS buffer three times to remove the remaining dye on the surface. Each tissues were placed on a slide glass and the images were obtained in depth of 30 μm under confocal microscopy.

3. Cytotoxicity assay.

The cytotoxic effect of the new dyes and probes on the cells was assayed using the Cell Counting Kit-8 (CCK-8, Dojindo Laboratories, Kumamoto, Japan). HeLa cells were seeded into 96-well plates at a density of 5000 cells/well and incubated for 24 h at 37 °C in a humidified atmosphere of 5% CO_2 in air. The probe solution at increasing concentrations was added into the culture media in the plate. The plate was incubated for certain time, and then CCK-8 solution was added to each well of the plate. After the further incubation for 2 h, the absorbance at 450 nm was measured using a microplate reader (Multiskan EX, Thermo Eletron). Results are expressed as the ratio of the absorbance of the positive control over that of the non-treated cells

4. Synthesis

Syntheis of (8-Methoxy-2,3,6,7-tetrahydro-1*H*,5*H*-pyrido[3,2,1-*ij*]quinolin-9-yl)(phenyl)methanone (2):

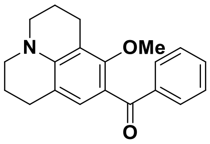

To a solution of $AlCl_3$ (3.28 g, 24.6 mmol) in dry CH_2Cl_2 (60 mL) under Ar atmosphere was added benzyl chloride (3.86 mL, 24.6 mmol) at room temperature. After being stirred for 10 min, the mixture was cooled to 0 ºC. Then a solution of compound **1** (5.0 g, 24.6 mmol) in dry CH_2Cl_2 (20 mL) was added to the mixture dropwise for 4 h.The reaction mixture was poured into cold 5 M HCl (40 mL), and the organic part was collected and washed with brine three times. Then, the solvent was evaporated, and the residues was purified by flash chromatography on silica gel (eluent: hexane/EtOAc = 9:1) to afford the desired product **2** as a yellow solid (2.3 g, 30%): ^{1}H NMR (300 MHz, $CDCl_3$, 293 K, δ): 7.80–7.77 (m, 2H), 7.53–7.47 (m, 1H), 7.44–7.38 (m, 2H), 7.02 (s, 1H), 3.51 (s, 3H), 3.26–3.21 (m, 4H), 2.77–2.67 (m, 4H), and 1.99–1.91 (m, 4H); ^{13}C NMR (75 MHz, $CDCl_3$, 293 K, δ): 195.5, 157.0, 147.0, 140.1, 131.7, 130.4, 129.8, 127.9, 118.4, 115.9, 113.6, 61.7, 50.1, 49.7, 27.5, 21.8, 21.3, and 21.3.

Synthesis of (3,6-Dimethoxynaphthalen-2-yl)(8-methoxy-2,3,6,7-tetrahydro-1*H*,5*H*-pyrido[3,2,1-*ij*]quinolin-9-yl)(phenyl)methanol (4):

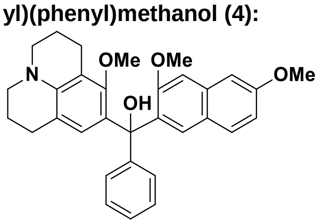

To a solution of compound **3** (1.3 g, 6.91 mmol) dissolved in dry THF (25 mL) under Ar atmosphere, were added TMEDA (4.1 mL, 27.63 mmol) and then *n*-BuLi (6.9 mL, 2 M in Hex, 13.81 mmol) at −10 ºC. The reaction temperature was allowed to rise to room

temperature, and the mixture was further stirred for 6 h. Next, compound **2** (2g, 6.56 mmol) in dry THF (25 mL) was added dropwise to the reaction mixture at −78 °C. The reaction temperature was allowed to rise to room temperature, and it was stirred overnight. The reaction was quenched with water, and the product was extracted with EtOAc. After concentration of the solvent, the crude product was purified by column chromatography on silica gel (eluent: hexane/EtOAc = 9:1) to afford compound **4** (1.0 g, 50%; the staring materials were recovered). The product has limited stability, hence it was directly subjected to the next step.

Synthesis of 13-Hydroxy-9-phenyl-1,2,3,5,6,7-hexahydrobenzo[6,7]chromeno[2,3-*f*]pyrido[3,2,1-*ij*]quinolin-4-ium bromide (BRosol):

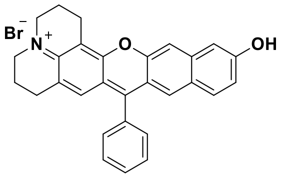

To a solution of compound **4** (1.0 g, 2.02 mmol) in dry CH_2Cl_2 (50 mL) was added a solution of BBr_3 (12 mL, 24.27 mmol, 1.0 M in CH_2Cl_2) at −78 °C dropwise, and the reaction temperature was allowed to rise to room temp., and then the mixture was stirred overnight. Water was added to the reaction mixture, and it was subjected to extractive work-up using CH_2Cl_2. The solvent was condensed and the residue was purified by column chromatography on silica gel (eluent: CH_2Cl_2/MeOH = 19:1) to afford **BRosol** as a dark red solid (0.94g, 95%): ^{1}H NMR (500 MHz, MeOD, 293 K, δ): 7.86 (s, 1H), 7.82 (s, 1H), 7.80–7.78 (d, J = 10.0 Hz, 1H), 7.75–7.73 (m, 3H), 7.52–7.50 (m, 2H), 7.163–7.158 (d, J = 2.5 Hz, 1H), 7.11–7.08 (dd, J = 8.0 Hz, J = 2.5 Hz, 1H), 6.98 (s, 1H), 3.75–3.70 (m, 4H), 3.06–3.04 (t, J = 6.5 Hz, 2H), 2.77–2.75 (t, J = 6.0 Hz, 2H), 2.16–2.12 (m, 2H), and 2.05–2.02 (m, 2H); ^{13}C NMR (125 MHz, MeOD, 293 K, δ): 161.4, 157.0, 156.7, 154.9, 151.0, 140.6, 133.7, 133.0, 132.5, 131.4, 131.0 (2C), 130.1 (2C), 129.4, 129.2, 127.4, 121.2, 119.6, 119.3, 111.6, 108.7, 107.8, 53.4, 52.9, 28.2, 21.5, 20.5, and 20.4. HRMS (EI$^+$) Calcd for $C_{29}H_{24}NO_2^+$ 418.1807; found 418.1805.

Synthesis of 14-bromo-13-hydroxy-9-phenyl-1,2,3,5,6,7-hexahydrobenzo[6,7]chromeno[2,3-*f*]pyrido[3,2,1-ij]quinolin-4-ium bromide (Br-BRosol):

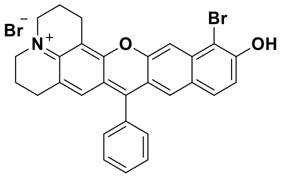

To a solution of **BRosol** (0.01 g, 0.0201 mmol) in dry CH_2Cl_2 (30 mL) was added NBS (0.0036 g, 0.0201 mmol) at room temp., and then the mixture was stirred overnight. Next, the reaction mixture was washed twice with 0.5 M aqueous HBr. The organic phase was condensed, and the residue was subjected to flash chromatography on silica gel (eluent: CH_2Cl_2/MeOH = 10:1) to afford **Br-BRosol** as a red solid (6.3 mg, red solid, 54%): ^{1}H NMR (600 MHz, MeOD, 293 K, δ): 8.30 (s, 1H), 7.98 (s, 1H), 7.88–7.87 (d, J = 9.0 Hz, 1H), 7.79–7.78 (m, 3H), 7.61–7.59 (m, 2H), 7.27–7.26 (d, J = 9.0 Hz, 1H), 7.10 (s, 1H), 3.82–3.77 (m, 4H), 3.19–3.16 (t, J = 6.6 Hz, 2H), 2.84–2.82 (t, J = 6.6 Hz, 2H), 2.23–2.19 (m, 2H), and 2.11–2.07 (m, 2H); ^{13}C NMR (150 MHz, MeOD, 293 K, δ): 157.3, 156.0, 154.9, 152.0, 138.7, 133.6, 132.9, 132.3, 131.5, 131.0 (2C), 130.2 (2C), 129.7, 129.5, 127.9, 120.5, 120.0, 111.7, 108.2, 104.4, 53.6, 53.0, 28.2, 21.5, 20.5 and 20.4.

Synthesis of 9-Phenyl-13-(prop-2-yn-1-yloxy)-1,2,3,5,6,7-hexahydrobenzo[6,7]chromeno[2,3-*f*]pyrido[3,2,1-*ij*]quinolin-4-ium bromide (BRosol-P):

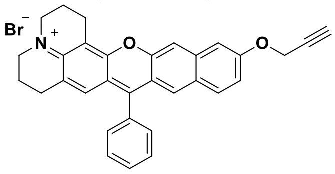

To a solution of **BRosol** (0.01 g, 0.0201 mmol) in CH_3CN (30 mL) was added K_2CO_3 (0.008 g, 0.0603 mmol) at room temp. After being stirred for 15 min, the reaction mixture was treated with propargyl bromide (0.006 mL, 0.0603 mmol) and then the reaction temperature was raised to 50 ˚C. After being stirred for 24 h at 50 °C, the solvent was removed by evaporation and the residue was diluted with CH_2Cl_2. The reaction mixture was washed twice with 0.5 M aqueous HBr, the organic phase was condensed, and the residue was purified by flash chromatography on silica gel (eluent: CH_2Cl_2/MeOH = 19:1) to afford **BRosol-P** as a red solid (7.0 mg, 65%): ^{1}H NMR (500 MHz, MeOD, 293 K, δ): 8.11 (s, 1H), 7.96 (s, 1H), 7.88–7.86 (m, J = 10.0 Hz, 1H), 7.74–7.73 (m, 3H), 7.56–7.55 (m, 2H), 7.511–7.508 (d, J = 1.5 Hz, 1H), 7.23–7.21 (dd, J = 9.5 Hz, J = 2.5 Hz, 1H), 7.083–7.077 (t, J = 1.5 Hz, 1H), 4.964–4.959 (d, J = 2.5 Hz, 2H), 3.79–3.73 (m, 4H), 3.08–3.07 (t, J = 7.5 Hz, 2H), 3.175–3.149 (t, J = 2.5 Hz, 1H), 2.81–2.79 (t, J = 6.5 Hz, 2H), 2.20–2.15 (m, 4H), and 2.07–2.02 (m, 4H); ^{13}C NMR (125 MHz, MeOD, 293 K, δ): 160.9, 155.1, 151.3, 140.1, 133.7, 132.6, 132.3, 131.5, 131.0 (2C), 130.1 (2C), 129.6, 129.5, 128.3, 128.2, 121.5, 120.0, 116.9, 113.6, 112.9, 108.0, 107.2, 79.0, 77.6, 57.1, 53.5, 53.0, 28.3, 21.5, 20.5, and 20.4; HRMS (EI$^+$) Calcd for $C_{32}H_{26}NO_2^+$ 456.1964; found 456.1961.

Synthesis of 13-Acetoxy-9-phenyl-1,2,3,5,6,7-hexahydrobenzo[6,7]chromeno[2,3-*f*]pyrido[3,2,1-*ij*]quinolin-4-ium bromide (BRosol-E):

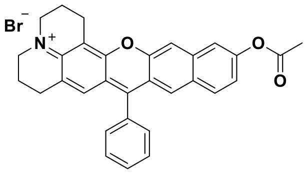

To a solution of **BRosol** (0.01 g, 0.0201 mmol) in CH_3CN (30 mL) was added Et_3N (0.008 mL, 0.0603 mmol) at room temp. After being stirred for 15 min, the reaction mixture was treated with AcCl (0.006 mL, 0.0603 mmol). After being stirred for 24 h at room temp., the reaction mixture was washed twice with 0.5 M aqueous HBr. The organic phase was condensed, and the residue was subjected to flash chromatography on silica gel (eluent: CH_2Cl_2/MeOH = 19:1) to afford **BRosol-E** as a red solid (9.0 mg, red solid, 83%): ^1H NMR (500 MHz, MeOD, 293 K, δ): 8.08 (s, 1H), 8.00 (s, 1H), 7.95–7.94 (m, *J* = 10.0 Hz, 1H), 7.76–7.73 (m, 4H), 7.55–7.53 (m, 2H), 7.32–7.30 (dd, *J* = 9.0 Hz, *J* = 2.0 Hz, 1H), 7.02 (s, 1H), 3.79–3.73 (m, 4H), 3.07–3.04 (t, *J* = 6.3 Hz, 2H), 2.78–2.75 (t, *J* = 6.0 Hz, 2H), 2.16–2.11 (m, 2H), and 2.06–2.01 (m, 2H); ^{13}C NMR (125 MHz, MeOD, 293 K, δ): 170.7, 157.6, 155.5, 154.8, 153.4, 150.8, 138.4, 133.4, 132.3, 132.2, 131.6, 131.1 (2C), 130.23, 130.21 (2C), 129.8, 129.6, 123.6, 122.1, 120.8, 118.9, 113.8, 108.3, 53.7, 53.2, 28.2, 21.4, 21.1, 20.4, and 20.3; HRMS (EI+) Calcd for $C_{32}H_{26}N_2O_3^+$ 460.1913; found 460.1917.

Synthesis of 13-(2,4-Dinitrophenoxy)-9-phenyl-1,2,3,5,6,7-hexahydrobenzo[6,7]chromeno[2,3-*f*]pyrido[3,2,1-*ij*]quinolin-4-ium bromide (BRosol-DNP):

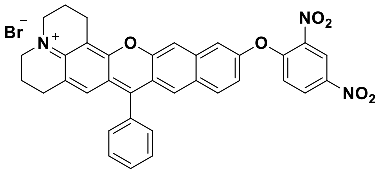

To a solution of **BRosol** (0.01 g, 0.0201 mmol) in CH_3CN (30 mL) was added Et_3N (0.008 mL, 0.0603 mmol) at room temp. After being stirred for 15 min, the reaction mixture was treated with Sanger's reagent (0.003 mL, 0.0241 mmol) and then stirred for 12 h. The solvent was evaporated, and the resulting residue was dissolved with CH_2Cl_2. Then it was washed twice with 0.5 M aqueous HBr, and the organic phase was condensed. The crude product was purified by flash chromatography on silica gel (eluent: CH_2Cl_2/MeOH = 19:1) to afford **BRosol-DNP** as a red solid (10.0 mg, red solid, 75%): ^1H NMR (300 MHz, MeOD, 293 K, δ): 8.96–8.95 (d, *J* = 3.0 Hz, 1H), 8.53–8.49 (dd, *J* = 9.3 Hz, *J* = 3.3 Hz, 2H), 8.15–8.08 (m, 3H), 7.76–7.71 (m, 4H), 7.69–7.56 (m, 2H), 7.43–7.39 (m, 2H), 7.09 (s, 1H), 3.82–3.75 (m, 4H), 3.16–3.11 (t, *J* = 6.3 Hz, 2H), 2.83–2.79 (t, *J* = 6.0 Hz, 2H), 2.20–2.13 (m, 2H), and 2.09–2.01 (m, 2H); ^{13}C NMR (75 MHz, MeOD, 293 K, δ): 157.7, 157.6, 155.6, 155.2, 154.9, 151.3, 144.5, 142.2, 138.98, 134.0, 133.5, 132.5, 131.6, 131.1 (2C), 130.38, 130.32, 130.2 (2C), 129.63, 129.59, 123.1, 122.8, 122.2, 121.3, 120.9, 115.3, 113.8, 108.4, 53.7, 53.2, 28.2, 21.4, 20.4, and 20.4; HRMS (EI+) Calcd for $C_{35}H_{26}N_3O_6^+$ 584.1822; found 584.1824.

Synthesis of 13-((4-Nitrobenzyl)oxy)-9-phenyl-1,2,3,5,6,7-hexahydrobenzo[6,7]chromeno[2,3-*f*]pyrido[3,2,1-*ij*]quinolin-4-ium bromide (BRosol-NBE):

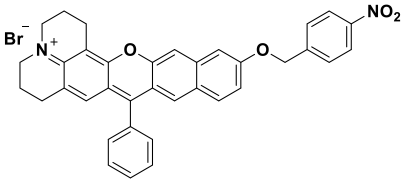

To a solution of **BRosol** (0.01 g, 0.0201 mmol) in CH_3CN (30 mL) was added K_2CO_3 (0.008 g, 0.0603 mmol) at room temp. After being stirred for 15 min, the reaction mixture was treated with 4-nitrobenzyl bromide (0.013 g, 0.0603 mmol) at room temp. After being stirred for 12 h at room temp., the solvent was removed by evaporation and the residue was diluted with CH_2Cl_2. The mixture was washed twice with 0.5 M aqueous HBr, and the organic phase was condensed. The residue was purified by flash chromatography on silica gel (eluent: CH_2Cl_2/MeOH = 19:1) to afford **BRosol-NBE** as a red solid (7.0 mg, red solid, 55%): ^1H NMR (500 MHz, MeOD, 293 K, δ): 8.21–8.19 (d, *J* = 8.5 Hz, 1H), 7.98 (s, 1H), 7.91 (s, 1H), 7.83–7.81 (d, *J* = 9.0 Hz, 1H), 7.76–7.74 (m, 3H), 7.66–7.64 (d, *J* = 8.5 Hz, 2H), 7.56–7.54 (m, 2H), 7.371–7.366 (d, *J* = 2.5 Hz, 1H), 7.18–7.16 (dd, *J* = 9.0 Hz, *J* = 2.5 Hz, 1H), 7.03 (s, 1H), 5.24 (s, 2H), 3.78–3.72 (m, 4H), 3.10–3.07 (t, *J* = 6.5 Hz, 2H), 2.78–2.75 (t, *J* = 6.5 Hz, 2H), 2.19–2.14 (m, 2H), and 2.06–2.01 (m, 2H); ^{13}C NMR (75 MHz, MeOD, 293 K, δ): 161.3, 157.2, 156.4, 154.8, 151.1, 148.7, 145.4, 140.1, 133.5, 132.7, 132.2, 131.5 (2C), 131.0 (2C), 130.2, 129.6, 129.4, 128.7 (2C), 128.1, 124.6 (2C), 121.5, 120.3, 119.8, 112.8, 107.9, 106.9, 69.8, 53.5, 53.0, 28.2, 21.4, 20.5, and 20.4; HRMS (EI+) Calcd for $C_{36}H_{29}N_2O_4^+$ 553.2127; found 553.2124.

Synthesis of 9-Phenyl-13-(((trifluoromethyl)sulfonyl)oxy)-1,2,3,5,6,7-hexahydrobenzo[6,7]chromeno[2,3-*f*]pyrido[3,2,1-*ij*]quinolin-4-ium bromide (BRosol-triflate):

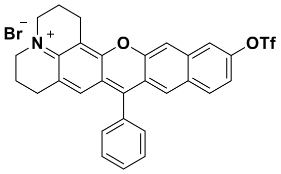

To a solution of **BRosol** (0.01 g, 0.0201 mmol) in CH_3CN (30 mL) was added Et_3N (0.006 mL, 0.0603 mmol) at room temp. After being stirred for 15 min, the reaction mixture was treated with Tf_2O (0.005 mL, 0.0603 mmol) at room temp., and then it was further stirred for 1 h. The solvent was evaporated, and the residue was diluted with CH_2Cl_2 and then washed twice with 0.5 M aqueous HBr. The organic phase was condensed, and the residue was subjected to flash chromatography on silica gel (eluent: CH_2Cl_2/MeOH = 19:1) to afford **BRosol-triflate** as a red solid (10.0 mg, red solid, 79%): ^{1}H NMR (500 MHz, MeOD, 293 K, δ): 8.317 (s, 1H), 8.14–8.11 (m, 3H), 7.75–7.74 (m, 3H), 7.58–7.56 (m, 2H), 7.51–7.48 (dd, *J* = 9.5 Hz, *J* = 2.5 Hz, 1H), 7.10 (s, 1H), 3.84–3.78 (m, 4H), 3.18–3.16 (t, *J* = 6.5 Hz, 2H), 2.83–2.80 (t, *J* = 6.0 Hz, 2H), 2.21–2.16 (m, 2H), and 2.09–2.04 (m, 2H); ^{13}C NMR (125 MHz, MeOD, 293 K, δ): 158.1, 154.9, 154.8, 151.4, 151.2, 137.8, 133.9, 133.3, 132.4, 131.6, 131.1 (2C), 130.8, 130.7, 130.3 (2C), 129.7, 123.5, 121.7, 121.5, 121.4, 119.8, 118.9, 114.7, 108.7, 53.9, 53.4, 28.2, 21.4, 20.4, and 20.3; HRMS (EI+) Calcd for $C_{30}H_{23}F_3NO_4S^+$ 550.1300; found 550.1302.

Synthesis of 13-Amino-9-phenyl-1,2,3,5,6,7-hexahydrobenzo[6,7]chromeno[2,3-*f*]pyrido[3,2,1-*ij*]quinolin-4-ium-9-phenyl-13-(((trifluoromethyl)sulfonyl)oxy)-1,2,3,5,6,7-hexahydrobenzo[6,7]chromeno[2,3-*f*]pyrido[3,2,1-*ij*]quinolin-4-ium bromide (BRosam 1):

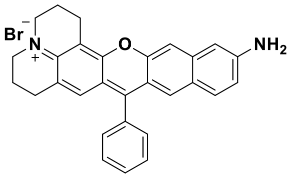

To a solution of **BRosol- triflate** (0.01 g, 0.0159 mmol), $Pd_2(dba)_3$ (0.002 g, 0.00159 mmol), xantphos (0.003 g, 0.00476 mmol), and Cs_2CO_3 (0.016 g, 0.0476 mmol) in toluene (4 mL), which was kept in a sealed tube, was added benzophenone imine (0.011 mL, 0.0636 mmol) and the reaction mixture was stirred for 12 h at 100 ^{o}C. The toluene was evaporated, the residue was diluted with CH_2Cl_2 and then washed twice with 0.5 M aqueous HBr. The organic phase was condensed, and the residue was purified by flash chromatography on silica gel (eluent = CH_2Cl_2/MeOH =19:1) to afford compound **5** (rose color), which was directly used for the next step.

A solution of compound **5** in MeOH (~0.1 M), NaOAc (2.4 equiv.), and hydroxylamine hydrochloride (1.8 equiv.) was stirred at room temperature for 1 h. Then the solvent was evaporated, and the residue was purified by flash chromatography on silica gel (eluent: CH_2Cl_2/MeOH = 9:1) to afford **BRosam 1** as a blue solid (4.0 mg, 51%): ^{1}HNMR (300 MHz, MeOD, 293 K, δ): 7.73–7.70 (m, 4H), 7.65–7.63 (m, 2H), 7.52–7.50 (m, 2H), 7.01–6.99 (dd, *J* = 9.0 Hz, *J* = 2.0 Hz, 1H), 6.97 (s, 1H), 6.904–6.899 (d, *J* = 2.5 Hz, 1H), 3.71–3.65 (m, 4H), 3.10–3.08 (t, *J* = 6.5 Hz, 2H), 2.76–2.74 (t, *J* = 6.5 Hz, 2H), 2.16–2.10 (m, 2H), and 2.04–1.99 (m, 2H); ^{13}C NMR (125 MHz, MeOD, 293 K, δ): 153.6, 151.7, 142.0, 134.0, 133.0, 132.9, 132.8, 131.3, 130.96, 130.94, 130.93 (2C), 130.0 (2C), 129.2, 127.9, 126.6, 121.0, 118.2, 117.9, 109.5, 107.4, 105.0, 53.0, 52.5, 49.5, 49.3, 49.2, 49.0, 48.80, 48.70, 48.50, 28.3, 21.6, and 20.6; HRMS (EI+) Calcd for $C_{29}H_{25}N_2O^+$ 417.1967; found 417.1968.

Synthesis of 13-(Dimethylamino)-9-phenyl-1,2,3,5,6,7-hexahydrobenzo[6,7]chromeno[2,3-*f*]pyrido[3,2,1-*ij*]quinolin-4-ium bromide (BRosam 2):

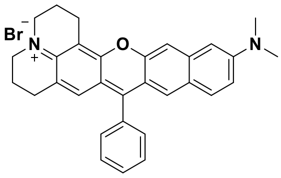

To a reaction mixture of **BRosol-triflate** (0.01 g, 0.0159 mmol), $Pd_2(dba)_3$ (0.002 g, 0.00159 mmol), xantphos (0.003 g, 0.00476 mmol), Cs_2CO_3 (0.016 g, 0.0476 mmol) in toluene (4 mL) in a sealed tube, was added dimethylamine (0.040 mL, 2.0 M in THF solution, 0.0795 mmol). The reaction mixture was kept stirring for 12 h at 100 ^{o}C, and then the solvent was evaporated. The resulting residue was diluted with CH_2Cl_2 and then washed twice with 0.5 M aqueous HBr. The organic phase was condensed, and the residue was purified by flash chromatography on silica gel (eluent: CH_2Cl_2/MeOH =19:1) to afford **BRosam 2** as a blue solid (4.0 mg, 48%): ^{1}HNMR (500 MHz, MeOD, 293 K, δ): 7.78–7.71 (m, 6H), 7.54–7.51 (m, 2H), 7.29–7.25 (dd, *J* = 15.5 Hz, *J* = 4.0 Hz, 1H), 3.72–3.65 (m, 4H), 3.21 (s, 6H), 3.13–3.09 (t, *J* = 6.0 Hz, 2H), 2.77–2.73 (t, *J* = 5.5 Hz, 2H), 2.18–2.01 (m, 2H), and 2.05–1.97 (m, 2H); ^{13}C NMR (125 MHz, MeOD, 293 K, δ): 157.5, 156.1, 155.0, 151.8, 141.5, 134.1, 132.8, 132.7, 131.2, 131.0, 131.0 (2C), 130.0 (2C), 129.2, 127.9, 126.1, 118.5, 118.1, 117.9, 110.1, 107.5, 104.0, 53.0, 52.5, 49.5, 49.3, 49.2, 49.0, 48.8, 48.7, 48.5, 40.4, 28.3, 21.6, and 20.6; HRMS (EI+) Calcd for $C_{31}H_{29}N_2O^+$ 445.2280; found 445.2283.

Synthesis of 13-((((4-Nitrobenzyl)oxy)carbonyl)amino)-9-phenyl-1,2,3,5,6,7-hexahydrobenzo[6,7]chromeno[2,3-*f*]pyrido[3,2,1-*ij*]quinolin-4-ium (BRosam-*p*NBC):

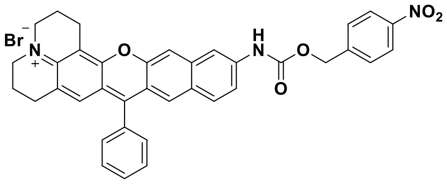

To a solution of **BRosam 1** (0.01 g, 0.0201 mmol), 4-nitrobenzyl chloroformate (0.013 g, 0.0603 mmol), and DMAP (0.002 g, 0.0201 mmol) in CH_2Cl_2 (30 mL), was added pyridine (0.016 mL, 0.201 mmol). The resulting mixture was keep stirring for overnight at room temperature, and then it was washed with 0.5 M HBr twice. The solvent was evaporated, and the residue was subjected to flash chromatography on silica gel (eluent: CH_2Cl_2/MeOH = 19:1) to afford **BRosam-*p*NBC** as a red solid (11.0 mg, 86%): ^{1}HNMR (500 MHz, MeOD, 293 K, δ): 8.43 (s, 1H), 8.29–8.27 (d, J = 8.5 Hz, 1H), 8.13–8.11 (d, J = 9.0 Hz, 1H), 8.03 (s, 1H), 7.96–7.94 (d, J = 9.0 Hz, 1H), 7.75–7.70 (m, 5H), 7.58–7.56 (m, 2H), 7.09 (s, 1H), 5.40 (s, 2H), 3.80–3.75 (m, 4H), 3.17–3.15 (t, J = 6.0 Hz, 2H), 2.81–2.78 (t, J = 6.0 Hz, 2H), 2.20–2.15 (m, 2H), and 2.06–2.02 (m, 2H); ^{13}C NMR (150 MHz, MeOD, 293 K, δ): 157.8, 155.2, 154.9, 151.9, 149.2, 145.2, 140.0, 137.0, 133.4, 132.7, 131.6 (2C), 131.1 (2C), 130.4, 130.2, 129.9, 129.60 (2C), 129.58, 124.7 (2C), 123.6, 122.0, 121.1, 113.4, 112.0, 108.5, 67.1, 53.8, 53.3, 28.2, 21.4, 20.5, and 20.4; HRMS (EI+) Calcd for $C_{37}H_{30}N_3O_5^+$ 596.2185; found 596.2188.

5. Tables

Table S1. Selected reaction-based fluorescent probes based on cyanine and hemicyanine dyes.

Probe	Analyte	Product	Ref.
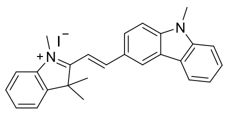	HSO_3^-	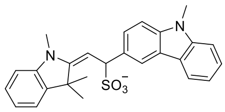	*Chem. Commun.* **2015**, *51*, 10236–10239
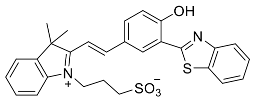	HSO_3^-	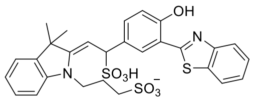	*Anal. Chem.* **2016**, *88*, 4426–4431
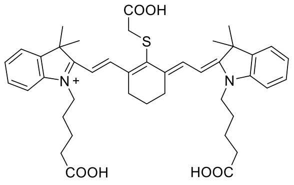	$ONOO^-$ or ClO^-	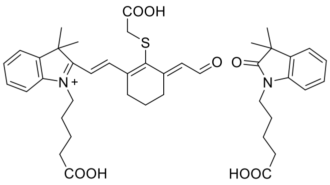	*Angew. Chem., Int. Ed.* **2017**, *56*, 4165–4169
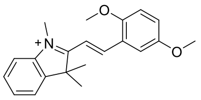	HSO_3^-	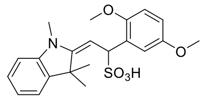	*Talanta*, **2017**, *165*, 625–631
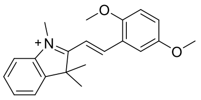	HClO	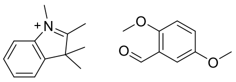	*Talanta*, **2017**, *165*, 625–631
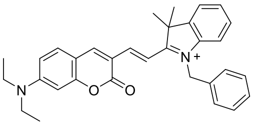	H_2S	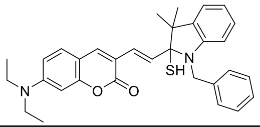	*Angew. Chem., Int. Ed.* **2013**, *52*, 1688–1691

Table S2. Photophysical properties of **Brosol-DNP**, **Brosol-NBE**, **Brosam-pNBC** and **Br-BRosol**..

Dye[a]	λ_{abs} (nm)	λ_{em} (nm)	Stokes shift (nm)	ε	Φ_f[b]
**BRosol-DNP**	460	595	135	25500	0.050
**BRosol-NBE**	480	590	110	19100	0.091
**BRosam-pNBC**	470	597	75	13760	0.046
**Br-BRosol**	520	586	66	17420	0.016
	615	732	117	17920	0.008

[a] The concentration of each dye was 10 µM (containing 1% DMSO) in the 30%EtOH/PBS (pH 7.4). [b] Fluorescence quantum yields determined using Nile blue (Φ_F = 0.27 in EtOH) as a reference dye.

Table S3. Comparision of **CyOH** and **BRosol** of their imaging performance in cell and tissue.

	**CyOH**	**BRosol**	Note
Mouse liver tissue	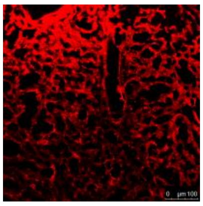	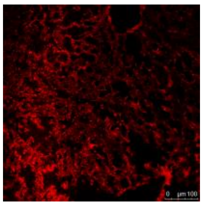	Dye: 10 µM<br>Incubation time: 15 min<br>Laser: 633 nm (5%)<br>Window: 650 – 800 nm<br>Scale bar: 100 µm
A549 cell	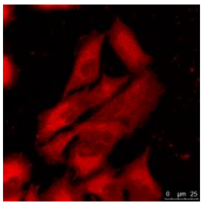	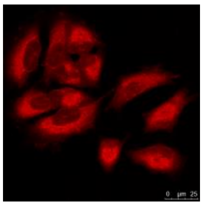	Dye: 10 µM<br>Incubation time: 15 min<br>Laser: 633 nm (5%)<br>Window: 650 – 800 nm<br>Scale bar: 25 µm
HT-29 cell	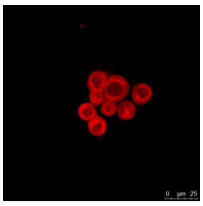	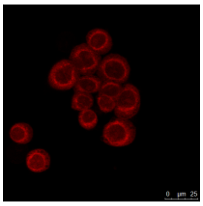	Dye: 10 µM<br>Incubation time: 15 min<br>Laser: 633 nm (5%)<br>Window: 650 – 800 nm<br>Scale bar: 25 µm

Note: the details of tissue and cell preparation were providide in the "**Experiment scetion**" of the manuscript and " **Cell culturing and tissue experiments**" of ESI.

6. Figures

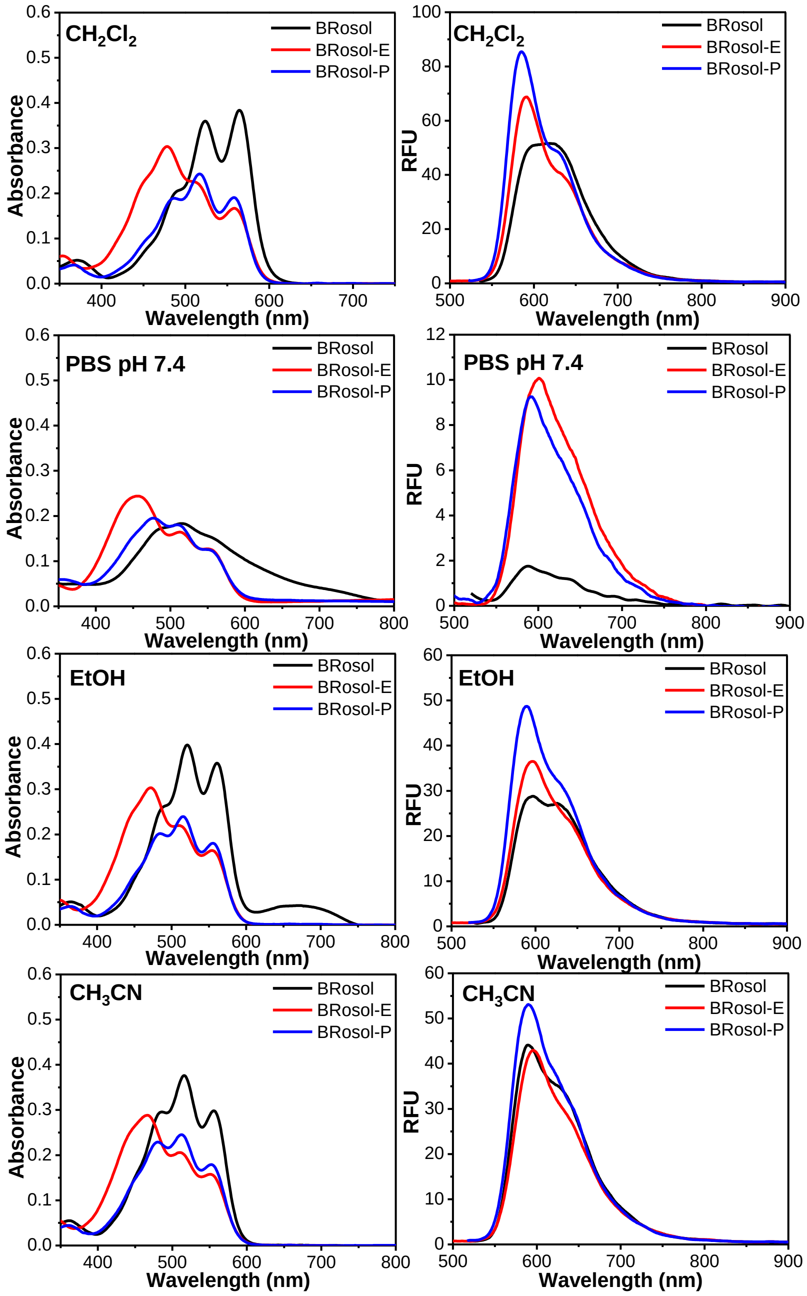

Figure S1. Photophysical properties of **BRosol**, **BRosol-E** and **BRosol-P** in different solvents (10 μM dye in the given solvent containing 1% DMSO), obtained under excitation at the absorption maximum of each dye.

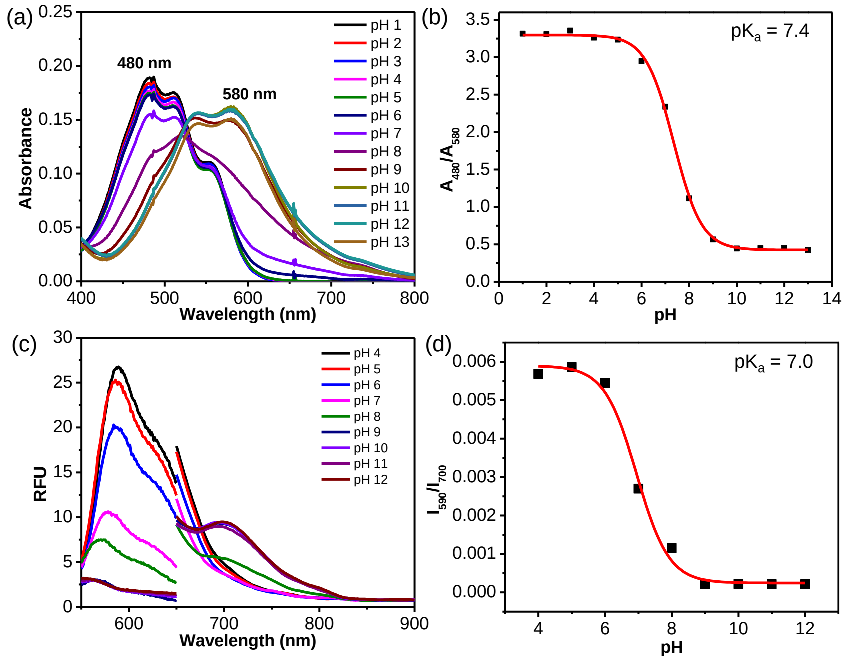

Figure S2. pH-Dependent absorption and emission spectral changes of **BRosol** in UBS at pH 1–13.

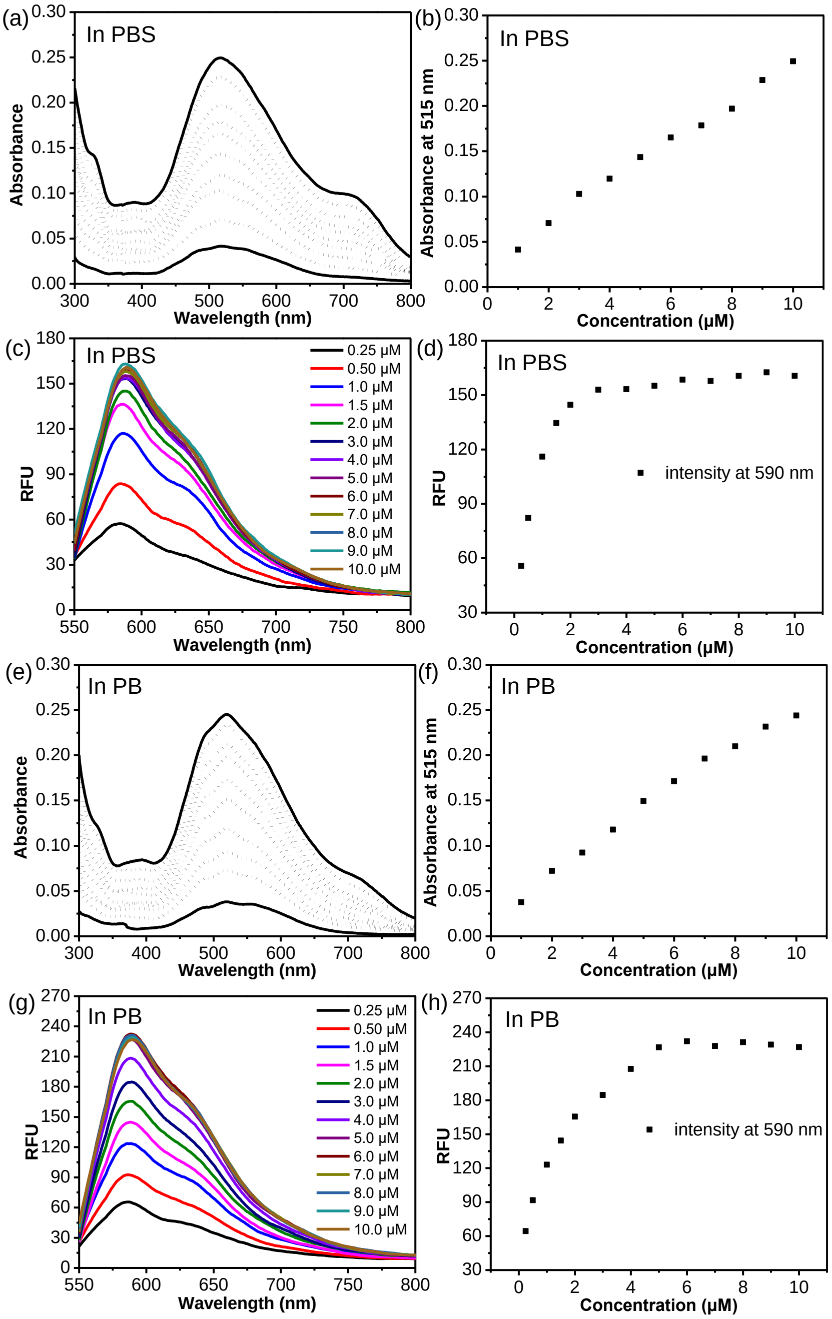

Figure S3. Concentration based absorbance and fluorescent intensity study of **BRosol** in PBS (phosphate buffer saline, 10 mM, pH 7.4) and PB (phosphate buffer, 10 mM, pH 7.4).

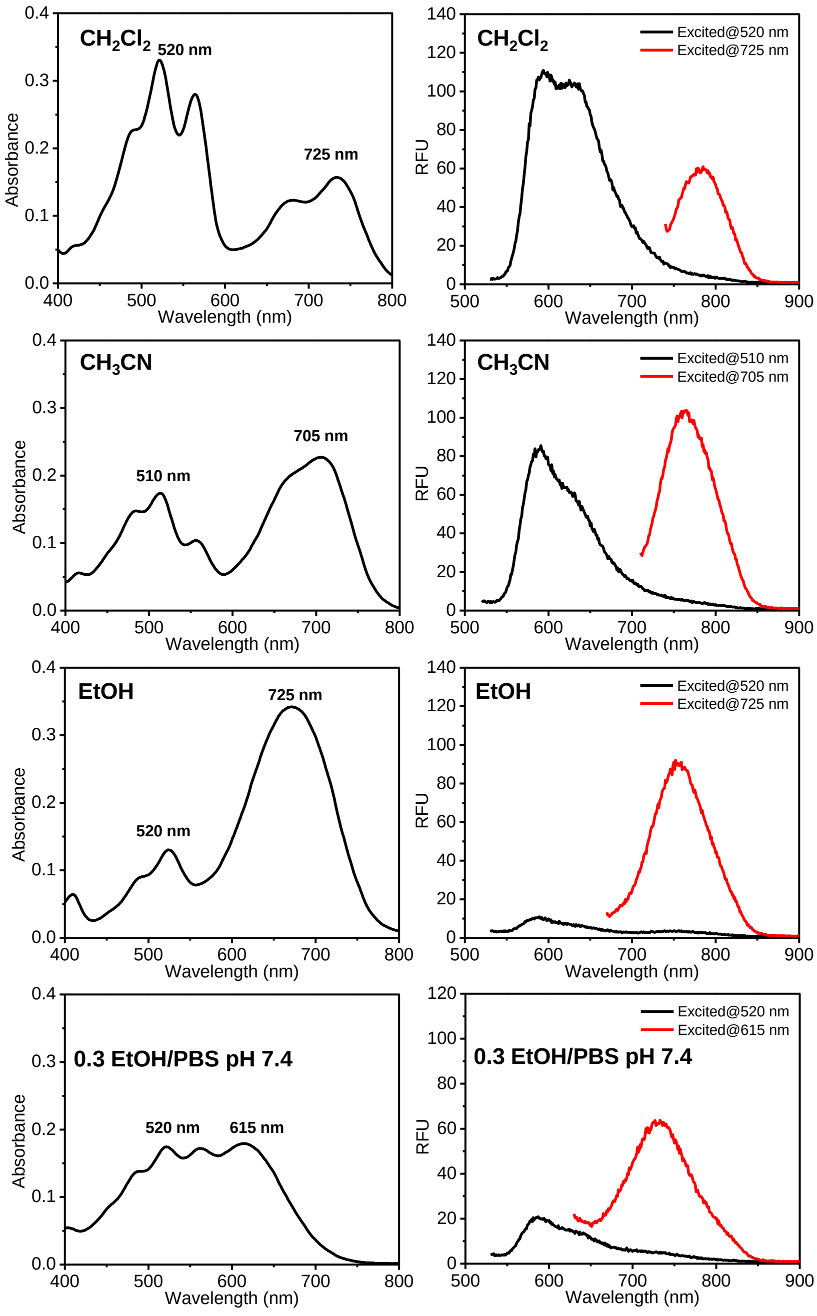

Figure S4. Photophysical properties of **Br-BRosol** in different solvents (10 μM in the given solvent containing 1% DMSO), obtained under excitation at the absorption maximum.

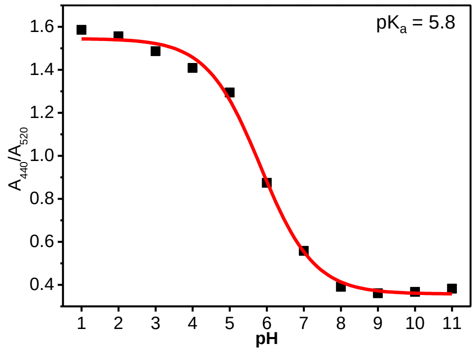

Figure S5. pKa study of **Br-BRosol.**

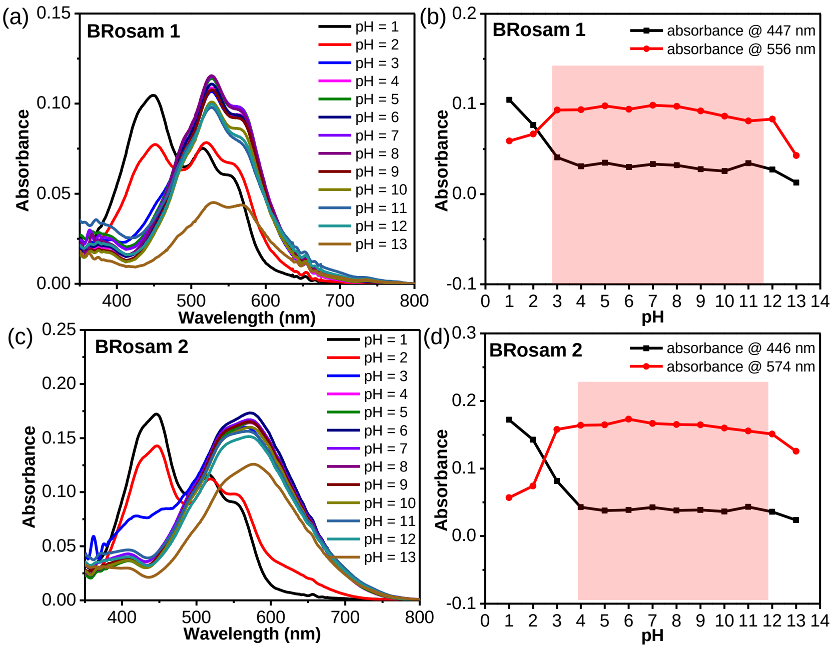

Figure S6. Absorption spectra of **BRosam 1** (a and b) and **BRosam 2** (c and d) in different pHs, obtained for each dye at 10 μM in UBS (containing 1% DMSO).

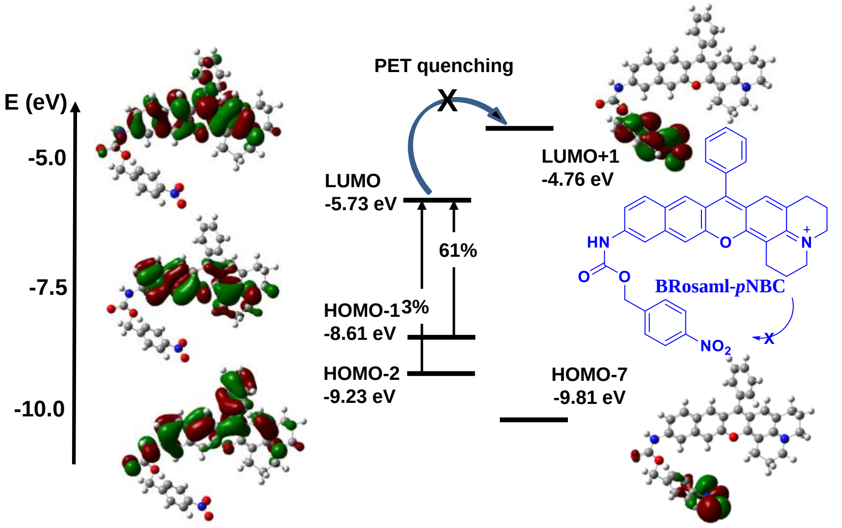

Electronic transition[a]	Energy (nm eV^{-1})	Experimental absorption (nm)	f[b]	Composition	[c]Contribution
$S_0 \rightarrow S_1$	550 / 2.25	520	0.1794	HOMO-1 → LUMO	34%
				HOMO → LUMO	65%
$S_0 \rightarrow S_2$	477 / 2.60	470	0.6694	HOMO-2 → LUMO	3%
				HOMO-1 → LUMO	61%

[a]Only the selected excited states are presented. [b]Oscillator strength. [c]Contribution of the respective excitation to the total CI wavefunction

Figure S7. Frontier molecular orbitals of **BRosol-*p*NBC** obtained by TD-DFT calculations using Gaussian'09 equipped with hybrid B3LYP functional and 6-31 G(d,p) basis set.

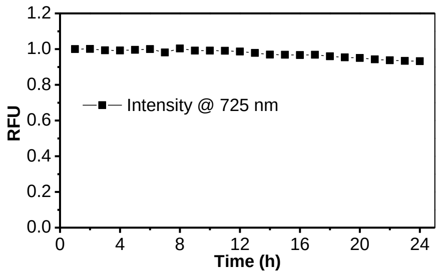

Figure S8. Time-dependent fluorescent intensity changes of **BRosol** (10 μM) in 30% EtOH/PBS (100 mM, pH 7.4) at 37 ℃. λ_{ex} = 615 nm; λ_{em} = 725 nm.

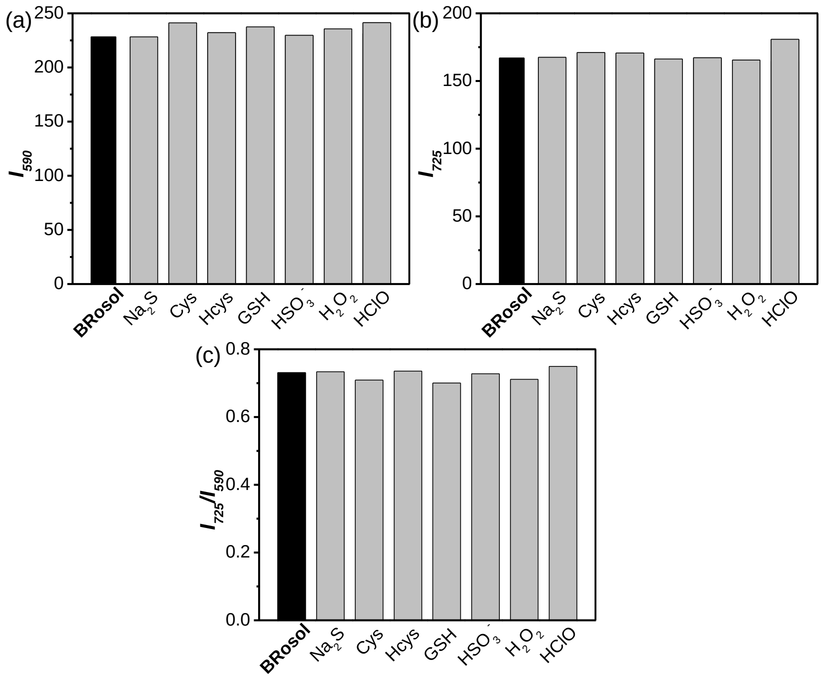

Figure S9. Chemical stability data of **BRosol** (10 μM) against potentially reactive biological species, estimated by the fluorescent changes observed upon addition of biothiols (Na_2S, Cys, Hcy, and GSH), bisulfite (HSO_3^-), and reactive oxygen species (H_2O_2 and HClO), each species at 100 μM in 30% EtOH/PBS (100 mM, pH 7.4) after 1 h incubation: (a, b) The emission intensity at 725 nm or at 590 nm (I_{725} and I_{590}); (c) the intensity ratio (I_{725}/I_{590}) changes under dual excitation at 615 nm (slit: 12 nm/12 nm) and at 515 nm (slit: 4 nm/4 nm).

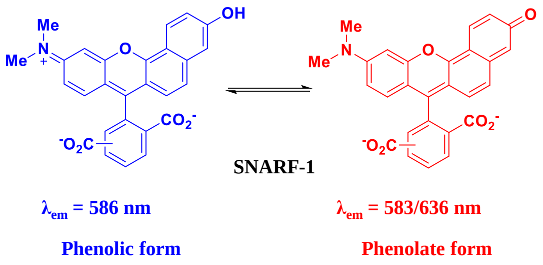

Figure S10. SNARF-1 in the phenolic and phenolate forms.

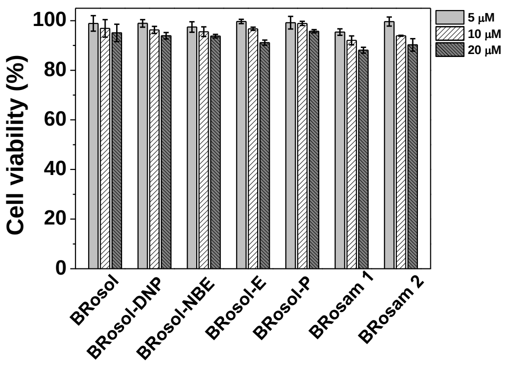

Figure S11. HeLa cell viability data upon incubation with new dyes, observed up to 12 h at varying dye concentrations (5, 10, and 20 μM).

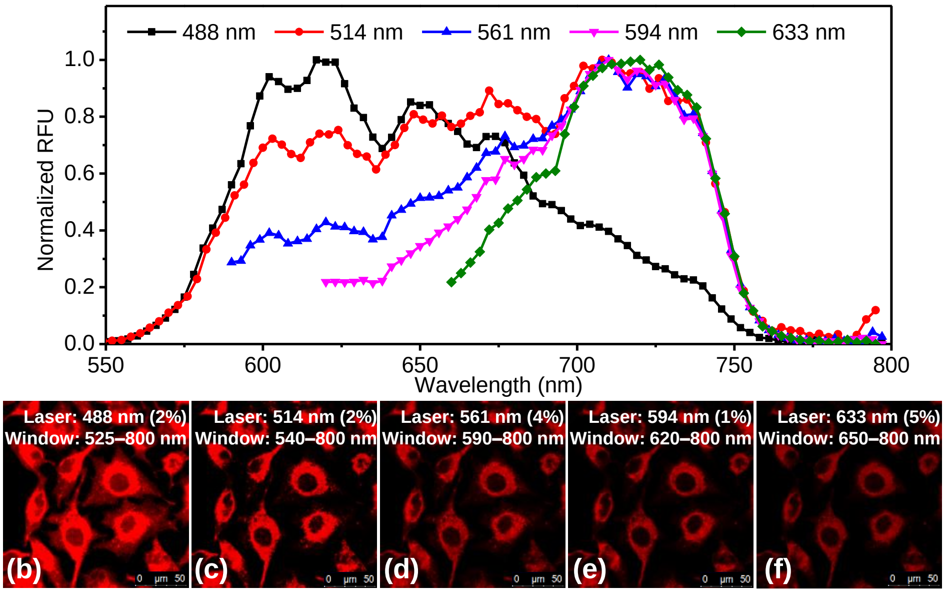

Figure S12. The cellular emission spectra and fluorescent images of **BRosol**. (a) Emission spectra of **BRosol** in HeLa cells, obtained at different excitation wavelengths (λ_{ex} = 488, 514, 561, 594, and 633 nm). (b)–(f) Fluorescent cellular images obtained by confocal laser scanning microscopy: (b) λ_{ex} = 488 nm (2% laser power), emission window = 525–800 nm; (c) λ_{ex} = 514 nm (2% laser power), emission window = 540–800 nm; (d) λ_{ex} = 561 nm (4% laser power), emission window = 590–800 nm; (e) λ_{ex} = 594 nm (1% laser power), emission window = 620–800 nm; (f) λ_{ex} = 633 nm (5% laser power), emission window = 650–800 nm. The HeLa cells were incubated with **BRosol** (10 μM) in PBS (pH 7.4) containing 1% DMSO for 30 min and then fixed with 4% formaldehyde, prior to taking the emission spectra and images.

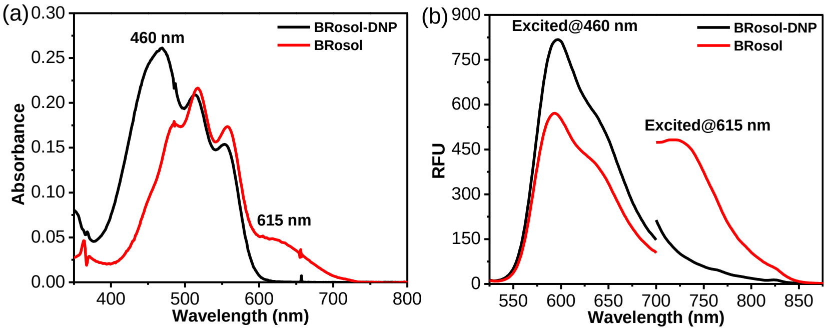

Figure S13. (a) Absorption spectra of **BRosol** and **BRosol-DNP** (each at 10 μM in 30% EtOH/PBS containing 1% DMSO) and (b) their emission spectra obtained under dual excitation at 460 nm (slits: 4 nm/4 nm) and 615 nm (slits: 12 nm/12 nm), respectively.

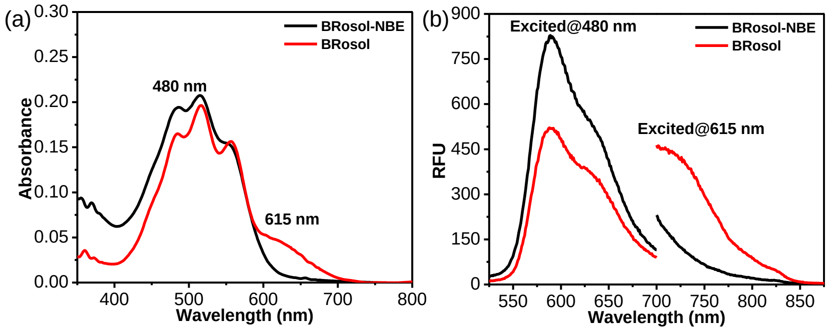

Figure S14. (a) Absorption and (b) emission spectra of **BRosol** and **BRosol-NBE** (each at 10 μM in 30% EtOH/PBS containing 1% DMSO), obtained under excitation at 460 nm (slits: 4 nm/4 nm) and 615 nm (slit: 12 nm/12 nm), respectively.

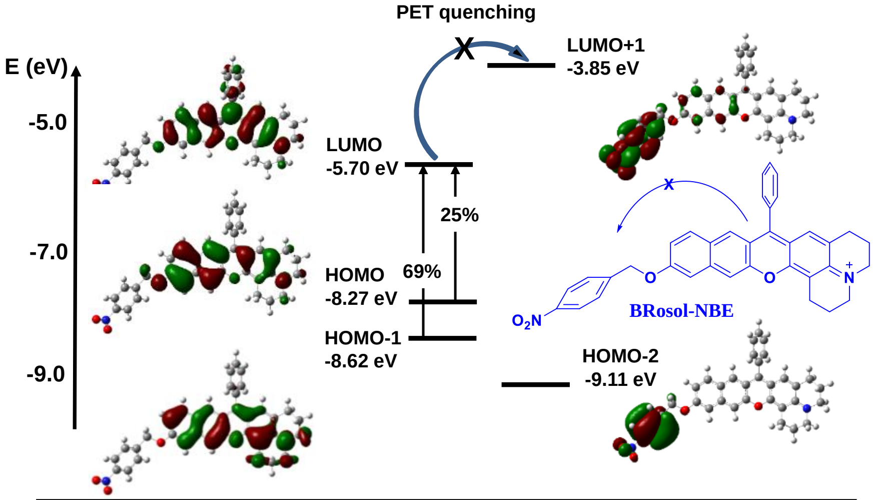

Electronic transition[a]	Energy (nm eV^{-1})	Experimental absorption (nm)	f[b]	Composition	[c]Contribution
$S_0 \rightarrow S_1$	561 / 2.21	516	0.1617	HOMO-1 → LUMO	26%
				HOMO → LUMO	73%
$S_0 \rightarrow S_2$	459 / 2.65	480	0.7718	HOMO-1 → LUMO	69%
				HOMO → LUMO	25%

[a]Only the selected excited states are presented. [b]Oscillator strength. [c]Contribution of the respective excitation to the total CI wavefunction

Figure S15. Frontier molecular orbitals of **BRosol-NBE** obtained by TD-DFT calculations using Gaussian'09 equipped with hybrid B3LYP functional and 6-31 G(d,p) basis set.

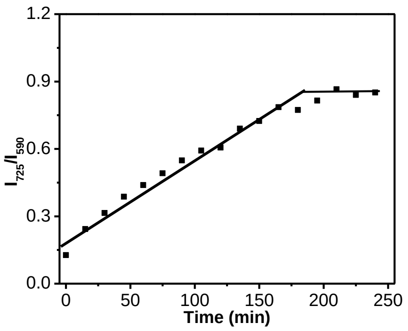

Figure S16. The emission intensity ratio, $I_{725\,nm}$ (slits: 12 nm/12 nm)/$I_{590\,nm}$ (slits: 4 nm/4 nm), changes during the fluorescence titration of **BRosol-DNP** (10 μM) with GSH (10 mM) in 30% EtOH/PBS (100 mM, pH 7.4; containing 1% DMSO).

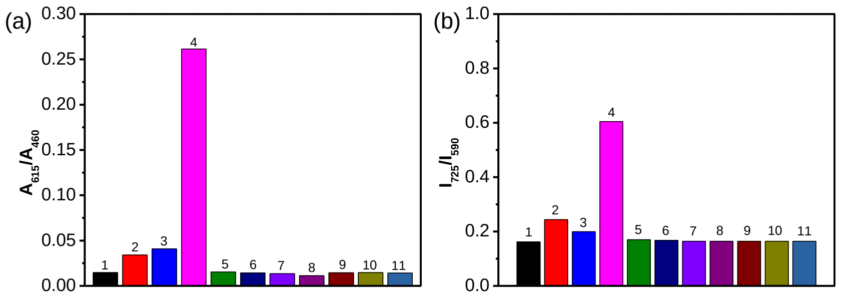

Figure S17. (a) Absorbance ratio (A_{615}/A_{460}) changes and (b) emission ratio ($I_{725\text{ nm}}$ (slits: 12 nm/12 nm)/$I_{590\text{ nm}}$(slits: 4 nm /4 nm)) changes of **BRosol-DNP** (10 μM), measured 2 h after addition of different analytes in 30% EtOH/PBS (100 mM, pH 7.4) at 37 °C: 1, probe only; 2, Na_2S (100 μM); 3, Cys (200 μM); 4, GSH (10 mM); 5, Hcys (50 μM); 6, $S_2O_3^{2-}$; 7, SO_4^{2-}; 8, NO_3^-; 9, NO_2^-; 10, HCO_3^-; 11, Cl^- (at a biologically relevant concentration of the biothiols; at 1.0 mM for other analytes).

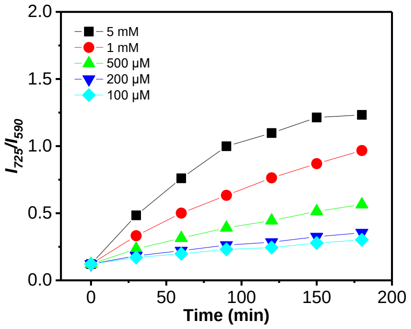

Figure S18. Time-course of the fluorescence response of **BRosol-DNP** (10 μM) toward GSH at different concentrations in 30% EtOH/PBS (100 mM, pH 7.4) containing 1% DMSO: The intensity ratio ($I_{725\text{ nm}}$ (slit = 12 nm/12 nm)/$I_{590\text{ nm}}$ (slit = 4 nm/4 nm)) changes dependent on time are shown.

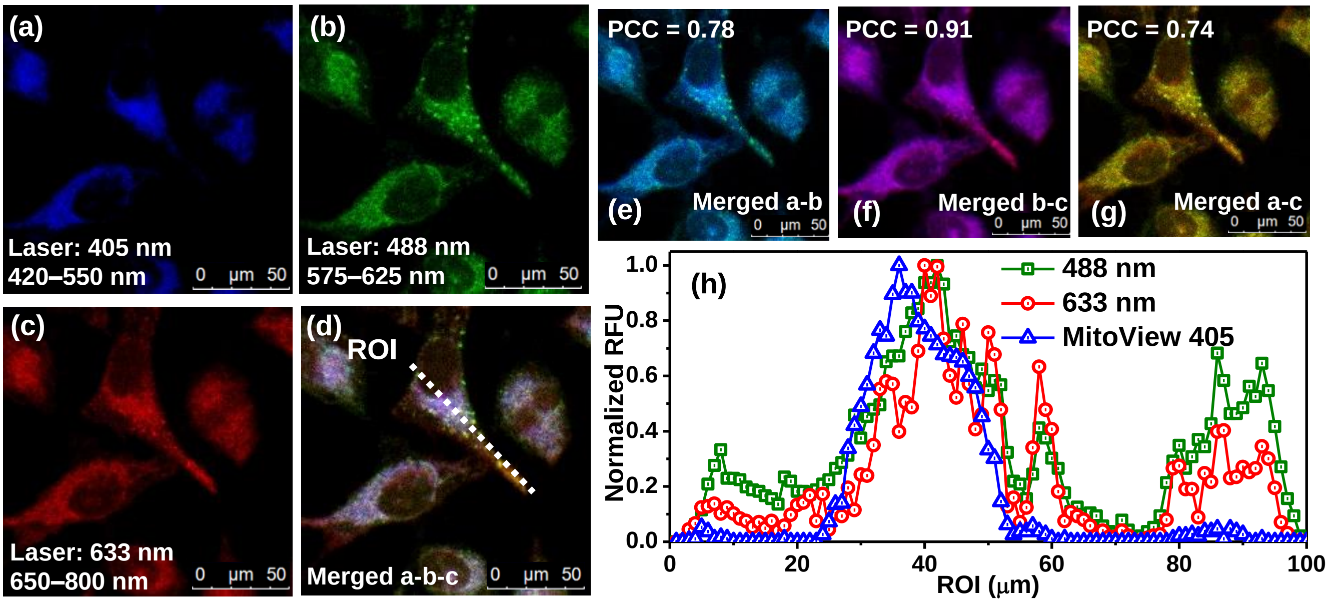

Figure S19. Zoomed figure 6.

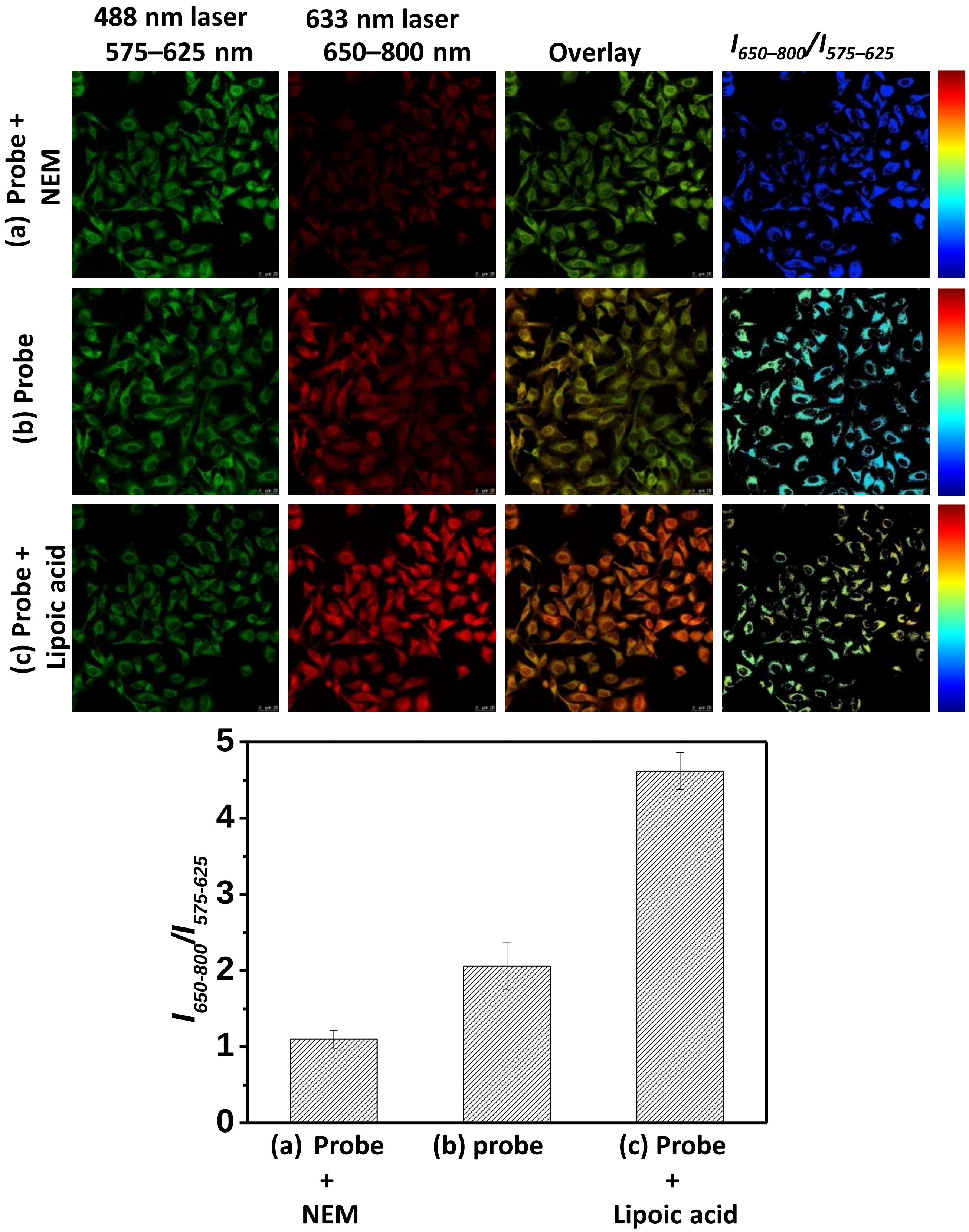

Figure S20. Zoomed figure 10.

7. Spectra

^{1}H NMR of compound **2**.

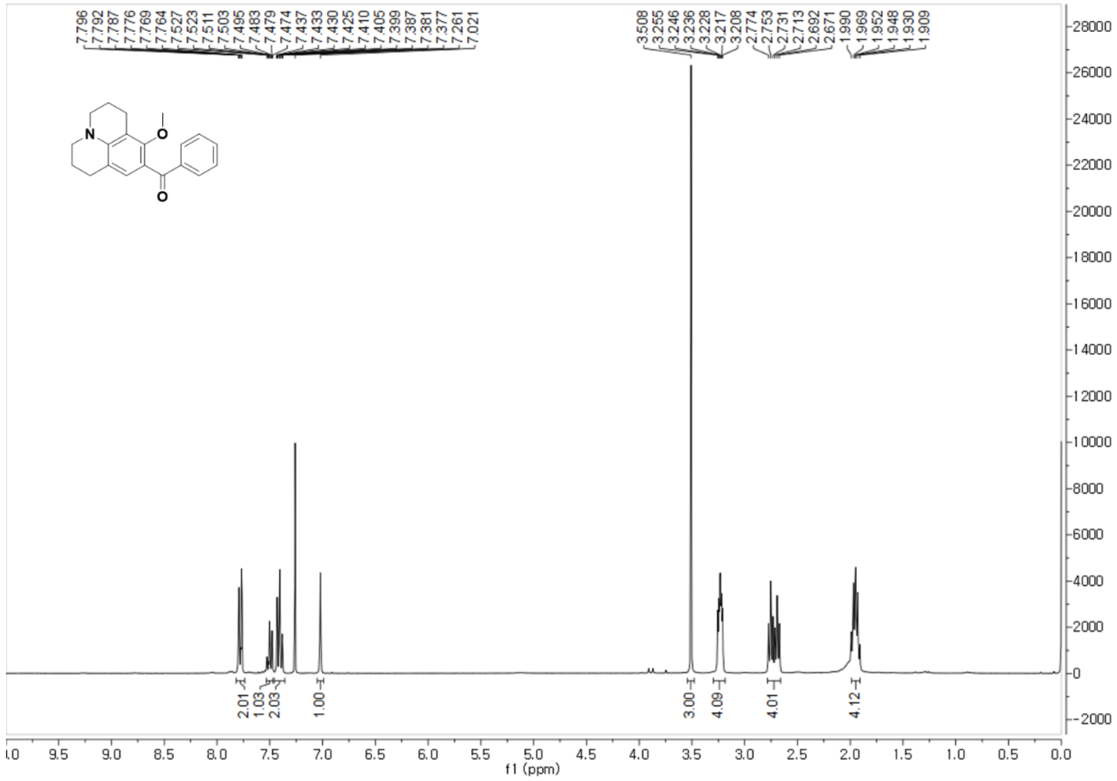

^{13}C NMR of compound **2**.

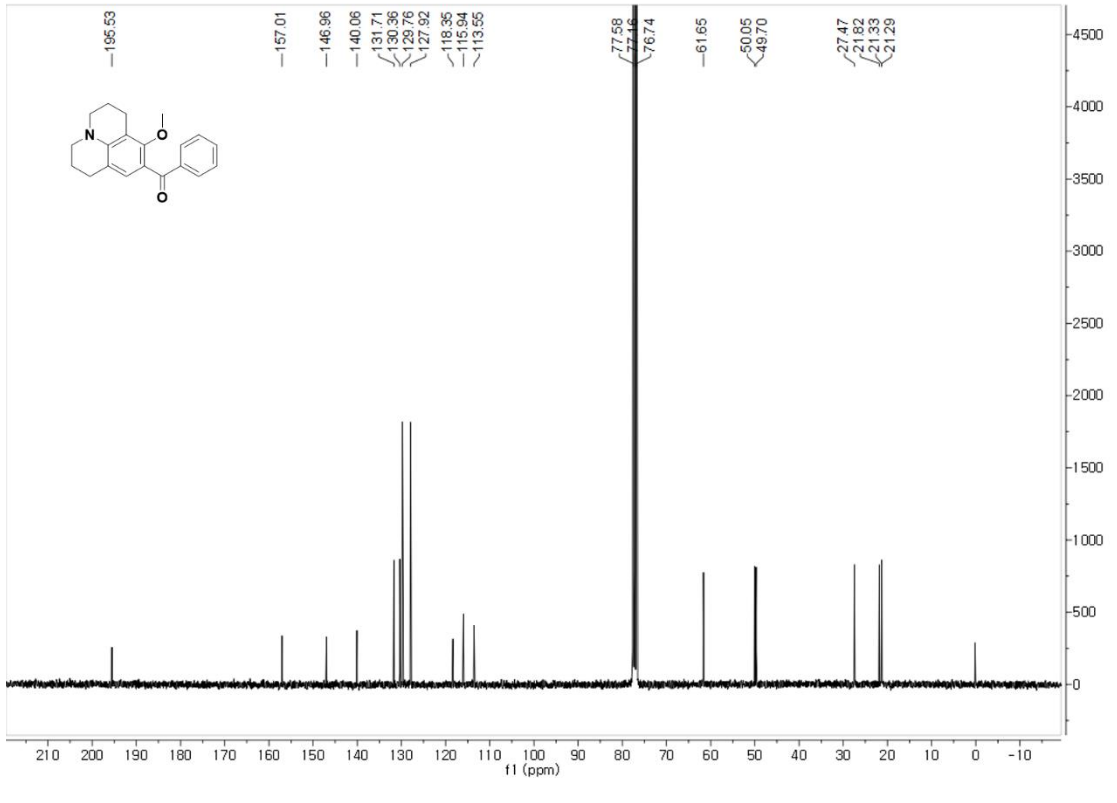

^{1}H NMR of **BRosol**..

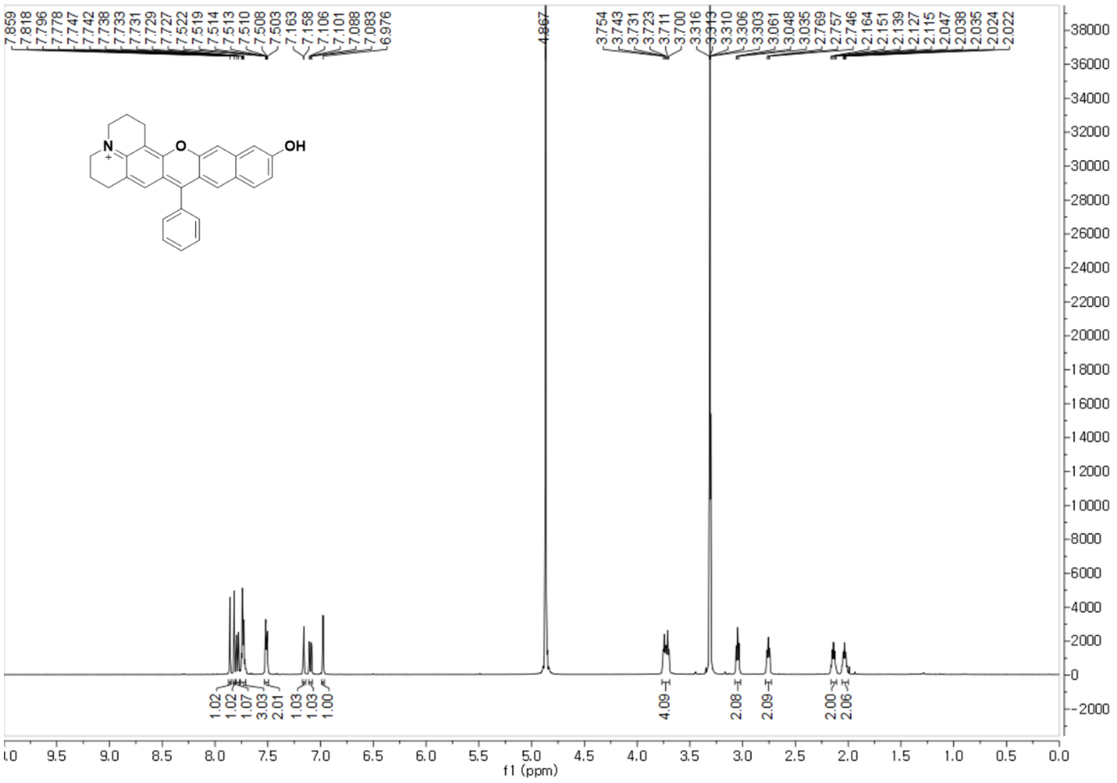

^{13}C NMR of **BRosol**.

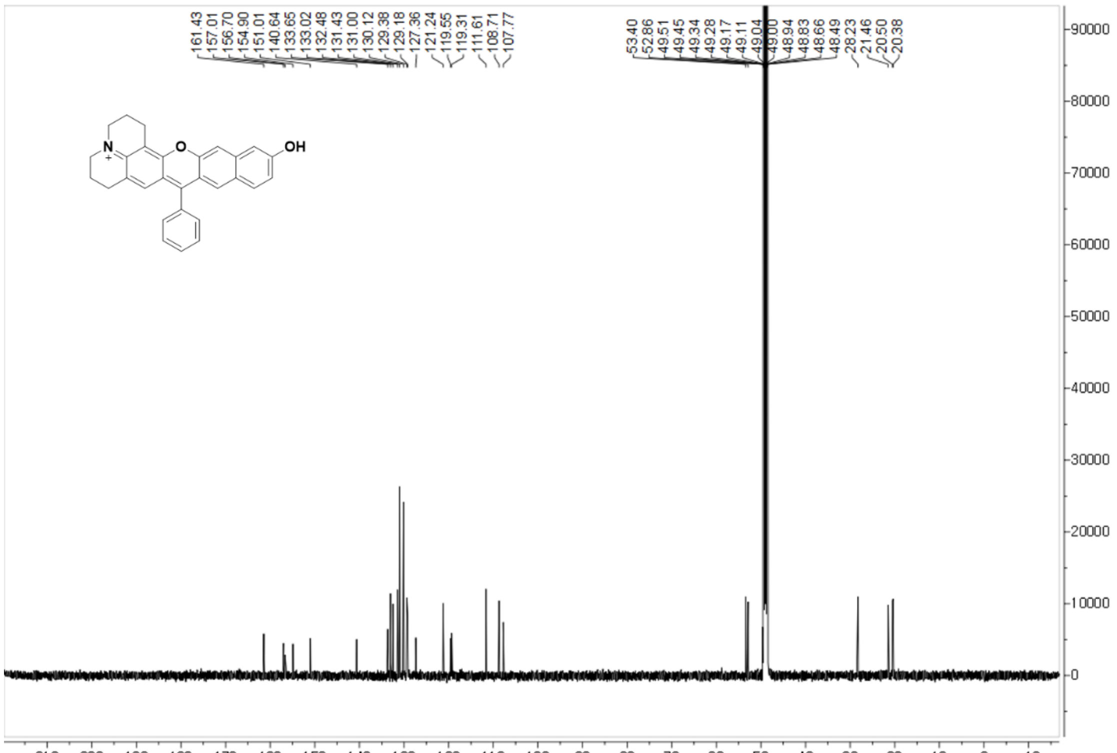

^{1}H NMR of **Br-BRosol**.

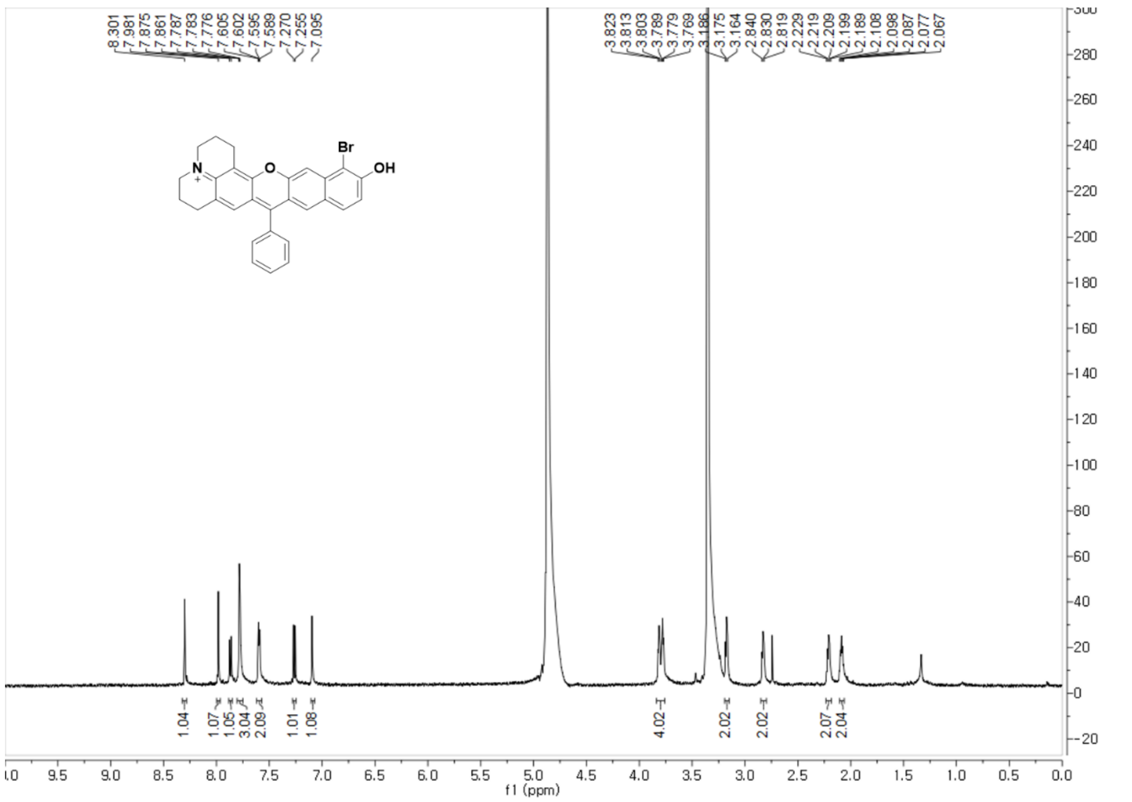

^{13}C NMR of **Br-BRosol**

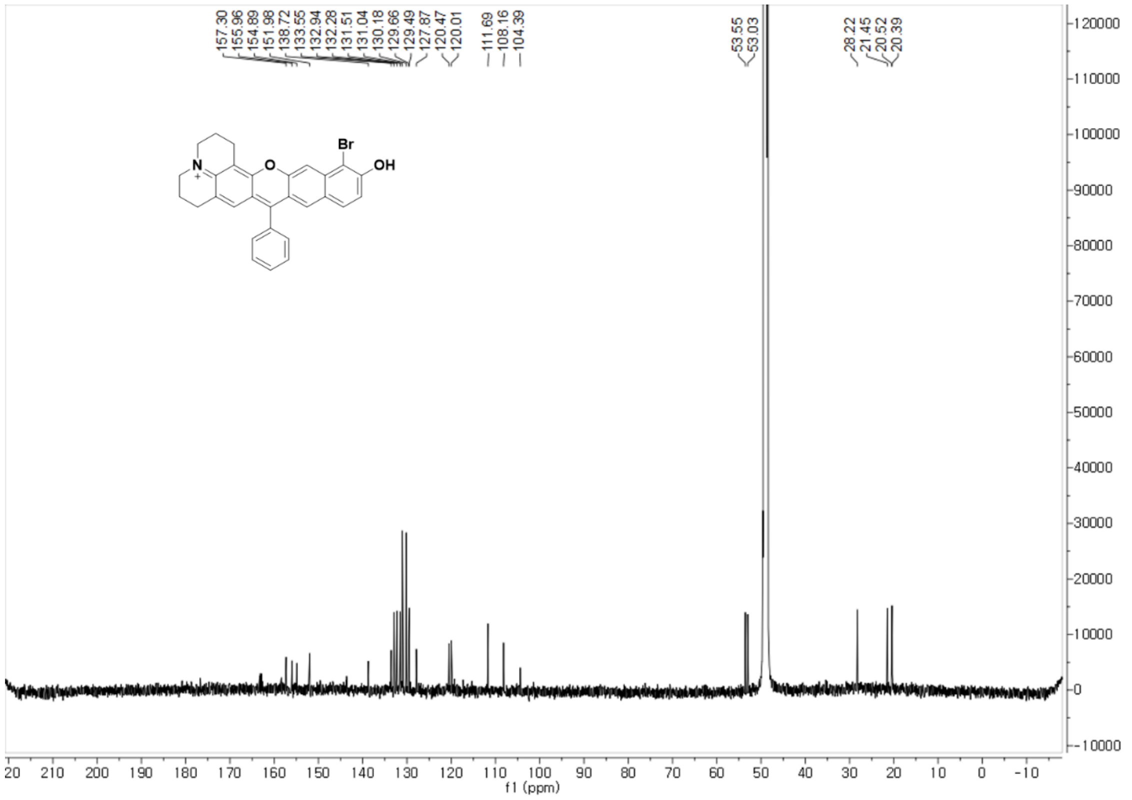

^{1}H NMR of **BRosol-P**.

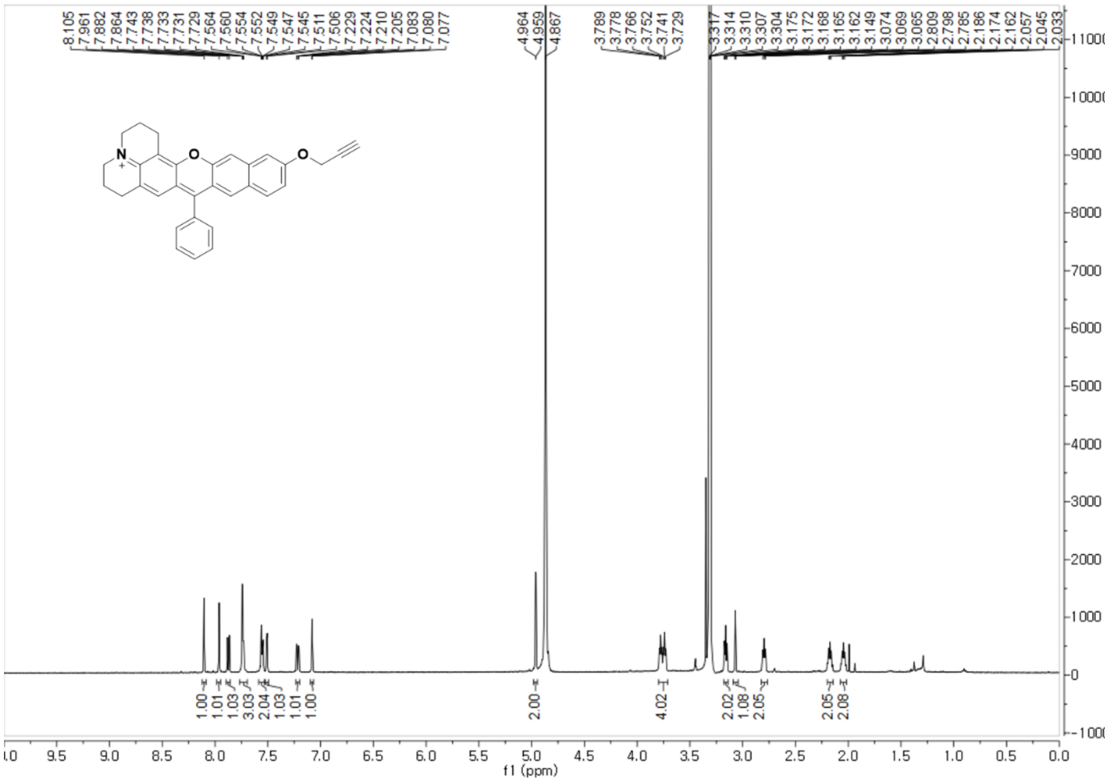

^{13}C NMR of **BRosol-P**.

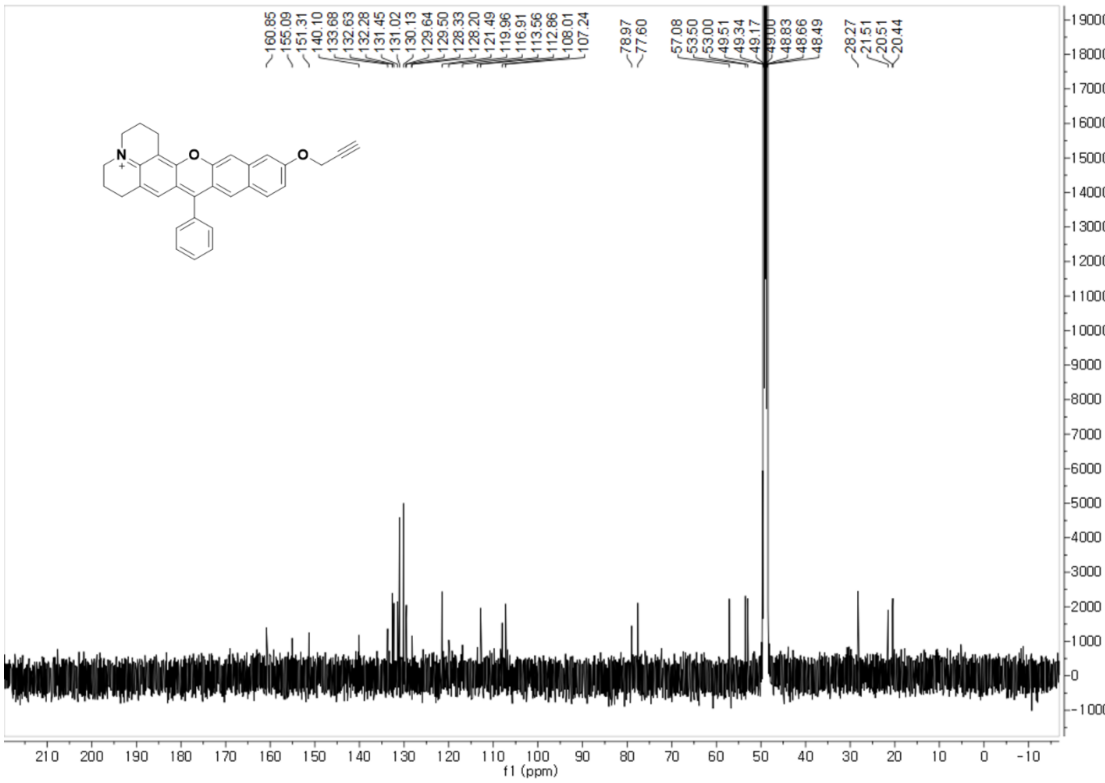

^{1}H NMR of **BRosol-E**.

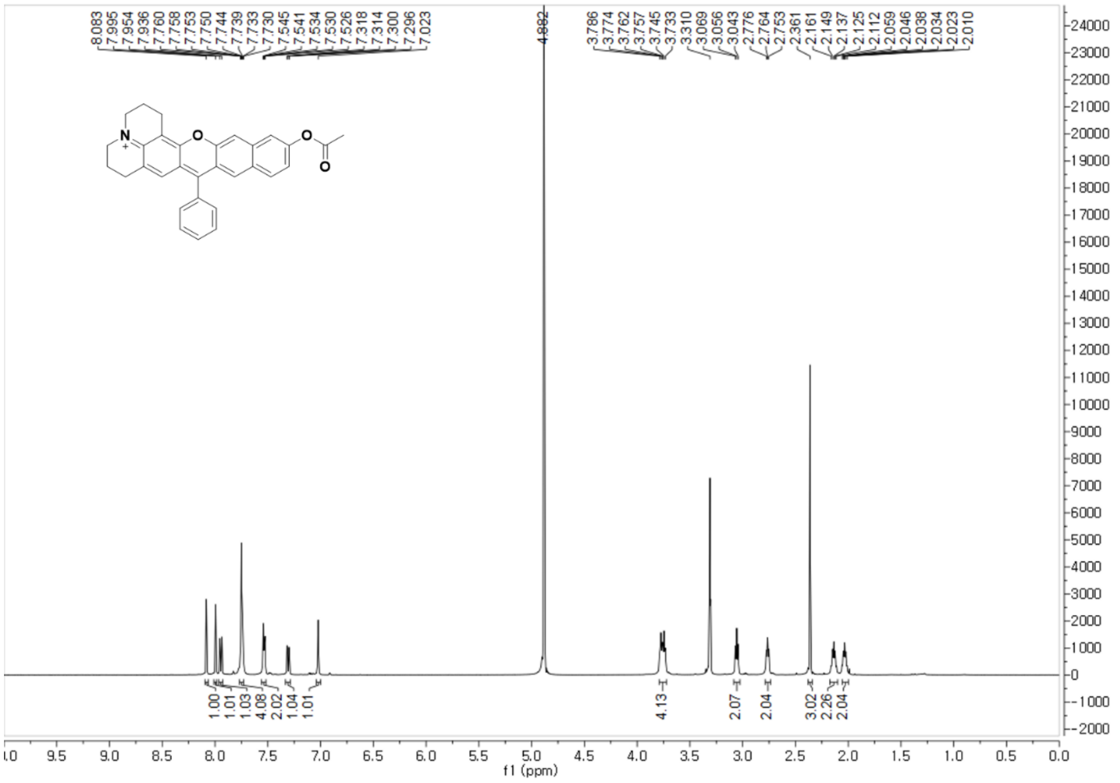

^{13}C NMR of **BRosol-E**.

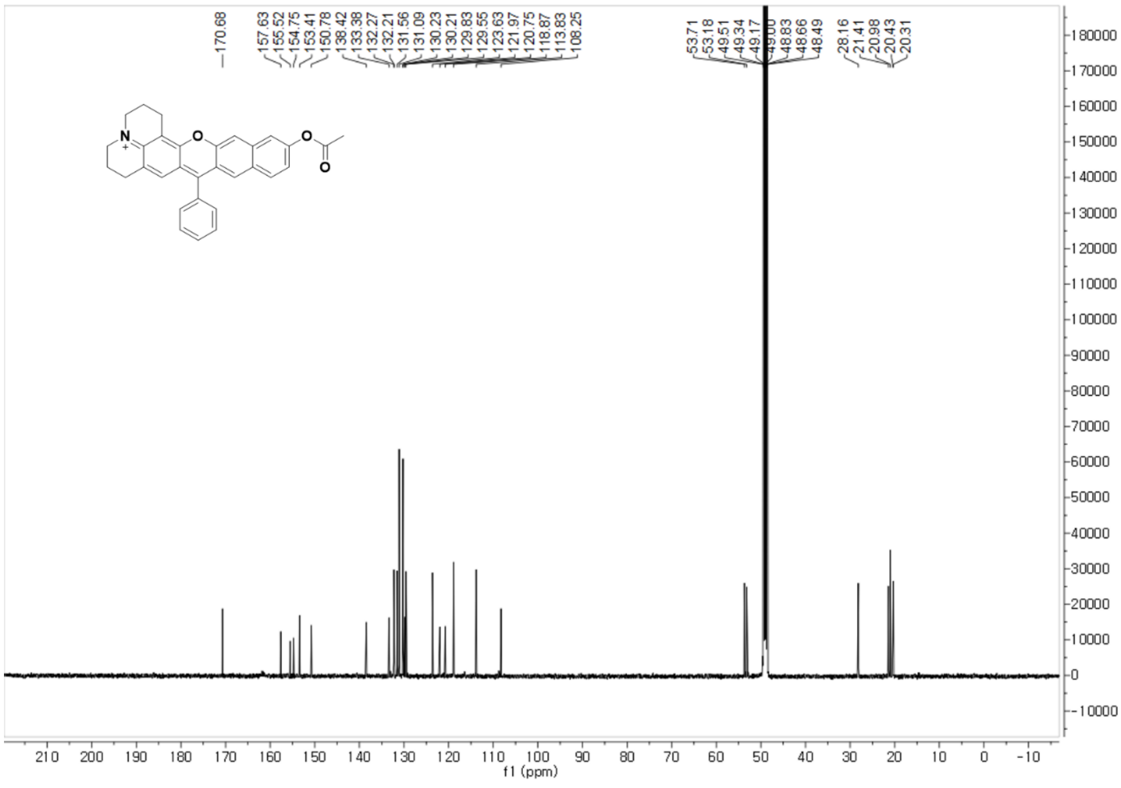

^{1}H NMR of **BRosol-DNP**.

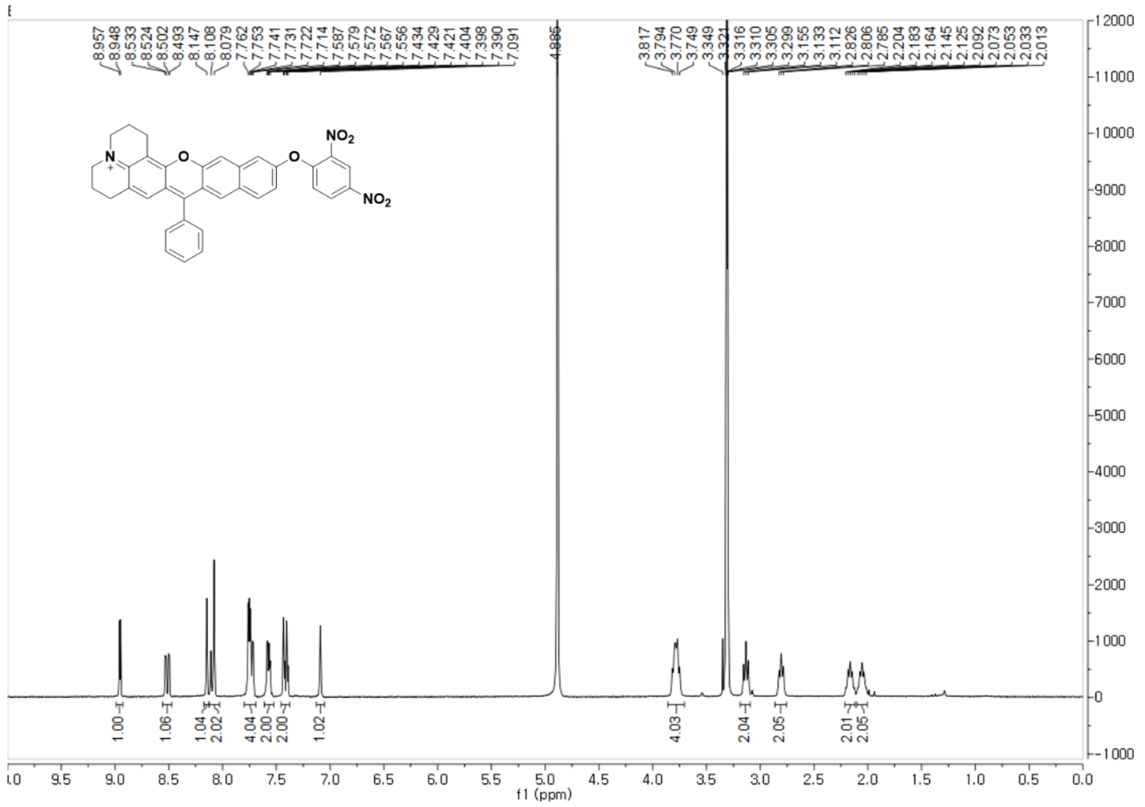

^{13}C NMR of **BRosol-DNP**.

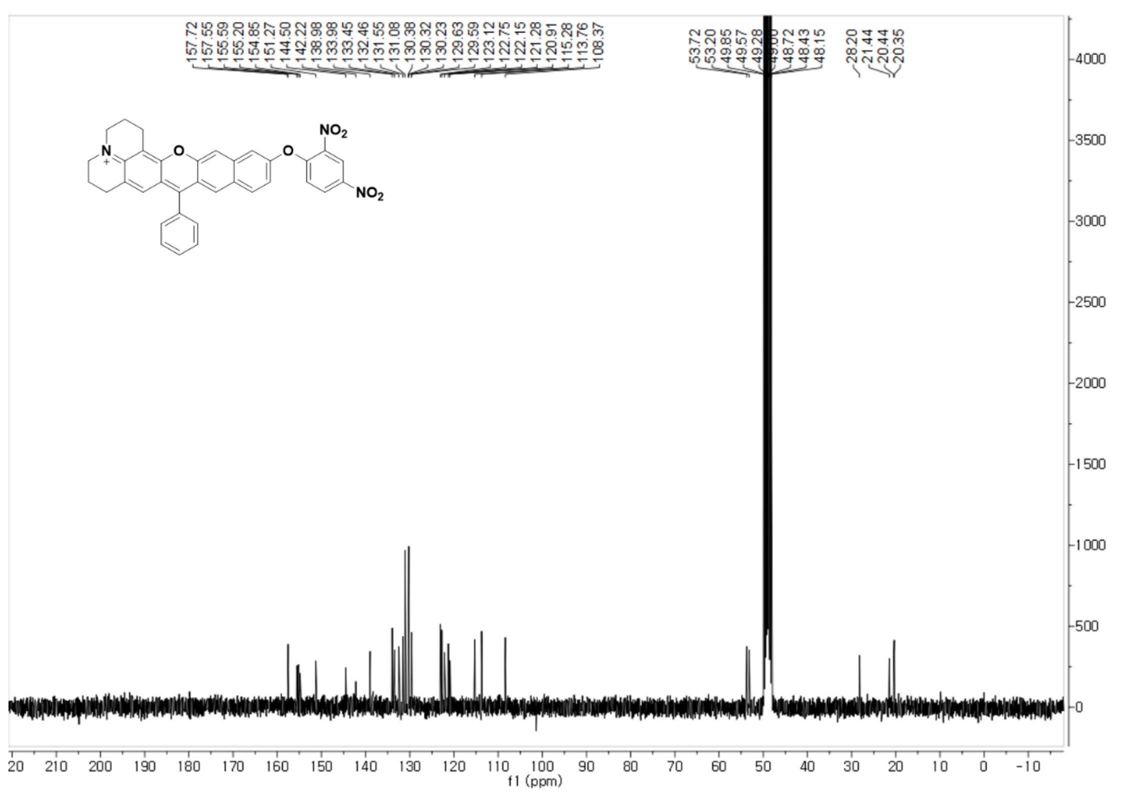

^{1}H NMR of **BRosol-NBE**.

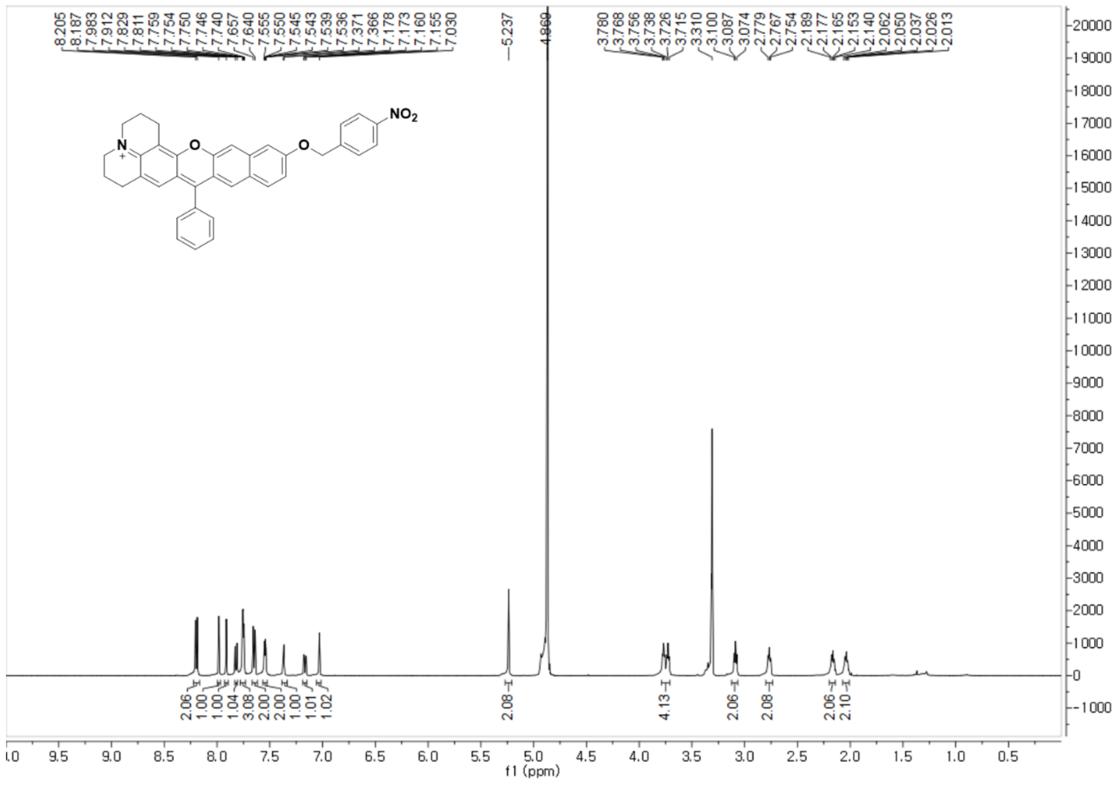

^{13}C NMR of **BRosol-NBE**.

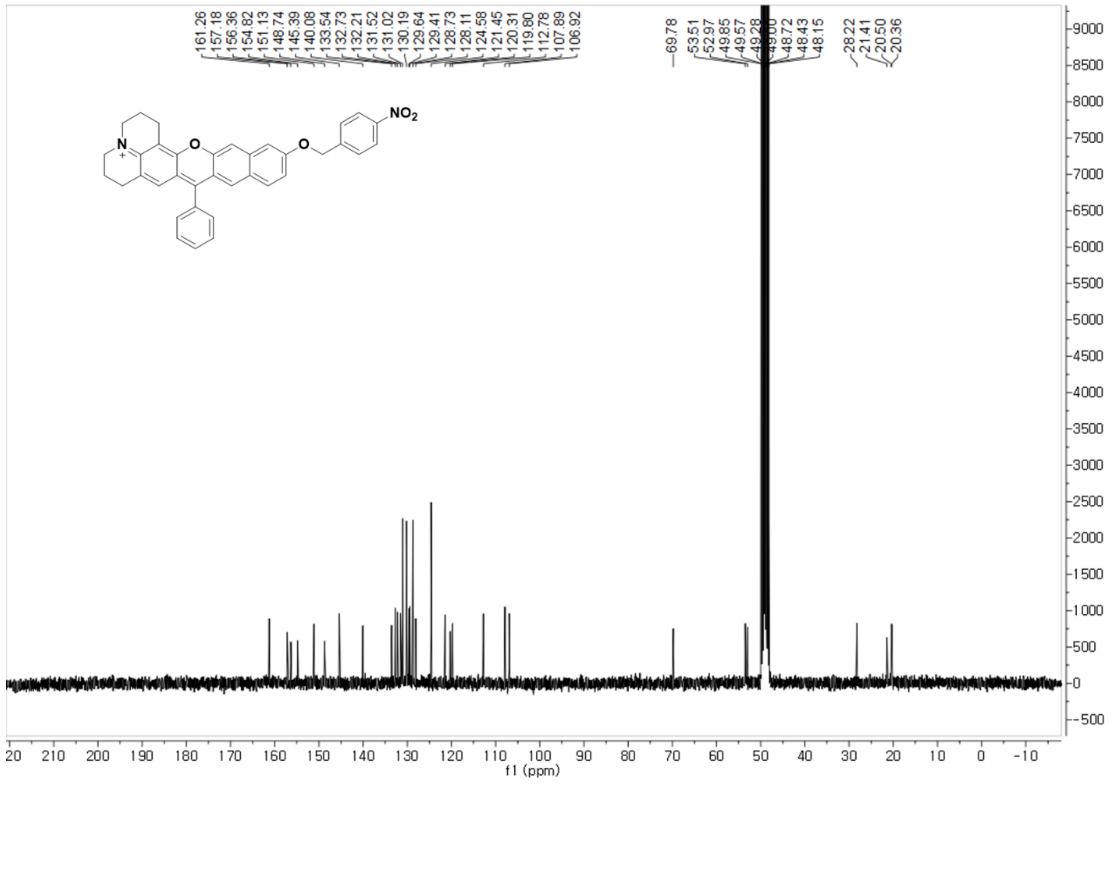

^{1}H NMR of **BRosol-triflate**.

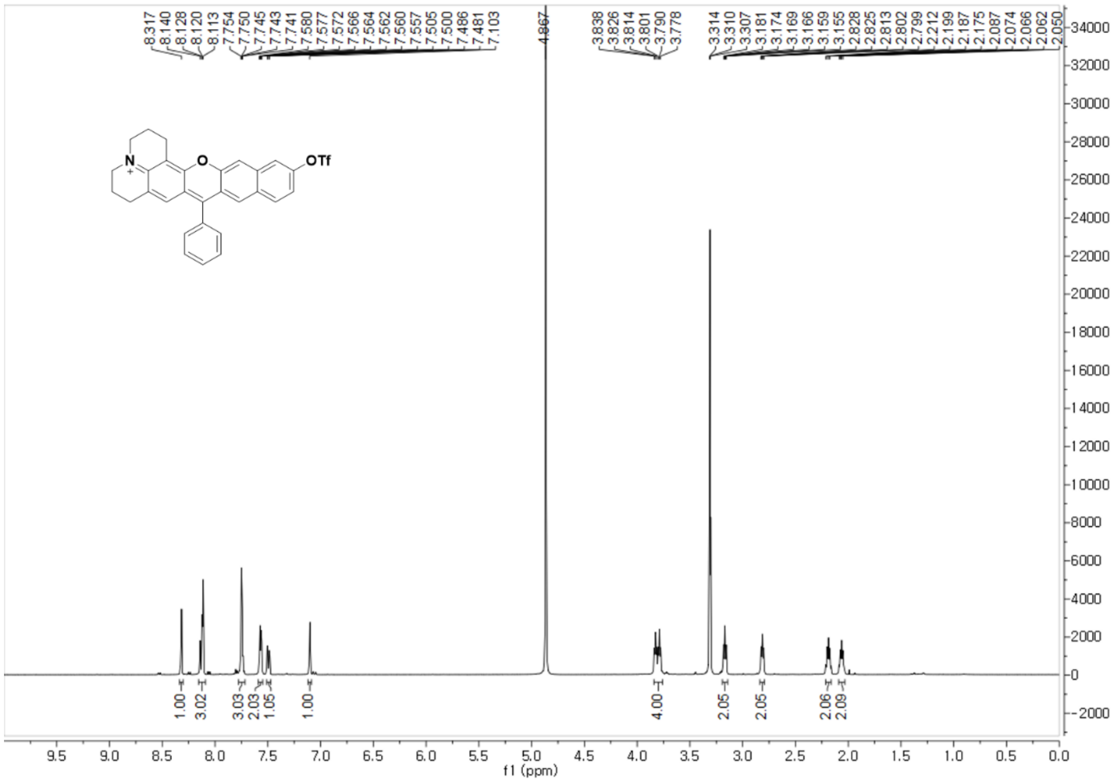

^{13}C NMR of **BRosol-triflate**.

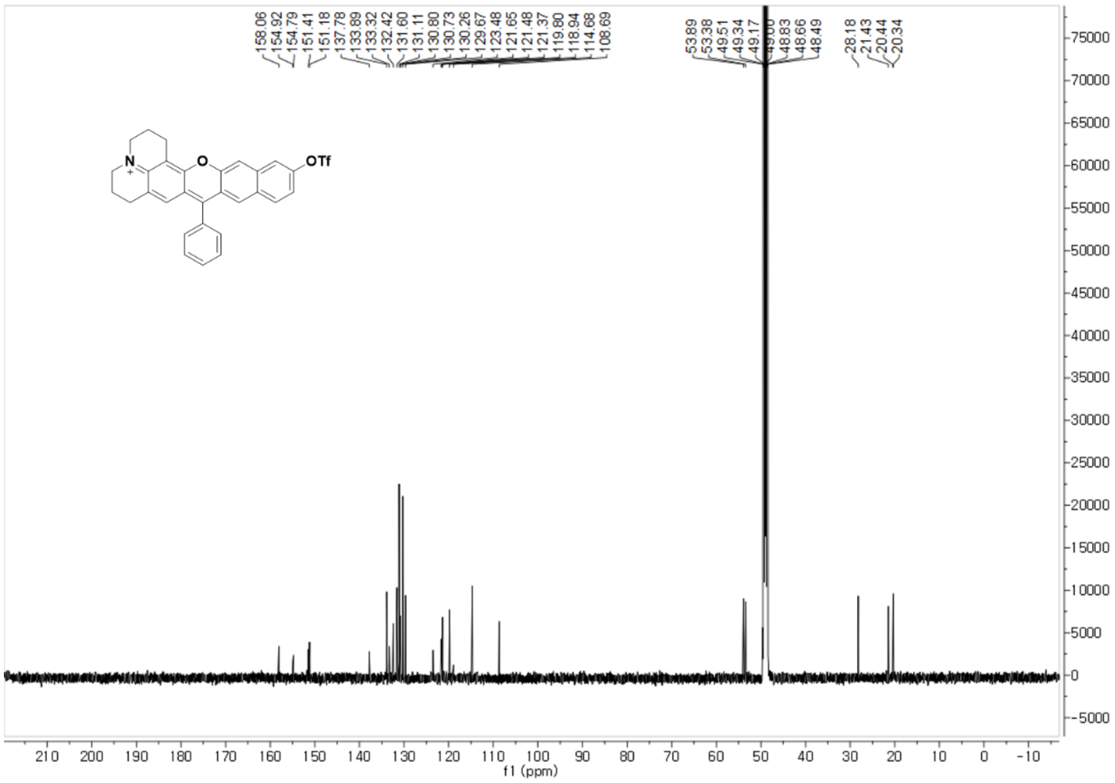

^{1}H NMR of **BRosam 1**.

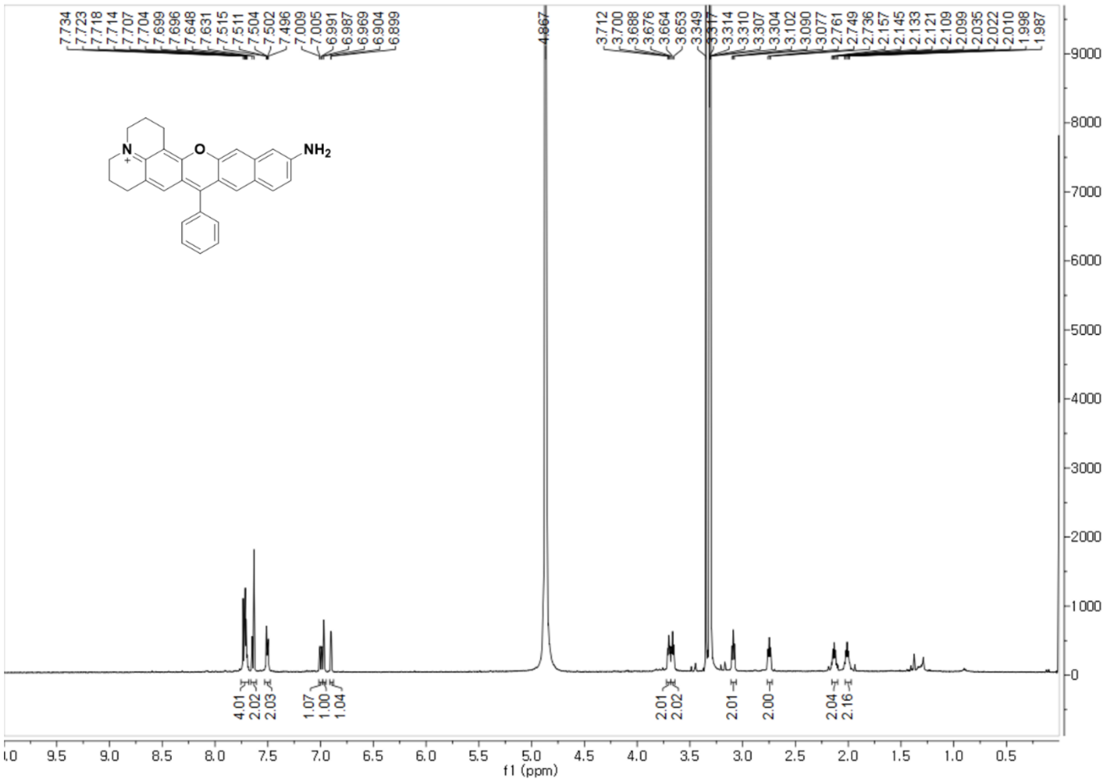

^{13}C NMR of **BRosam 1**.

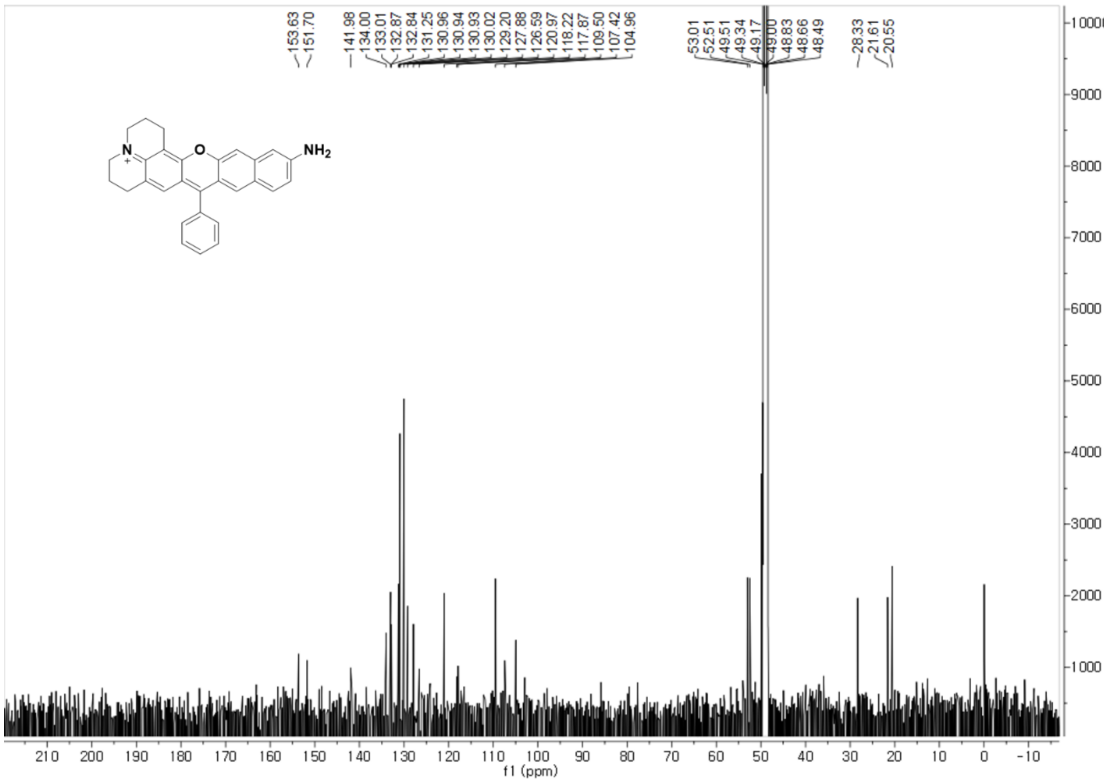

^{1}H NMR of **BRosam 2**.

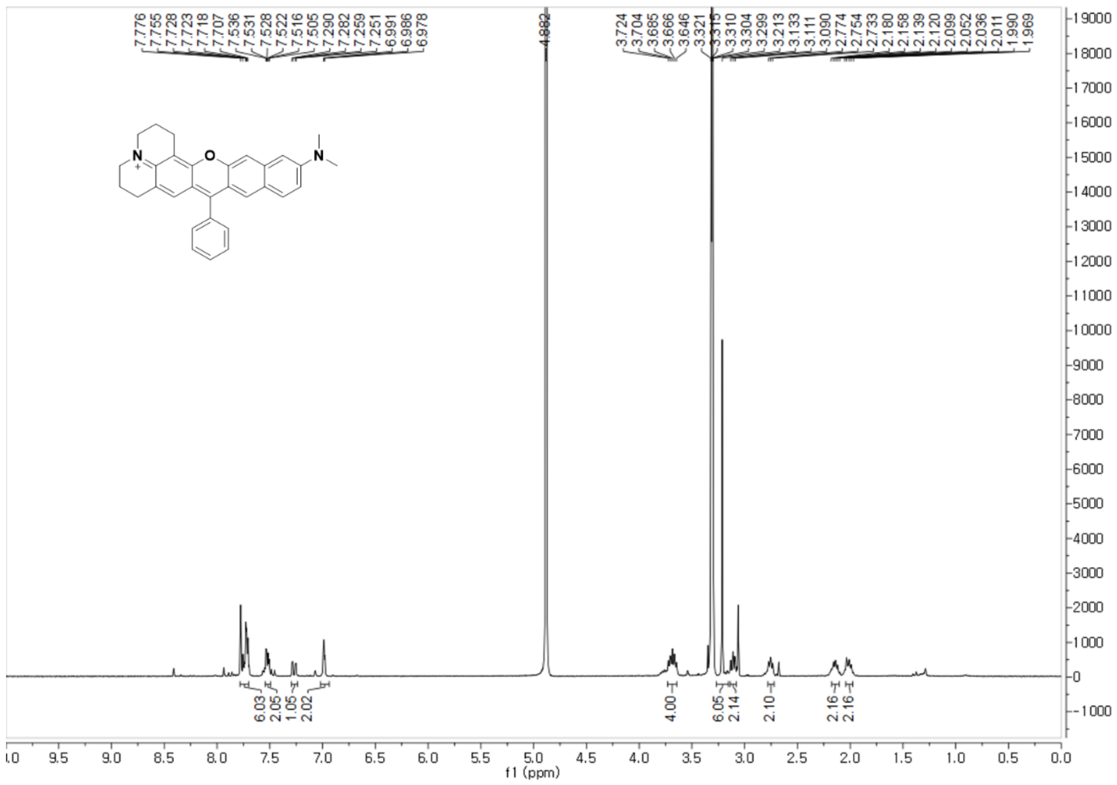

^{13}C NMR of **BRosam 2**.

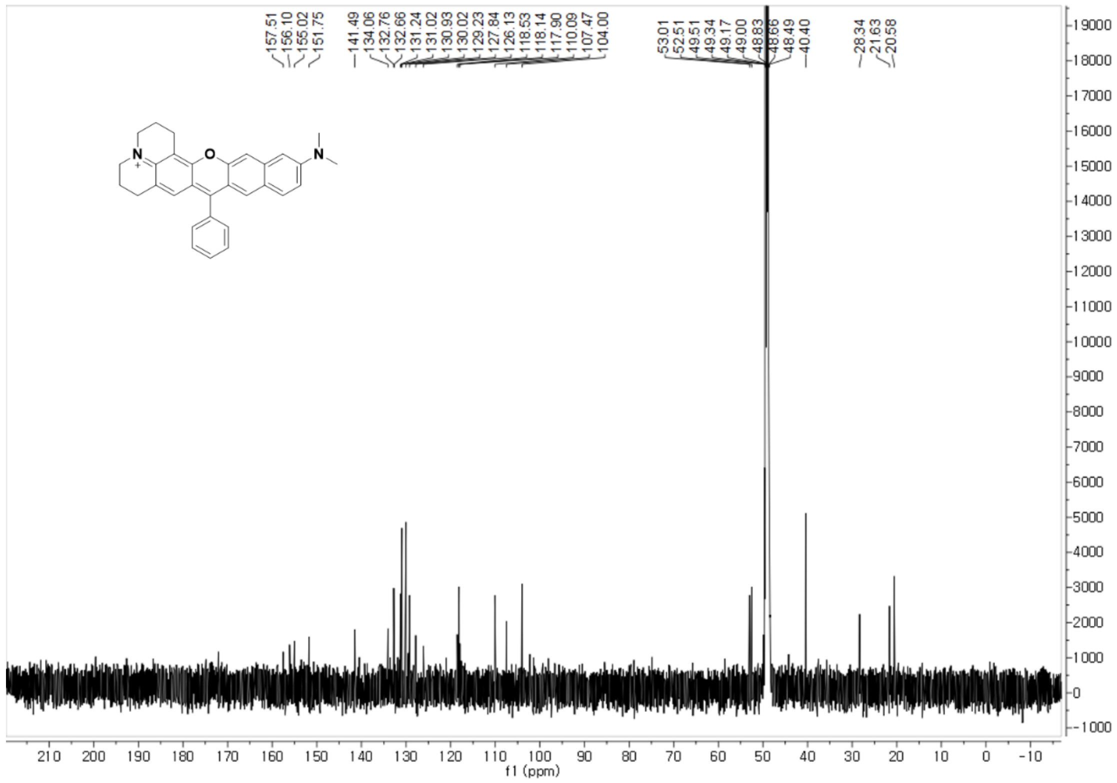

^{1}H NMR of **BRosam-pNBC**.

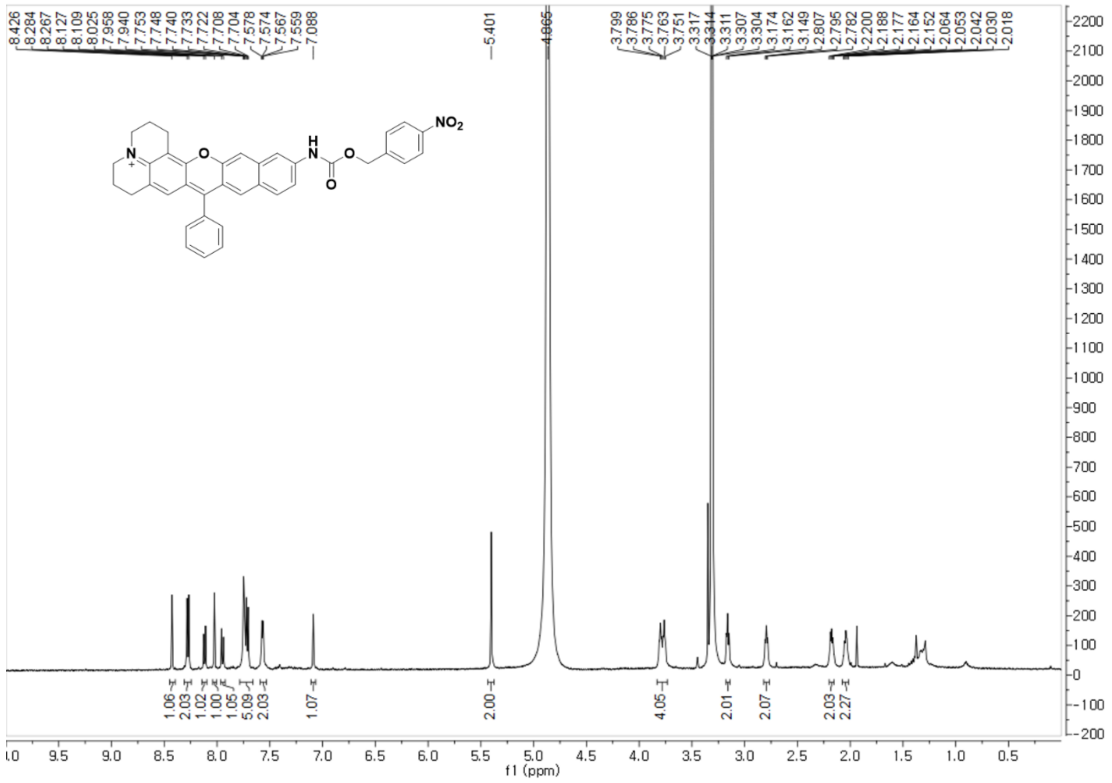

Figure S33. ^{13}C NMR of **BRosam-pNBC**.

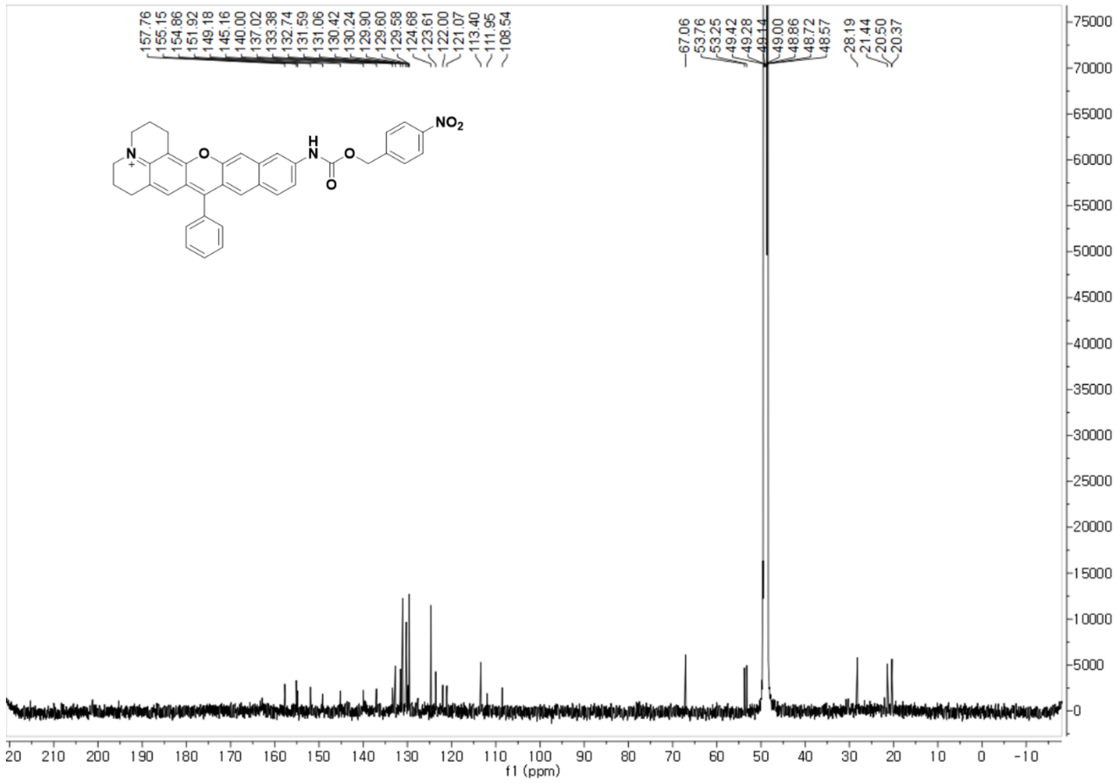

HRMS of **BRosol**.

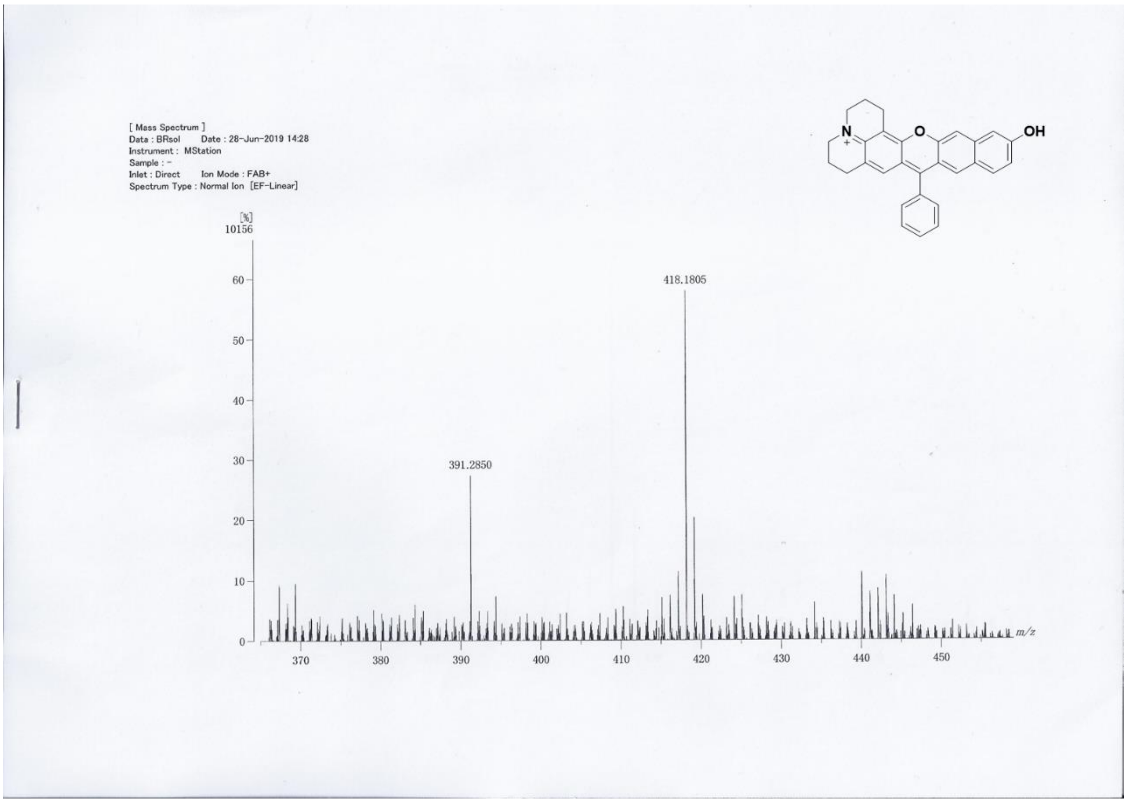

HRMS of **BRosol-P**.

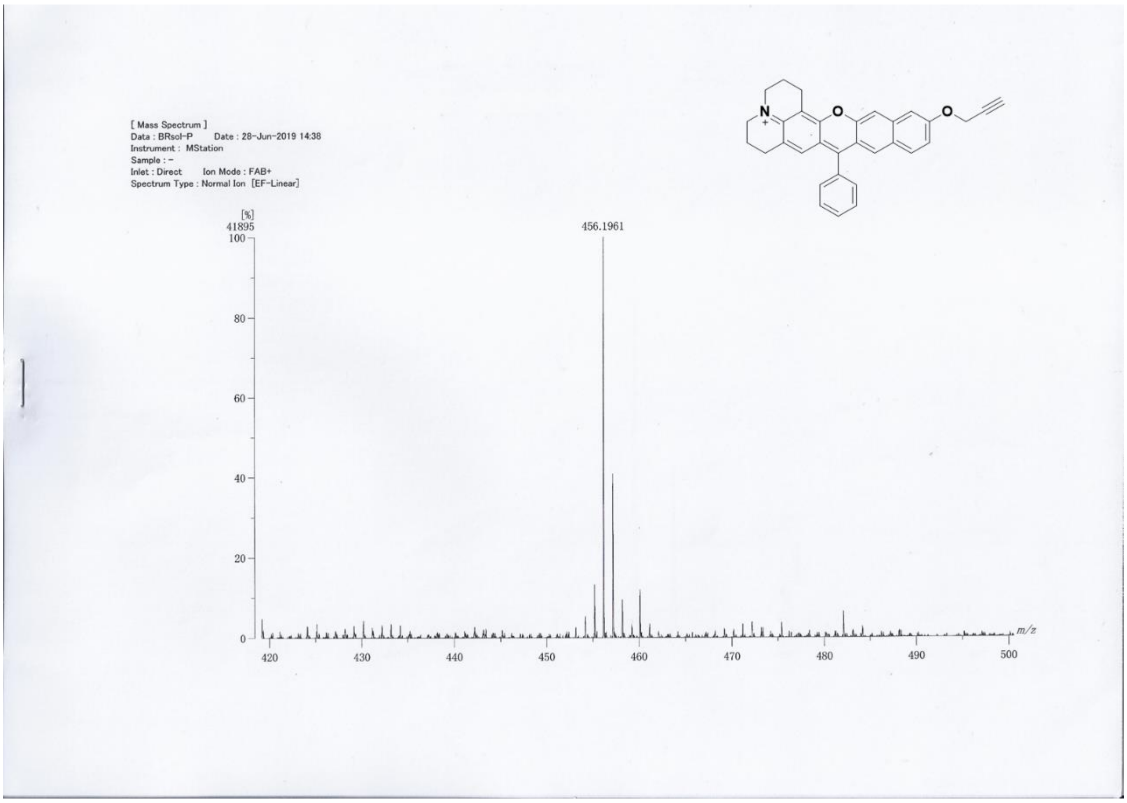

HRMS of **BRosol-E**.

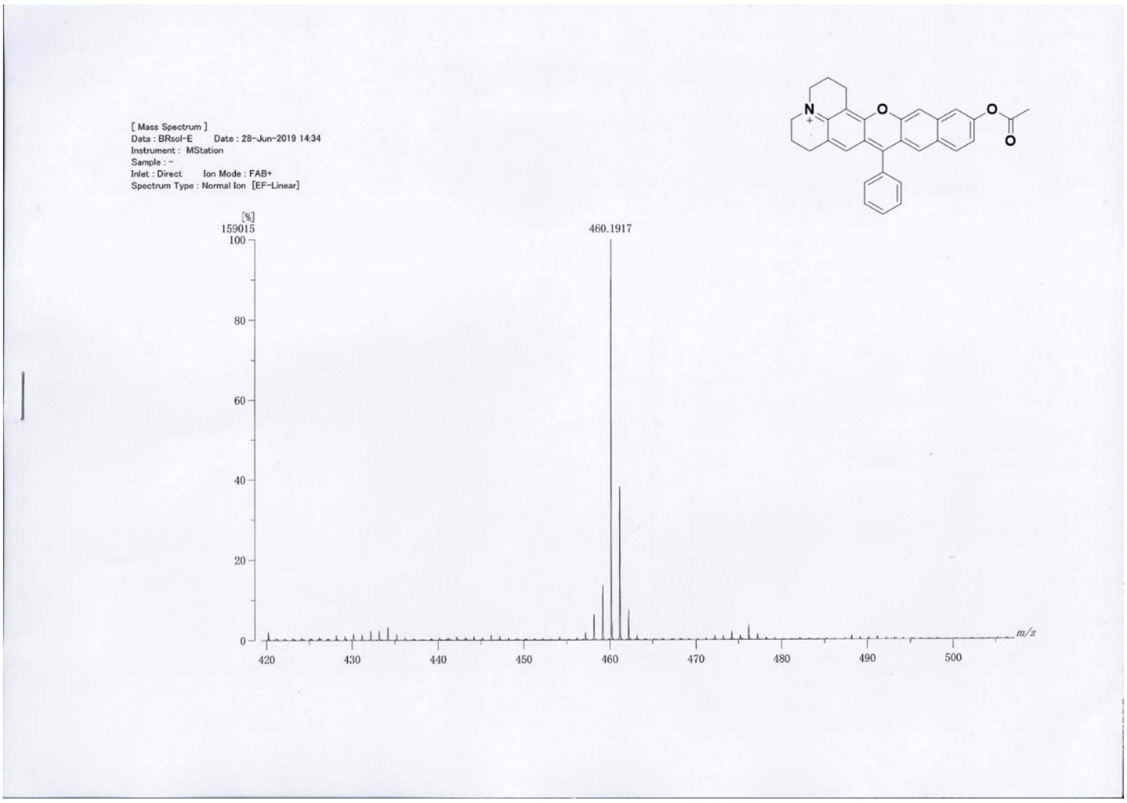

HRMS of **BRosol-DNP**.

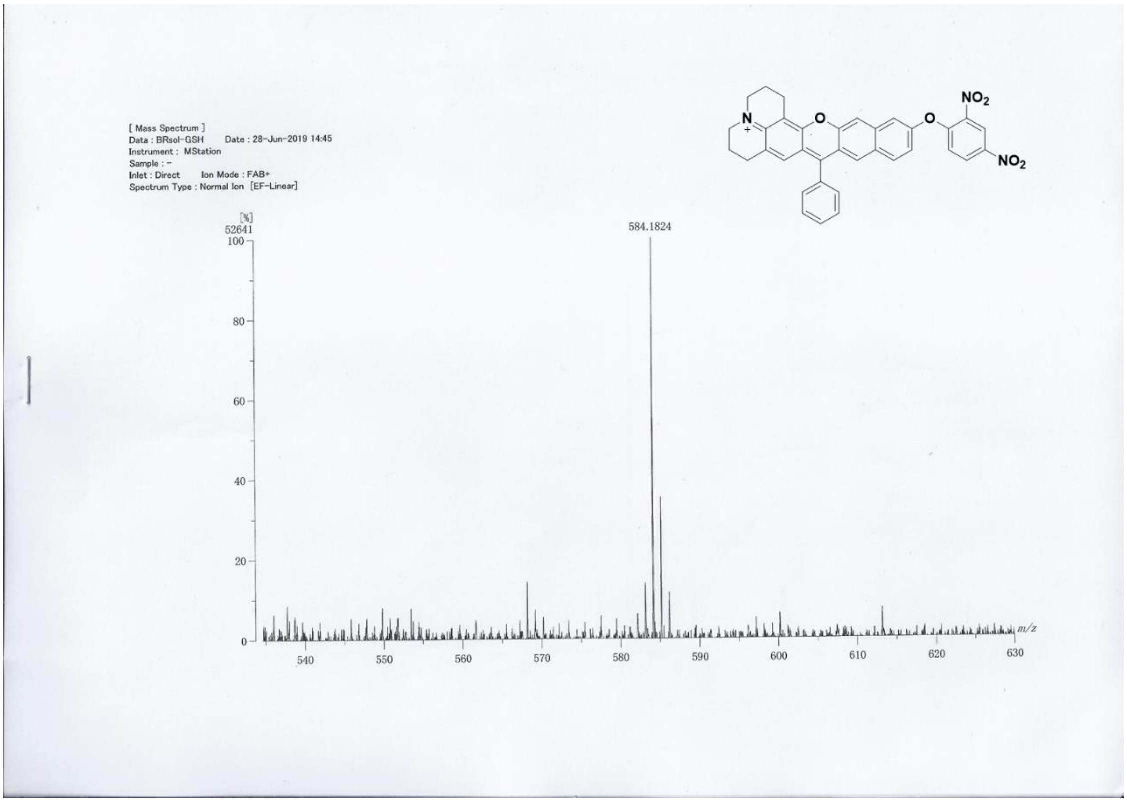

HRMS of **BRosol-NBE**.

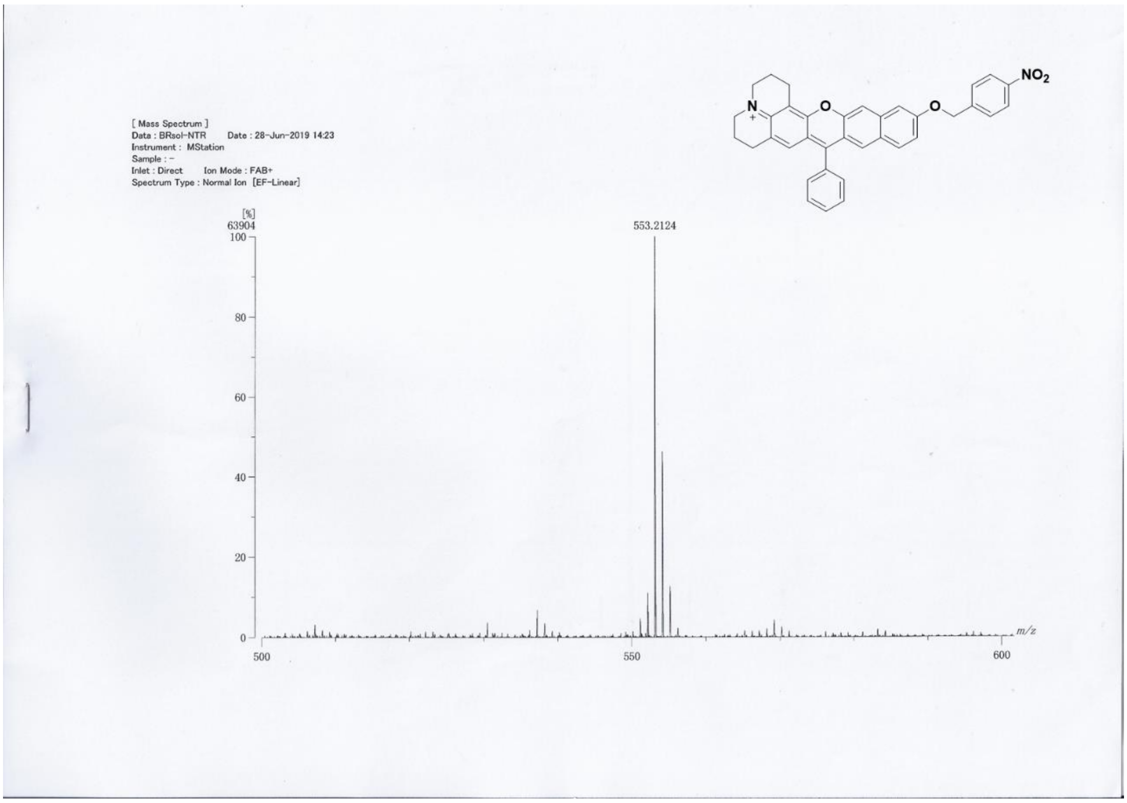

HRMS of **BRosol-triflate**.

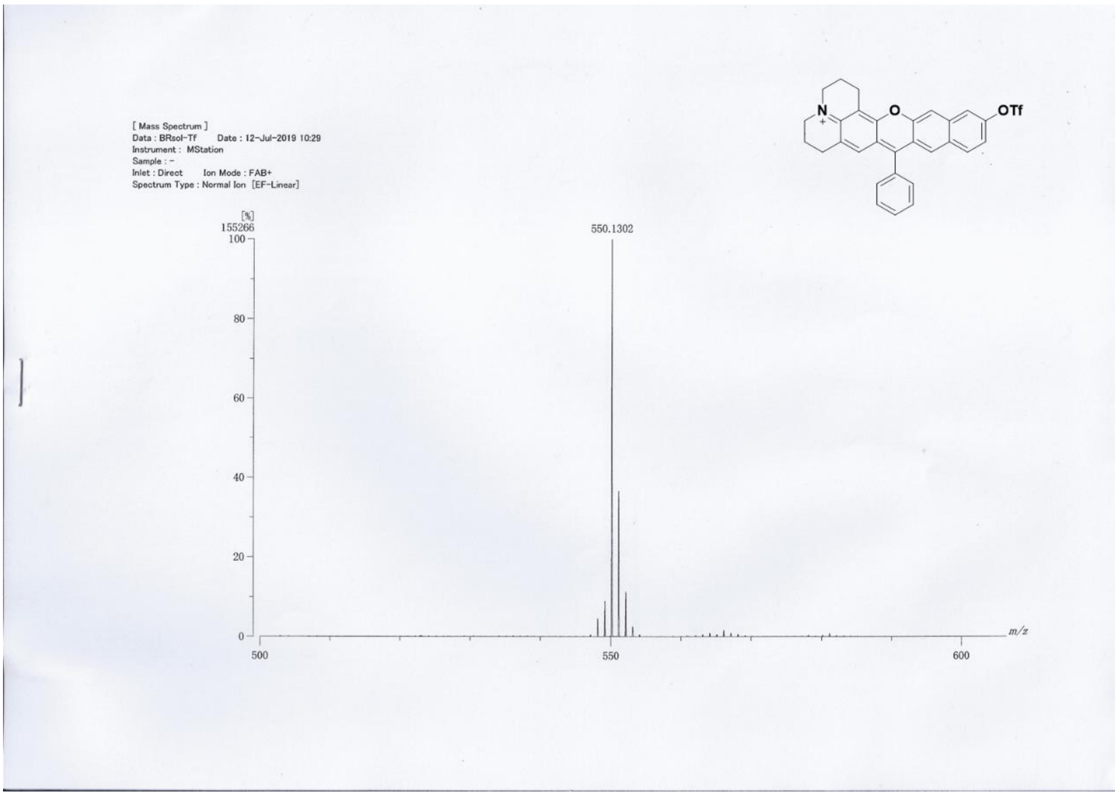

HRMS of **BRosam 1**.

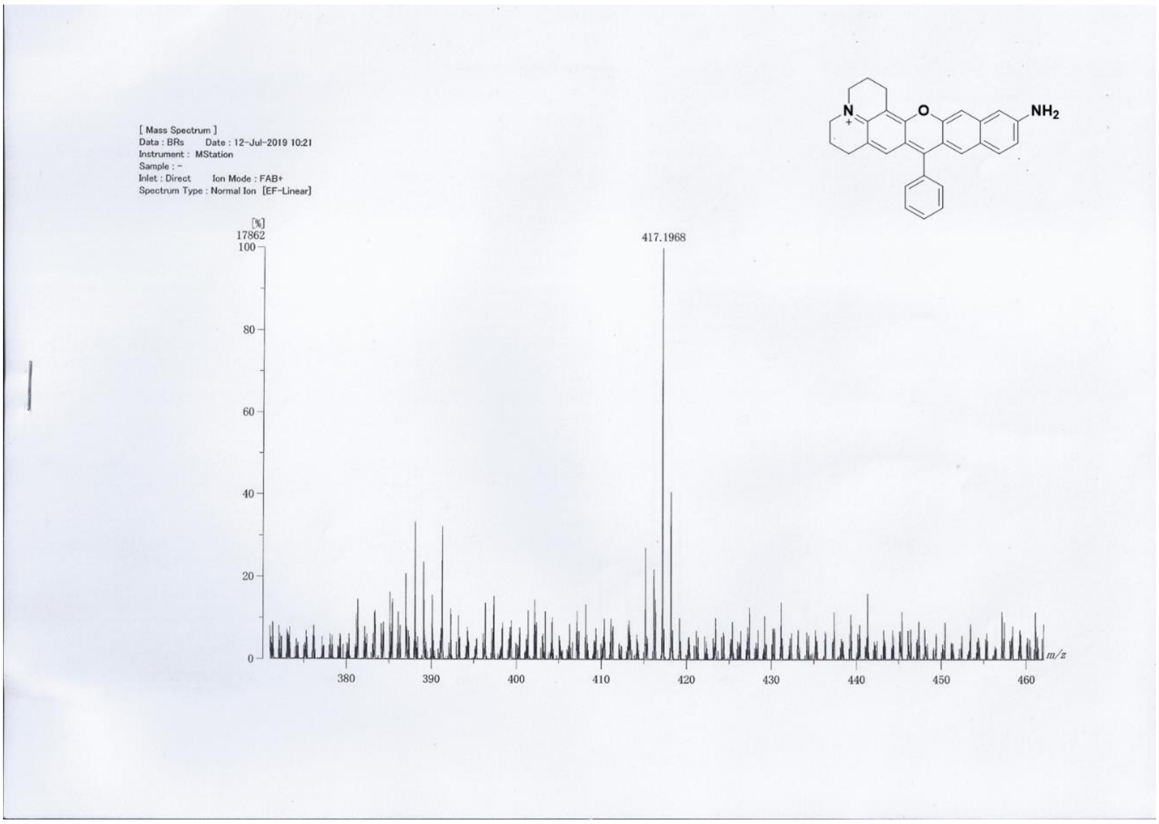

HRMS of **BRosam 2**.

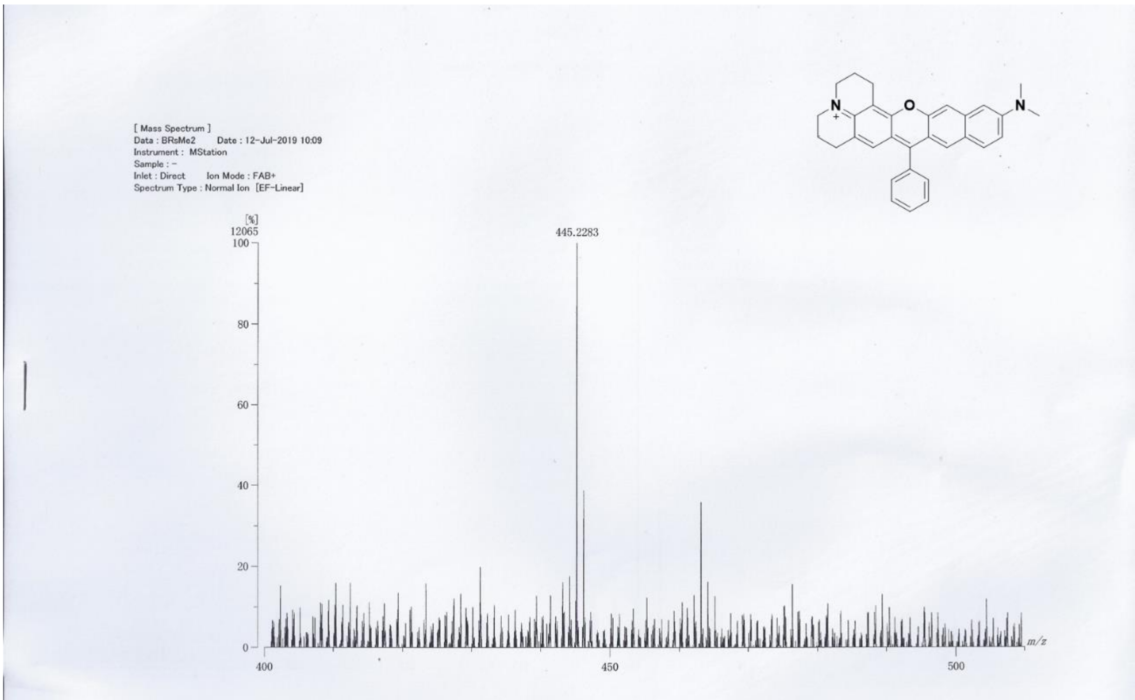

HRMS of **BRosam-pNBC**.

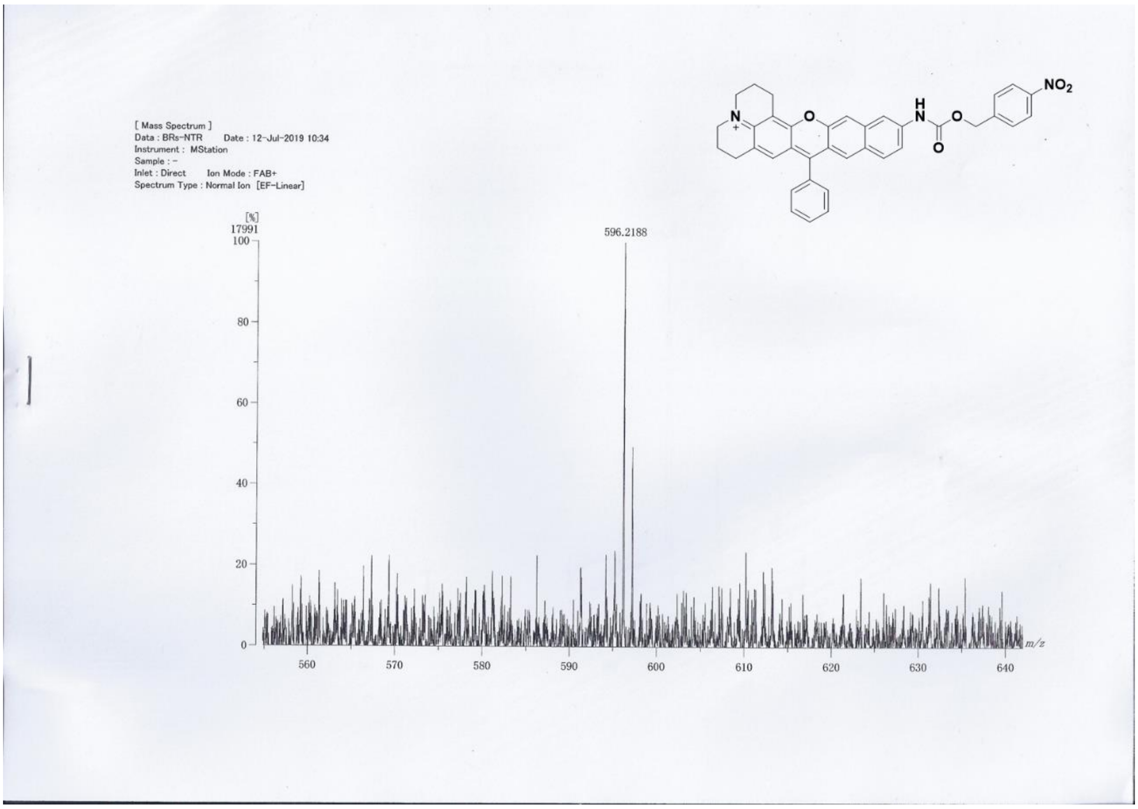

Supplement: SC-011-D0SC03314F-s001 [file SC-011-D0SC03314F-s001.pdf]
